# Independent evolution of plant natural products: Formation of benzoxazinoids in *Consolida orientalis* (Ranunculaceae)

**DOI:** 10.1016/j.jbc.2024.108019

**Published:** 2024-11-26

**Authors:** Matilde Florean, Hedwig Schultz, Jens Wurlitzer, Sarah E. O’Connor, Tobias G. Köllner

**Affiliations:** Max Planck Institute for Chemical Ecology, Department of Natural Product Biosynthesis, Jena, Germany

**Keywords:** benzoxazinoids, evolution, independent evolution, pathway, biosynthesis, defense compounds, DIBOA-Glc, plant biochemistry, plant defense, secondary metabolism

## Abstract

Benzoxazinoids (BXDs) are important defense compounds produced by a number of species from different, evolutionarily unrelated plant families. While BXD biosynthesis has been extensively studied in the grasses (monocots) and core eudicots, the mechanism of BXD synthesis in the basal eudicots is still unclear. We used an integrated metabolomics and transcriptomics approach to elucidate the BXD pathway in *Consolida orientalis*, a Ranunculaceae species known to produce the BXD DIBOA-Glc. Overexpression of candidate genes in *Nicotiana benthamiana* identified a flavin-dependent monooxygenase (*Co*BX2-3) and two cytochrome P450 enzymes (*Co*BX4 and *Co*BX5) that catalyze the oxidation steps that transform indole into DIBOA. Co-expression of *CoBx2-3*, *CoBx4*, and *CoBx5* with the previously described indole synthase gene *CoBx1* and the UDP-glucosyltransferase gene *CoBx8* in *N*. *benthamiana* resulted in the reconstitution of a fully active BXD pathway. The fact that *Co*BX2-3, *Co*BX4, and *Co*BX5 are not phylogenetically related to their counterparts in the grasses and core eudicots suggests independent evolution of benzoxazinoid biosynthesis in these three angiosperm lineages.

Benzoxazinoids (BXDs) are indole-derived specialized metabolites that have attracted considerable attention due to their diverse biological activities and fascinating evolutionary history. BXDs play multiple roles in plant defense, exhibiting antimicrobial, antifeedant, and allelopathic effects. In addition, BXDs can facilitate iron uptake in some plants, potentially promoting plant mineral nutrition (1, 2). BXDs are primarily found in the grasses (Poaceae) (1, 3, 4), a monocotyledonous angiosperm family, but also occur sporadically in a number of dicotyledonous species belonging to the Acanthaceae (5), Apocynaceae (6, 7), Lamiaceae (8, 9), Plantaginaceae (10), and Ranunculaceae (11) families (Fig. 1*A*).

**Figure 1:**
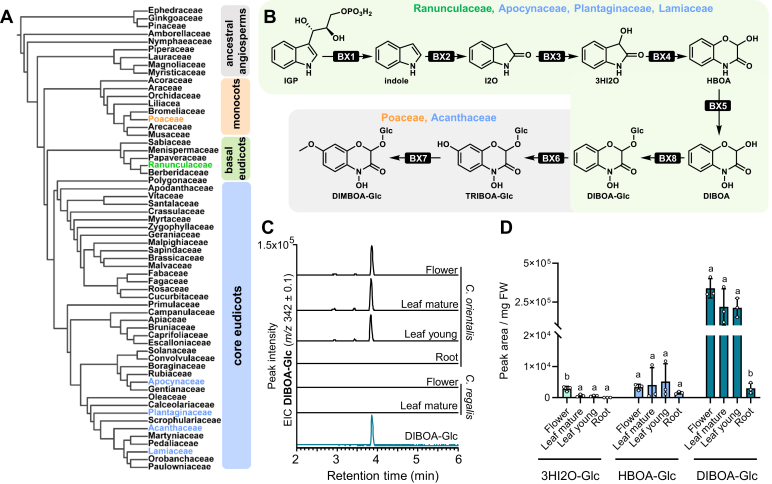
**Occurrence of benzoxazinoids in the angiosperms and metabolic profiling of *Consolida* species**. *A*, BXDs have been reported in the core eudicots, basal eudicots, and monocots. Plant families containing BXD-producing species are colored. The tree has been readapted from Kew gardens Tree of Life (32). *B*, biosynthetic pathway leading to the formation of BXDs in plants. Ranunculaceae, Apocynaceae, Plantaginaceae, and Lamiaceae produce DIBOA-Glc, while Poaceae and Acanthaceae can further convert DIBOA-Glc into the methoxylated derivative DIMBOA-Glc. *C*, *Consolida orientalis* but not the closely related *C*. *regalis* accumulates DIBOA-Glc. 100 ± 5 mg of plant tissue were extracted with methanol and analyzed through liquid chromatography-time of flight mass spectrometry (LC-qTOF-MS). Extracted ion chromatograms (EIC) are shown. *D*, accumulation of BXDs in different organs of *C*. *orientalis*. 100 ± 5 mg of leaf tissue were extracted with methanol and analyzed through LC-qTOF-MS. EIC are shown. Means and SD are displayed (n = 3 biological replicates, independent plants). Columns labeled with different letters represent statistically significant differences for each compound among tissues (*p* < 0.05, one-way ANOVA with Tukey’s correction for multiple comparisons). Detailed statistical values for each comparison are reported in Supplemental Dataset 1.

Decades of maize research (*Zea mays*, Poaceae) have shown that the BXD pathway starts with the formation of indole from indole-3-glycerol phosphate through the action of the indole 3-glycerol phosphate lyase (IGL) *Zm*BX1 (3, 12). Indole then undergoes a series of sequential oxidations catalyzed by four evolutionarily related cytochrome P450 monooxygenases (CYP). These CYPs belong to the CYP71 C subfamily and are named *Zm*BX2, *Zm*BX3, *Zm*BX4, and *Zm*BX5, respectively (3). *Zm*BX2 converts indole to indolin-2-one (I2O), which is further metabolized by *Zm*BX3 to 3-hydroxyindolin-2-one (3HI2O). *Zm*BX4 catalyzes the oxidative ring expansion of 3HI2O that leads to 2-hydroxy-3,4-dihydro-1,4-benzoxazin-3-one (HBOA) (13). Finally, *Zm*BX5 mediates the *N*-hydroxylation of HBOA to 2,4-dihydroxy-1,4-benzoxazin-3-one (DIBOA). DIBOA is then glucosylated by the UDP-glucosyltransferases (UGTs) *Zm*BX8 and *Zm*BX9, which quench BXD aglucone reactivity and promote BXD storage as stable glucosides in the vacuole (3, 14). DIBOA-Glc has significant defense properties and is the end product of the BXD pathway in several grasses and most dicotyledonous species (Fig. 1*B*) that produce BXDs (1, 8, 15). In other species such as maize and the eudicot *Aphelandra squarrosa*, DIBOA-Glc can undergo a further hydroxylation catalyzed by the 2-oxoglutarate-dependent dioxygenase (2-ODD) BX6. The resulting product 2-(2,4,7-trihydroxy-1,4-benzoxazine-3-one)-β-D-glucopyranose (TRIBOA-Glc) is methylated to 2-(2,4-dihydroxy-7-methoxy-1,4-benzoxazin-3-one)-β-D-glucopyranose (DIMBOA-Glc) by the *O*-methyltransferase (OMT) BX7 (Fig. 1*B*) (16, 17). While the biosynthesis of BXDs in maize and other grasses has been extensively studied, the formation of BXDs in eudicots has only recently been elucidated. Elucidation of the BXD pathway in Acanthaceae and Lamiaceae species demonstrated that BXDs have evolved independently in these two eudicot families (9, 17). The studied species, *A*. *squarrosa*, *Acanthus ilicifolius* (both Acanthaceae), and *Lamium galeobdolon* (Lamiaceae), produced indole through the expression of a pseudoenzyme (18). In addition, these species were shown to possess a flavin-dependent monooxygenase (FMO) that performs the functions of *Zm*BX2 and *Zm*BX3 from maize. Furthermore, the P450 enzymes BX4 and BX5 identified in these species belong to different (sub)families, indicating a parallel evolution of their enzymatic activities (19).

While most of the BXD-producing species in the eudicots belong to core eudicot families, a single species in the Ranunculaceae, a basal eudicot family (20) has also been shown to accumulate BXDs (Fig. 1*A*). This species, *Consolida orientalis*, produces high levels of DIBOA-Glc in leaves and flowers and the first and last enzymes of its BXD pathway, the IGL *Co*BX1 and the UGT *Co*BX8, respectively, have already been reported (8, 9, 11). Sequence comparisons showed that *Co*BX1 and *Co*BX8 are evolutionarily unrelated to maize *Zm*BX1 and *Zm*BX8, which led to the hypothesis that BXDs evolved independently in monocots and *C*. *orientalis* (9, 11). However, the question of whether the BXD pathway in the basal eudicot *C*. *orientalis* and other BXD-producing core eudicot species has a common evolutionary origin remained unresolved.

Here, we used an integrated metabolomics and transcriptomics approach to elucidate the missing steps of BXD biosynthesis in *C*. *orientalis*. By expressing the maize BXD pathway in *Nicotiana benthamiana* and replacing individual maize BXD genes with candidate genes from *C*. *orientalis*, we identified one FMO and two P450 enzymes involved in BXD biosynthesis in this species. Sequence comparisons of these newly discovered enzymes showed that the BXD pathway has evolved independently in the basal and core eudicots.

## Results

### BXDs accumulate in aerial organs of *C*. *orientalis* but not in *C*. *regalis*

Using liquid chromatography-quadrupole time-of-flight mass spectrometry (LC-qTOF-MS) we performed untargeted metabolomic analysis of methanolic extracts from young and mature leaves, flowers, and roots. We confirmed that BXDs accumulate primarily in aerial organs of *C*. *orientalis*, as previously reported (9). The organ with the highest BXD content was flowers, followed by young and mature leaves, while only low levels of BXDs were detectable in roots (Fig. 1, *C* and *D* and Fig. S1, *B* and *C*). *Consolida regalis*, a species closely related to *C*. *orientalis*, did not appear to produce BXDs, again consistent with previous reports (21, 22) (Figs. 1*C* and S1*A*).

### Previously identified *Co*BX1 and *Co*BX8 show different levels of activity

**Figure 2:**
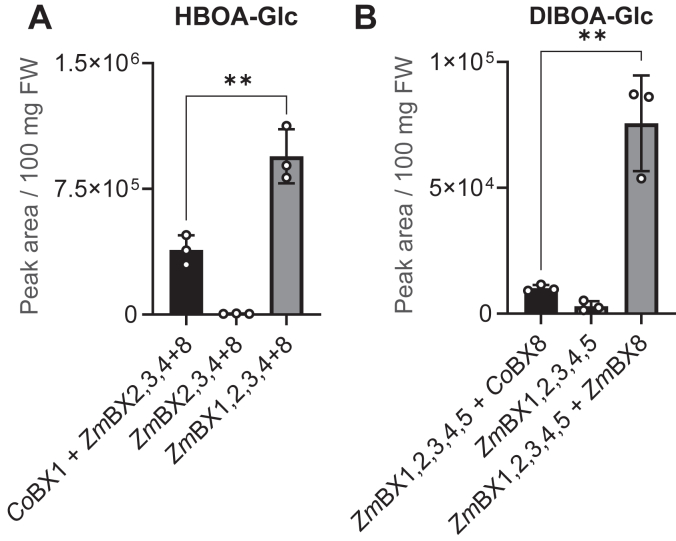
**Expression of previously identified *CoBx1* and *CoBx8* in *Nicotiana benthamiana***. Activity of *Co*BX1 (*A*) and *Co*BX8 (*B*) *in N*. *benthamiana* in combination with BXD biosynthetic genes from maize. 100 ± 5 mg of leaf tissue was extracted with methanol and analyzed using LC-qTOF-MS. Means and SD are shown (n = 3 biological replicates, independent plants). Unpaired *t* test was used to evaluate differences among samples, (A) *p* = 0.0062, t = 5.261, df = 4; (B) *p* = 0.004, t = 5.955, df = 4.

Two BXD biosynthetic enzymes from *C*. *orientalis*, the IGL *Co*BX1 and the UGT *Co*BX8, have been reported (9, 11). However, while *Co*BX1 exhibited kinetic parameters comparable to those of maize *Zm*BX1 (9), the UGT *Co*BX8 was approximately 10 times less active than *Zm*BX8 when assayed *in vitro* (11). To test whether *Co*BX1 and *Co*BX8 are active in planta, we expressed these genes together with maize BXD genes in *N*. *benthamiana*. Co-expression of *CoBx1* together with *ZmBx2*, *3*, *4*, and *ZmBx8* and co-expression of *CoBx8* together with *ZmBx2*, *3*, *4*, and 5 resulted in lower levels of HBOA-Glc and DIBOA-Glc compared to the expression of the corresponding *Z*. *mays* genes (Fig. 2). When *CoBx1* was expressed, HBOA-Glc was produced in comparable orders of magnitude to those of a control experiment using *ZmBx1* in place of *CoBx1* (Fig. 2*A*). However, the substitution of *ZmBx8* with *CoBx8* resulted in levels of DIBOA-Glc almost 10 times lower than those produced by *Zm*BX8 (Fig. 2*B*). Although *CoBx8* expression levels in *N*. *benthamiana* were not measured, these results suggest that *CoBx8* could have lower catalytic efficiency both *in vitro* and *in planta*.

### Comparative transcriptomics of different organs of *C*. *orientalis* and *C*. *regalis* revealed candidate BXD genes

**Figure 3:**
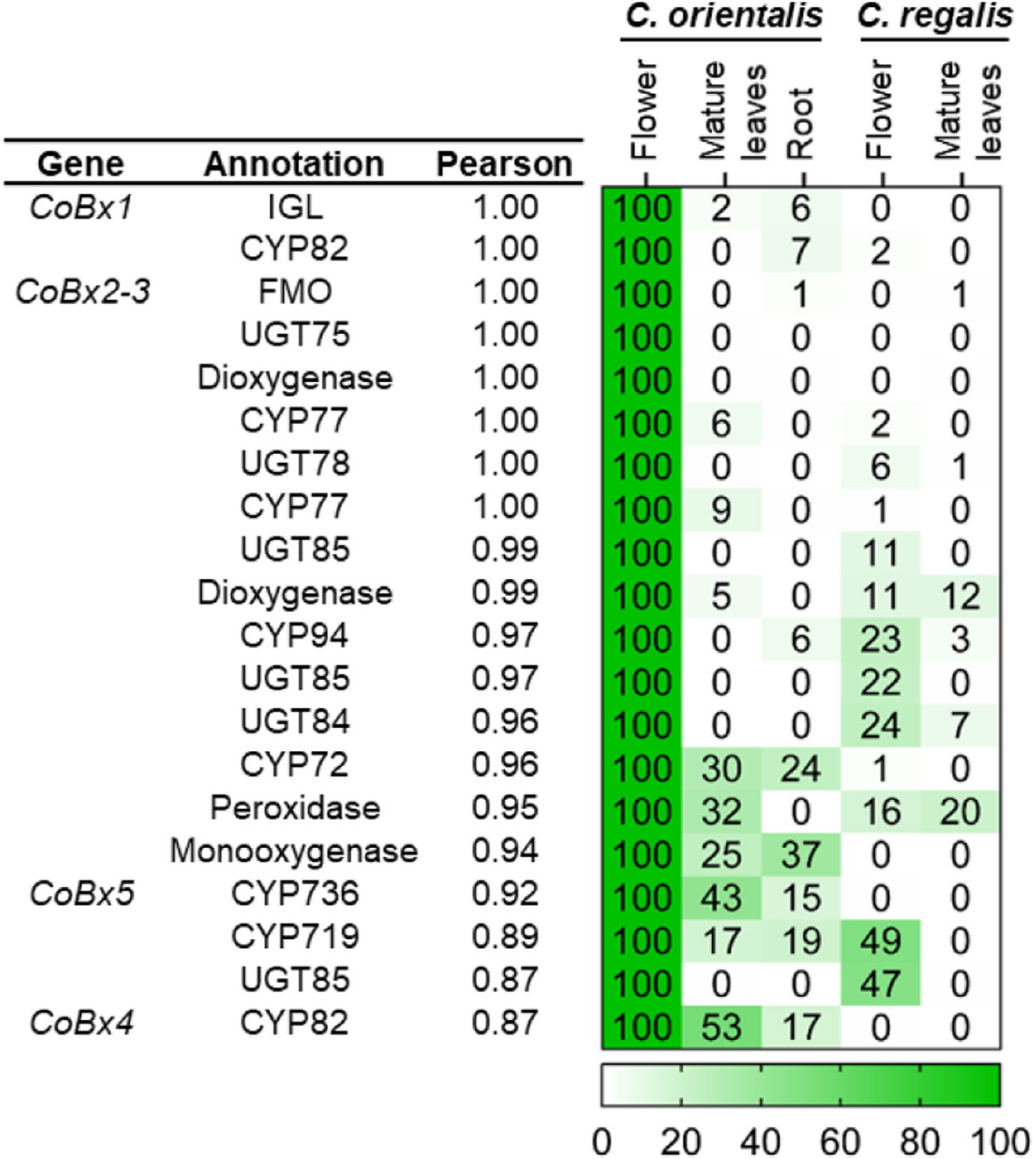
**Relative expression of the 20 *C*. *orientalis* transcripts best correlating with *CoBx1*.** Values from 0 (lowest) to 100 (highest) show the relative expression of each gene among the conditions tested. Genes with RPKM > 20 and Pearson co-expression values with *CoBx1* > 0.7 were selected. The values reported are the average of three biological replicates (independent plants). Enzyme abbreviations: indole-3-glycerol-phosphate lyase (IGL), cytochrome P450 (CYP), flavin-containing monooxygenase (FMO), UDP-glucosyltransferase (UGT).

To identify the remaining genes involved in DIBOA-Glc biosynthesis in *C*. *orientalis*, the transcriptomes of leaves, flowers, and roots of *C*. *orientalis* and leaves and flowers of *C*. *regalis* were sequenced. By generating a *de novo* assembly from all sequenced samples, we identified genes belonging to enzyme classes that could in principle contribute to BXD formation. We further narrowed the pool of candidates by only selecting genes with RPKM values higher than 20 in flowers of *C*. *orientalis*, the tissue with the highest content of DIBOA-Glc. We then compared the gene expression pattern of the previously identified *CoBx1* and the candidate genes by performing a Pearson correlation analysis. Pearson correlation analysis was performed for 152 genes, which respected our selection criteria, over 5 tissue/species samples (Fig. S2). Among the top 20 candidates with Pearson correlation coefficients > 0.7, eight CYP genes, two 2-ODD genes, one FMO gene, one monooxygenase gene, one peroxidase gene, and six UGT genes were identified and considered for further characterization. The majority of genes within the top 20 candidates, prioritized by descending co-expression values, were tested for BX2, BX3, BX4, BX5, and BX8 activities. Genes that showed BXD activity are indicated as *CoBx* (Fig. 3). RPKM values of the selected genes are provided in Fig. S3. Notably, the previously described *CoBx8* had a Pearson correlation coefficient of only 0.36 and, as already reported in the literature, was found to be expressed not only in aerial organs but also in roots (Figs. S2 and 4).

### Flavin-dependent monooxygenases catalyze the formation of 3HI2O from indole in eudicots

**Figure 4:**
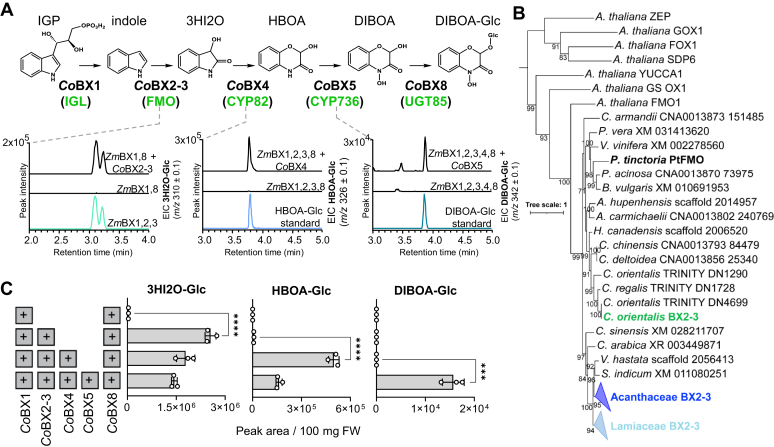
**The identified *Co*BX enzymes allow the biosynthesis of 3HI2O-Glc, HBOA-Glc, and DIBOA-Glc in *N*. *benthamiana***. *A*, *Co*BX2-3, *Co*BX4, and *Co*BX5 were transiently co-expressed with *ZmBx1* + *ZmBx8*, *ZmBx1*, *2*, *3* + *ZmBx8*, and *ZmBx1*, *2*, *3*, *4* + *ZmBx8*, respectively. Leaf material was extracted with methanol and extracts were analyzed using LC-qTOF-MS. Accumulation of BXDs was confirmed with authentic standards for HBOA-Glc and DIBOA-Glc. No authentic standard was available for 3HI2O-Glc, therefore we co-expressed *ZmBx1*, *2*, *3*, + *ZmBx8* in *N*. *benthamiana* to generate this compound. Extracted ion chromatograms (EIC) are shown. *B*, the FMO *Co*BX2-3 independently evolved in the Ranunculaceae, Acanthaceae, and Lamiaceae. Amino acid sequences were aligned with WebPrank and a maximum likelihood tree was inferred using iQTree. Bootstrap values above 70% are displayed. FASTA sequences used for the construction of phylogenetic trees are provided in Supplemental Dataset 2. *C*, *Consolida orientalis* BXD pathway reconstitution. Transient expression of all *C*. *orientalis Bx* genes in *N*. *benthamiana*. 100 ± 5 mg of *N*. *benthamiana* leaf tissue was extracted with methanol. Extracts were analyzed using LC-qTOF-MS. Means and SD are shown (n = 3 biological replicates, independent plants). Unpaired *t* test was used to evaluate the effect of the addition of each *CoBx* gene. 3HI2O-Glc: *p* < 0.0001, t = 26.12, df = 4; HBOA-Glc: *p* < 0.0001, t = 26.60, df = 4; DIBOA-Glc: *p* = 0.0003, t = 12.19, df = 4.

In the Poaceae, the two-step conversion of indole to 3HI2O is catalyzed by two CYP71C enzymes (BX2 and BX3), whereas *L*. *galeobdolon* and *A*. *squarrosa* each possess a dual-function FMO (*Lg*BX2-3 and *As*BX2-3, respectively) that directly oxidizes indole to 3HI2O. Among the top 20 candidate genes tested from *C*. *orientalis*, an FMO (*Co*BX2-3) that showed a Pearson correlation coefficient of 1.0 with *CoBx1*, produced 3HI2O when co-expressed with *Zm*BX1 in *N*. *benthamiana* (Figs. 3 and 4*A*, S5*A*). Unfortunately, we failed to produce active, purified enzyme, precluding more in-depth *in vitro* biochemical experiments (Fig. S6). Phylogenetic analysis showed that the clade containing *Co*BX2-3 was well separated from the clade that contained *Lg*BX2-3 and *As*BX2-3 (Figs. 4*B* and S7). Moreover, *Co*BX2-3 clustered with a previously characterized FMO that is involved in indigo biosynthesis in *Persicaria tinctoria* (*Pt*FMO) (23), a core eudicot belonging to the Poligonaceae family. These phylogenetic analyses strongly suggest that *Co*BX2-3 and *Lg*BX2-3/*As*BX2-3 evolved independently.

### A CYP82 synthesizes HBOA in *C*. *orientalis*

**Figure 5:**
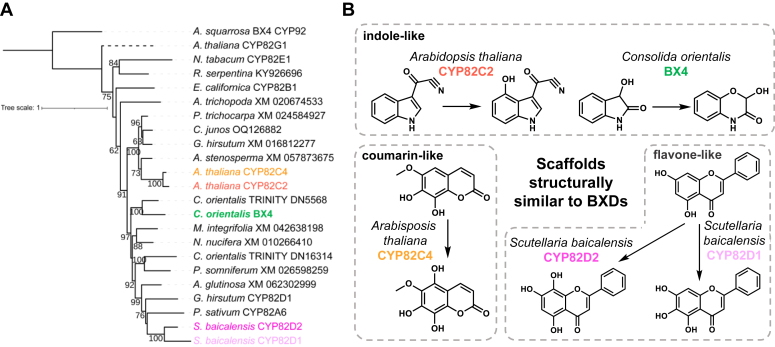
***Co*BX4 is evolutionarily related to CYP82C and CYP82D enzymes that hydroxylate coumarins, flavonoids, or indole-like scaffolds.***A*, amino acid sequences were aligned with WebPrank and a maximum likelihood tree was inferred using iQTree. Bootstrap values above 60% are displayed. FASTA sequences used for the construction of phylogenetic trees are provided in Supplemental Dataset 2. *B*, examples for reactions catalyzed by CYP82C and CYP82D enzymes acting on substrates structurally related to benzoxazinoids.

The oxidative ring expansion of 3HI2O to HBOA is catalyzed by a CYP71 enzyme in maize (*Zm*BX4) and a CYP92 enzyme *A*. *squarrosa* and *L*. *galeobdolon* (*As*BX4/*Lg*BX4). In *C*. *orientalis*, we identified, among the tested candidates, a P450 with a Pearson correlation coefficient of 0.87 (Fig. 3) that, when tested in *N*. *benthamiana* in combination with *Zm*BX1, 2, 3 and *Zm*BX8, produced HBOA-Glc (Figs. 4*A* and S5*B*). The activity of this enzyme, named *Co*BX4, was also confirmed by feeding *CoBx4*-expressing *Saccharomyces cerevisiae* with chemically synthesized 3HI2O and then measuring the accumulation of HBOA by LC-qTOF-MS (Fig. S8). A phylogenetic analysis showed that *Co*BX4 belongs to the CYP82 family (Fig. 5*A*). Since *Co*BX4, *Zm*BX4, and *As*BX4/*Lg*BX4 each belong to different P450 families, parallel evolution of BX4 activity likely occurred in the Poaceae, basal eudicots, and core eudicots.

### A CYP736 converts HBOA to DIBOA

HBOA must undergo *N*-hydroxylation to form DIBOA. In the Poaceae and in *L*. *galeobdolon* this reaction is catalyzed by a CYP71 enzyme (*Zm*BX5 and *Lg*BX5, respectively), whereas *A*. *squarrosa* recruited a CYP81 (*As*BX5) for this reaction. Among the tested candidates, one of the P450 genes with a Pearson correlation coefficient of 0.92 with *CoBx1* showed BX5 activity when tested in *N*. *benthamiana* in combination with *ZmBx1*, *2*, *3*, *4*, and *8* (Figs. 4*A* and S5*C*). Phylogenetic analysis of *Co*BX5 revealed that this enzyme belongs to the CYP736 family and is not related to *A*. *squarrosa As*BX5 (CYP81), *L*. *galeobdolon Lg*BX5 (CYP71D), or maize *Zm*BX5 (CYP71C) (Fig. S9), supporting the hypothesis that the BX5 activity has also evolved independently in all BXD-producing families studied to date.

### None of the tested *Co*UGT candidates showed BXD glycosylation activity

Since the catalytic efficiency of *Co*BX8 is low compared to *Zm*BX8 from maize (Fig. 2*B*), we speculated that this enzyme may not be involved in BXD biosynthesis in *C*. *orientalis*. We thus tested all UGTs in our candidate gene list from *C*. *orientalis* and co-expressed these candidate genes with *ZmBx1*, *2*, *3*, *4*, and *5* in *N*. *benthamiana*. However, none of the UGT enzymes tested showed DIBOA glucosylation activity (Fig. S10). Previously reported *Co*BX8 was the only active UGT although with a Pearson correlation coefficient lower than 0.7 with *CoBx1*.

### Reconstitution of *C*. *orientalis* BXD pathway in *N*. *benthamiana*

To test whether the enzymes identified in this study are able to reconstitute a functional pathway *in planta*, *CoBx2-3*, *CoBx4*, and *CoBx5* were co-expressed in *N*. *benthamiana* with the previously identified *CoBx1* and *CoBx8* genes. LC-qTOF analysis showed that, despite the low activity of *Co*BX8, transient co-expression of *CoBx1*, *2*-*3*, *4*, *5*, and *8* genes in *N*. *benthamiana* resulted in the formation of DIBOA-Glc (Fig. 4*C*).

## Discussion

We report here the elucidation of BXD biosynthesis in a basal family of the eudicots, the Ranunculaceae. Our results show that an FMO and two P450 enzymes are involved in BXD biosynthesis in *C*. *orientalis*. None of these enzymes are related to their counterparts in either the grasses and core eudicots, strongly suggesting that BXD biosynthesis evolved independently in the basal eudicots.

The first and last enzymes of the BXD pathway in *C*. *orientalis*, the IGL *Co*BX1, and the UGT *Co*BX8, respectively, have been described previously. *In vitro* characterization of *Co*BX1 revealed catalytic parameters comparable to those of *Zm*BX1 (9), and we also confirmed the role of this enzyme by heterologous expression in *N*. *benthamiana* (Fig. 2*A*). In contrast, *Co*BX8, previously characterized *in vitro* and by stable transformation in *Arabidopsis thaliana* (11), showed low activity when expressed in *N*. *benthamiana* (Fig. 2*B*). The low efficiency of this enzyme and inconsistent expression pattern compared to other genes involved in BXD metabolism make it questionable whether *Co*BX8 is responsible for glycosylation of BXDs in *C*. *orientalis*. However, screening of four other UGT genes, with organ-specific expression patterns correlating with the expression of BXD genes, did not reveal any other functional candidate. We speculate that a non-specific UGT with an expression pattern that differs from the core BXD genes may be involved in BXD biosynthesis. Alternatively, *C*. *orientalis* may simply have an inefficient *Co*BX8.

Like *A*. *squarrosa* and *L*. *galeobdolon*, *C*. *orientalis* has recruited an FMO capable of performing a double oxidation of indole to 3HI2O (17). However, *Co*BX2-3 has only 54% amino acid sequence similarity to *As*BX2-3 and *Lg*BX2-3 and is found in a different clade of FMOs when subjected to phylogenetic analysis. *Co*BX2-3 instead belongs to a clade that includes an enzyme that catalyzes the oxidation of indole to 3-OH-indole during indigo biosynthesis in *Polygonum tinctorium* (23). The substrates of the other FMOs in the *Co*BX2-3 clade have not been investigated. Nevertheless, it is tempting to speculate that these clade members are characterized by substrate specificity for indole or indole-derived substances, which would facilitate the evolution of BX2-3 activity in *C*. *orientalis*.

The hydroxylation of 3HI2O and the subsequent ring expansion to HBOA is catalyzed by BX4, which in all plants analyzed so far is a P450 monooxygenase. However, while BX4 in the grasses belongs to the CYP71 family (3, 4), Acanthaceae, Apocynaceae, Lamiaceae, and Plantaginaceae have recruited a CYP92 for this reaction (17). Our finding that *C*. *orientalis* possesses a CYP82 capable of catalyzing the BX4 reaction clearly indicates an independent evolution of BX4 activity in monocots, basal eudicots, and core eudicots. It is noteworthy that some previously characterized members of the CYP82C and CYP82D subfamilies, which *Co*BX4 closely resembles, exhibit hydroxylation activities for substrates structurally related to benzoxazinoids. For example, CYP82D1 and CYP82D2 in *Scutellaria baicalensis* function as flavone 6-hydroxylase and flavone 8-hydroxylase, respectively, in the biosynthesis of 4′-deoxyflavones (24). In contrast, CYP82C4 in Arabidopsis hydroxylates the coumarin derivative fraxetin to sideretin (25), and the closely related CYP82C2 is involved in the hydroxylation of 4-hydroxyindole-3-carbonylnitrile (4-OH-ICN) (26) (Fig. 5*A*). While the structural similarity between BXDs and 4-OH-ICN is obvious, coumarin and flavone-like structures also resemble the aglucone or glucosylated form of ring-expanded BXDs such as HBOA. The ability of CYP82C and CYP82D enzymes to hydroxylate the aromatic ring of BXD-like scaffolds may explain a rapid and species-specific evolution of BX4 activity in *C*. *orientalis*.

Like BX4, all known BX5 enzymes belong to the P450 family but were recruited from different subfamilies: CYP736 (*Co*BX5), CYP71C (*Zm*BX5), CYP81 (*As*BX5), and CYP71D (*Lg*BX5) (3, 17). This indicates parallel evolution of *N*-hydroxylation activity (BX5) in the different plant lineages (19). In addition, the identification of *Co*BX5 as CYP736 expands the repertoire of substrates and reactions reported for this P450 family. Previously, CYP736 enzymes were only known to be involved in the biosynthesis of monoterpenes (27) and cyanogenic glucosides (28), and to catalyze the oxidation of biphenyls (29) and sesquiterpenes such as santalene and bergamotene (30).

**Figure 6:**
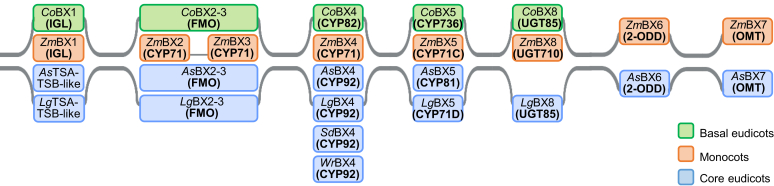
**Independent evolution of the BXD pathway in the angiosperms.** Characterized enzymes and respective enzyme classes are indicated in boxes colored according to the plant lineage in which the enzymes were identified. *As*: *Aphelandra squarrosa*, *Co*: *Consolida orientalis*, *Lg*: *Lamium galeobdolon*, *Sd*: *Scoparia dulcis*, TSB-like: pseudoenzyme involved in indole biosynthesis, *Wr*: *Wrightia religiosa*, *Zm*: *Zea mays*.

The elucidation of the entire BXD pathway in the Ranunculaceae now provides a comprehensive picture of BXD evolution in the angiosperms (Fig. 6). It is now clear that these compounds have evolved independently at least four times: in the monocots, in two lineages of the core eudicots, and the basal eudicots. The BXD pathway is therefore one of the most remarkable examples of the flexibility of plant metabolism. The repeated independent evolution of this pathway also underscores the biological importance of benzoxazinoids as defense and signaling compounds in plants.

## Experimental procedures

### Chemicals

All chemicals were purchased as molecular grade or higher from Sigma Aldrich, Thermo Fisher, or Tokyo Chemical Industry. The same batch of 3HI2O, HBOA, HBOA-Glc, DIBOA, and DIBOA-Glc previously synthesized or purified for (17) was used in this study as reference compounds.

### Plant material and growth

*C*. *orientalis*and*C*. *regalis* plants were grown in a greenhouse at 20 to 24°C during the day and 19 to 22°C during the night, with 45 to 60% humidity on a 16h light/8h dark photoperiod until flowering. *N*. *benthamiana* plants for transient gene expression were grown in a greenhouse at 22°C and 60% humidity on a 16h light/8h dark photoperiod. Plants were grown for 3 weeks before transformation.

### Metabolite extraction

*C*. *orientalis* and *C*. *regalis* tissue was snap-frozen in liquid nitrogen and ground using a mortar and pestle. The resulting powder (100 mg ± 5%) was extracted with 500 μl MeOH, incubated 15 min with vigorous shaking, and then centrifuged in a tabletop centrifuge at maximum speed. Samples were filtered through a PTFE 0.22 μm filter before liquid chromatography/mass spectrometry analysis. *N*. *benthamiana* tissue was sampled using a leaf borer (Ø 1 cm). Four leaf disks (equivalent to ∼ 100 mg) were extracted as reported above.

### Liquid chromatography-quadrupole time-of-flight mass spectrometry (LC-qTOF-MS) analysis

Samples were analyzed as described in Florean *et al*., 2023 (17). In brief, analyses were performed using a Thermo Scientific UltiMate 3000 (Ultra-High Resolution Quadrupole Time-of-flight) system coupled to an Impact II UHR-Q-ToF (Ultra-High Resolution Quadrupole Time-of-Flight) mass spectrometer (Bruker Daltonics). Liquid chromatography was performed using a Phenomenex Kinetex XB-C18 column (100 x 2.1 mm, 2.6 μm; 100 Å) at 35 °C. The mobile phase consisted of H_2_O + 0.1% formic acid (A) and acetonitrile (B) and was run at a flow of 0.3 ml/min. Chromatographic separation was started at 5% B for 1 min, linear gradient from 5% to 50% B in 7 min, 100% B for 2.5 min, 5% B for 2.5 min. A volume of 2 μl was injected for samples and authentic standards (prepared at a concentration of 50–80 μM). Mass spectrometry acquisition was performed in positive or negative electrospray ionization mode depending on the compound of interest as described in (17).

### RNA-sequencing and gene candidate identification

Total RNA from *C*. *orientalis* and *C*. *regalis* tissues was extracted using RNeasy Mini Kit (Quiagen) according to the manufacturer’s instructions. On-column gDNA digestion was performed using RNase-Free DNase Set (Quiagen). RNA quality was assessed using a Nanophotometer N60 (Implen). Three biological replicates of each tissue were prepared. RNA sequencing was performed by Novogene using the company’s standard protocol for library preparation and Illumina RNA sequencing. Approximately 40 million paired-end reads were acquired per sample.

Transcriptome assembly was performed using OmixBox (Biobam). Reads were trimmed and then assempled using the Trinity algorithm implemented in OmixBox with minimum contig length of 400 nt, normalization of max. read coverage of 200, pairs distance of 500, minimum Kmer coverage of 1. Separate transcriptomes for *C*. *orientalis* and *C*. *regalis* were generated. The completeness of the *de novo* transcriptome assemblies was evaluated by calculating the BUSCO scores, and only assemblies with a score of 90% or higher were used. The assembled transcriptomes were annotated using SwissProt 2021 database (blasting parameters: E-Value, 1.0E-3; number of Blast Hits, 20; word size, 3; low complexity filter, on; number of threads, 40; HSP length cutoff, 33). Read mapping was performed on the annotated transcriptomes using CLC Genomics workbench (mapping parameters: mismatch cost, 2; insertion cost, 3; deletion cost, 3; length fraction, 0.8; similarity fraction 0.9; auto-detect paired distances, on; the maximum number of hits for a read, 10). For gene candidate selection, transcripts were first filtered for enzymatic classes of interest and the average RPKM value for each transcript in each condition was calculated. Only transcripts with RPKM values ≥ 20 were kept. Pearson correlation coefficients of the transcripts with *CoBx1* were calculated in Excel. Transcripts were sorted from highest to lowest Pearson correlation coefficient.

### Cloning

Candidate genes were amplified from cDNA prepared from total RNA using SuperScript IV VILO Master Mix (ThermoFisher Scientific) according to the manufacturer’s instruction. Gene amplification was performed using Platinum SuperFi II PCR Master Mix (ThermoFisher Scientific). PCR products were purified using DNA Clean and Concentrator-5 (Zymo). Amplified sequences were inserted in the vectors 3Ω1 (*BsaI-*HF digested) for expression in *N*. *benthamiana*, pOPINF (*HindIII-*HF*/KpnI-*HF digested) for expression in *Escherichia coli*, and pESC-Leu (*NotI-*HF*/PacI-*HF digested) for expression in *S*. *cerevisiae* using In-Phusion HD Cloning (Takara Bio). Constructs were transformed into *E*. *coli* Top10 using the heat shock method and then plated on LB plates with appropriate selection. Single colonies were inoculated in liquid LB with appropriate selection and incubated at 37°C, 250 rpm. Plasmid DNA was purified using Wizard Plus SV Minipreps DNA Purification System (Promega) and the correctness of the insert was checked through Sanger sequencing. Primers used in this study are given in Table S2.

### *Agrobacterium tumefaciens*-mediated transient transformation of *N*. *benthamiana*

Electrocompetent *A*. *tumefaciens* GV3101 (Goldbio) cells were thawed on ice and combined with 100 ng of sequence-verified plasmid. Following a 30-min incubation on ice, the cell-plasmid mixture was transferred to an electroporation cuvette (1 cm path length) and electroporated using a BioRad Micropulser. Transformed cells were then recovered in 1 ml of LB medium and incubated for 3 h at 28°C, 200 rpm. Cells were plated onto LB-agar plates with appropriate selection and incubated at 28°C for 48 h. Single colonies were inoculated in liquid LB with selection, followed by overnight incubation at 28°C with shaking at 200 rpm. For transient transformation of *N*. *benthamiana*, overnight cultures were centrifuged at 4000 rpm for 10 min at 14°C. The pellet was resuspended in infiltration medium (10 mM MES, 10 mM MgCl₂, 100 μM acetosyringone, pH 5.7) to achieve an OD600 of 0.8 to 0.9 and incubated for 1.5 to 2.5 h at 28°C with shaking at 200 rpm. Desired co-infiltration mixtures were prepared by combining equal volumes of the infiltration solutions. These mixtures were introduced into the abaxial side of 3-week-old *N*. *benthamiana* leaves using a needle-less 1 ml syringe. After infiltration, plants were transferred to a growth chamber under standard light conditions. Infiltrated leaves were harvested 5 days post-infiltration. In all transformations, a plasmid encoding the silencing suppressor protein p19 was co-infiltrated to enhance expression.

### Heterologous expression of genes in *S*. *cerevisiae*

*S*. *cerevisiae* WAT11 cells were made competent using the lithium acetate method (31). Briefly, *S*. *cerevisiae* WAT11 was streaked onto YPAD agar plates to isolate single colonies. A 10 ml YPAD seed culture was inoculated with a single colony and grown overnight at 30°C with shaking at 180 rpm. The overnight culture was subsequently diluted into 40 ml of fresh YPAD and incubated for an additional 4 to 5 h. Cells were harvested by centrifugation (3000 x g, 4°C, 5 min) and washed with ddH₂O. Aliquots of the resuspended cells were pelleted by centrifugation, and then resuspended in a transformation mixture containing 240 μl PEG 3350 (50% w/v), 36 μl lithium acetate (1 M), 100 μg salmon testis DNA, and 1 μg plasmid DNA. The transformation mixture was incubated at 42°C for 50 min, followed by centrifugation. The pellet was resuspended in 300 μl ddH₂O and plated on SD-Leu agar plates supplemented with 2% glucose. Plates were incubated at 30°C for 48 to 72 h. For confirmation of transformation, 2 μl of single colonies lysed in 20 μl of 20 mM NaOH and boiled at 100°C for 10 min were used in a 15 μl PCR reaction.

### Enzymatic activity screening in *S*. *cerevisiae*

Positive *S*. *cerevisiae* WAT11 colonies were inoculated in 30 ml SD-Leu medium (+2% glucose) and incubated at 30°C, 180 rpm overnight. A volume of cell culture corresponding to OD600 = 1 was added to 100 ml SD-Leu medium (+2% glucose) and incubated for 30 to 35 h at 30°C, 180 rpm. Cells were pelleted and resuspended in 100 ml SD-Leu (+1.8% galactose and 0.2% glucose). 300 μl aliquots of cell culture were transferred to Eppendorf tubes, supplemented with 1 mM substrate, and incubated for 24 h at 30°C, 180 rpm. The samples were worked up by the addition of 300 μl of MeOH followed by 30 min sonication in a sonic bath. Cell debris was then pelleted by centrifugation and the samples were filtered through a PTFE 0.22 μm filter for LC-qToF-MS analysis.

### Heterologous expression of genes in *E*. *coli* and *in vitro* assays

*E*. *coli* DE3 (ThermoFisher Scientific) was transformed with sequence-verified plasmids using the heat-shock method, plated on selective LB-agar, and grown overnight at 37°C. Single colonies were inoculated in a selective LB medium and cultured overnight at 37°C, 250 rpm. A seed culture (100 μl) was used to inoculate 100 ml of 2x YT medium, grown to OD600 = 0.4 to 0.6, and incubated at 18°C for 20 min before induction with 500 μM IPTG. Induced cultures were incubated overnight at 18°C, 250 rpm. Cells were harvested by centrifugation (4000 x g, 4°C, 10 min) and resuspended in A1 buffer (50 mM TRIS-HCl, 50 mM glycine, 5% glycerol, 0.5 M NaCl, 20 mM imidazole, pH 8) supplemented with lysozyme and protease inhibitors. Cells were disrupted by sonication (2 min, 2 s on/3 s off) on ice, and the lysate was clarified by centrifugation (32,000 x g, 4°C, 20 min). His-tagged proteins were purified using Ni-NTA agarose beads (Takara) and eluted with buffer A1 additioned of 500 mM imidazole. Elution buffer was exchanged for storage buffer (20 mM HEPES, 150 mM NaCl, pH 7.5) using Amicon 30 kDa concentrator columns (Merck Millipore), and proteins were stored at −20°C. Protein concentration was determined spectrophotometrically using an Implen spectrophotometer. *Co*BX2/3 *in vitro* assays were performed in KPO_4_ buffer (25 mM, pH 7.5) containing 1 mM DTT, 1 mM NADPH, 500 μM FAD, 1 mM substrate, and 2 μg of protein. Reactions were incubated 12 h at 30°C with gentle shaking.

### SDS-page and Western blot

His-purified proteins were analyzed by SDS-page using Novex 12%, Tris-Glycine Plus WedgeWell gels (Invitrogen) according to manufacturer’s instructions. For Western Blot analysis, gels were transferred on a Power Blotter Select Transfer Stack PVDF Mini Size membrane (Invitrogen) using Power Blotter XL transfer station (Invitrogen). Blotted membranes were blocked in TBS + 1 ml/L Tween buffer (TBST) + 5% (w/v) skimmed milk for 1 h at room temperature. The blocking solution was removed and membrane incubated in TBST + 3% (w/v) skimmed milk and anti-Histidine antibody coupled with Horseradish peroxidase (BioRad) as per manufacturer’s instructions. Western blots were imaged with Clarity Western ECL Substrate (BioRad) as per the manufacturer’s instructions.

### Statistical analysis

Statistical analyses were performed in GraphPad Prism version 10.0.3. Details (statistical test and *p* values) are given in the respective figure legends.

## Data availability

Transcriptome raw reads were deposited in the NCBI Sequence Read Archive (SRA) under the BioProject accession PRJNA1153027. Characterized genes were deposited in NCBI GenBank with the accession numbers reported in Table S1. All other data is included in the manuscript or in the Supplementary information. This article contains supporting information.

## Supporting information

This article contains supporting information.

## Conflict of interest

The authors declare that they have no conflicts of interest with the contents of this article.
