# Supplementary material for: Independent evolution of plant natural products: Formation of benzoxazinoids in Consolida orientalis (Ranunculaceae)
Source: J Biol Chem. 2024 Nov 26;301(1):108019. doi: 10.1016/j.jbc.2024.108019 (PMC11742589; doi:10.1016/j.jbc.2024.108019)
Supplement: Supplemental Figures S1–S10 [file mmc1.pptx]

## Slide 1
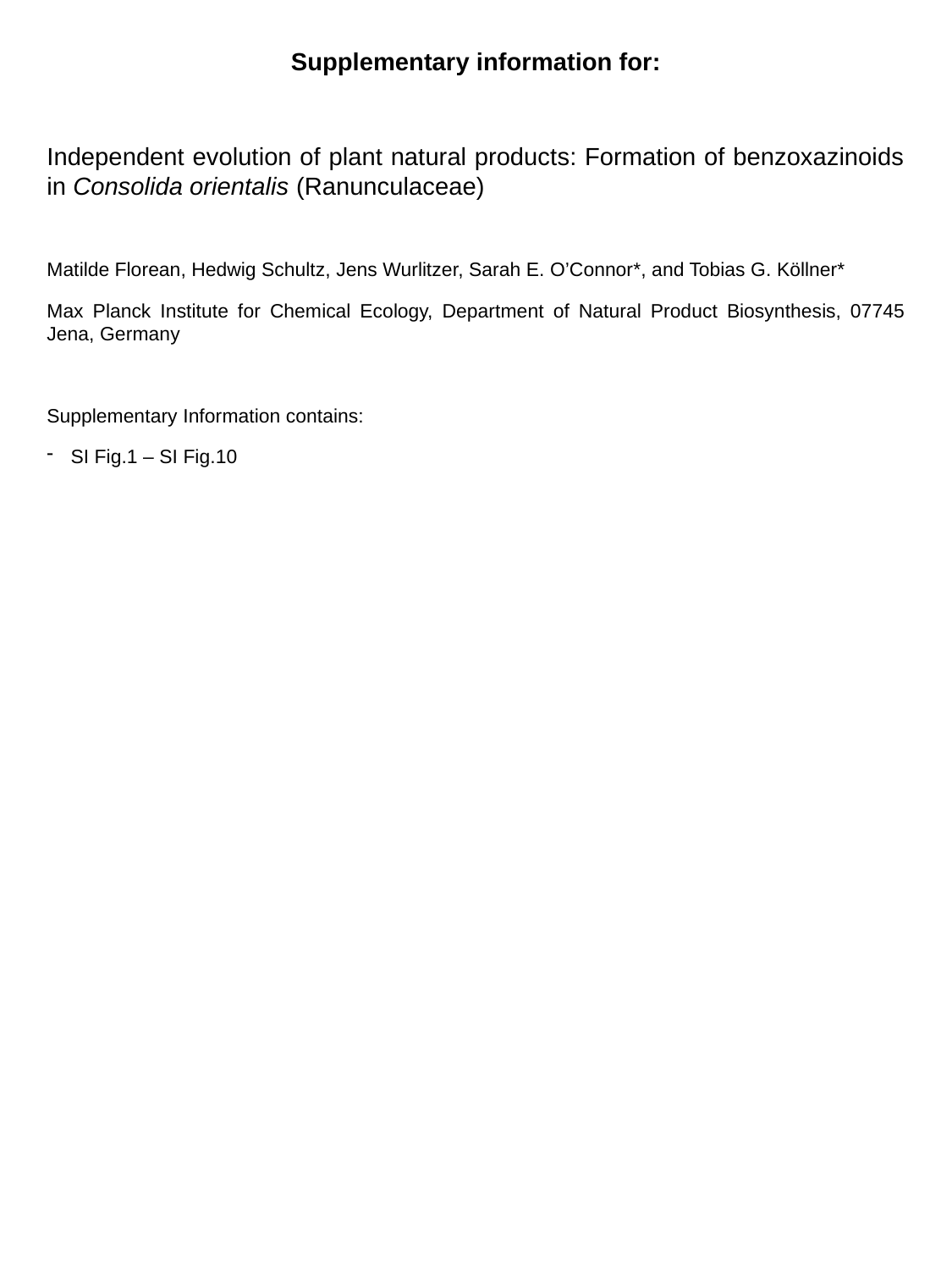

Supplementary information for:
Independent evolution of plant natural products: Formation of benzoxazinoids in Consolida orientalis (Ranunculaceae)
Matilde Florean, Hedwig Schultz, Jens Wurlitzer, Sarah E. O’Connor*, and Tobias G. Köllner*
Max Planck Institute for Chemical Ecology, Department of Natural Product Biosynthesis, 07745 Jena, Germany
Supplementary Information contains:
SI Fig.1 – SI Fig.10

## Slide 2
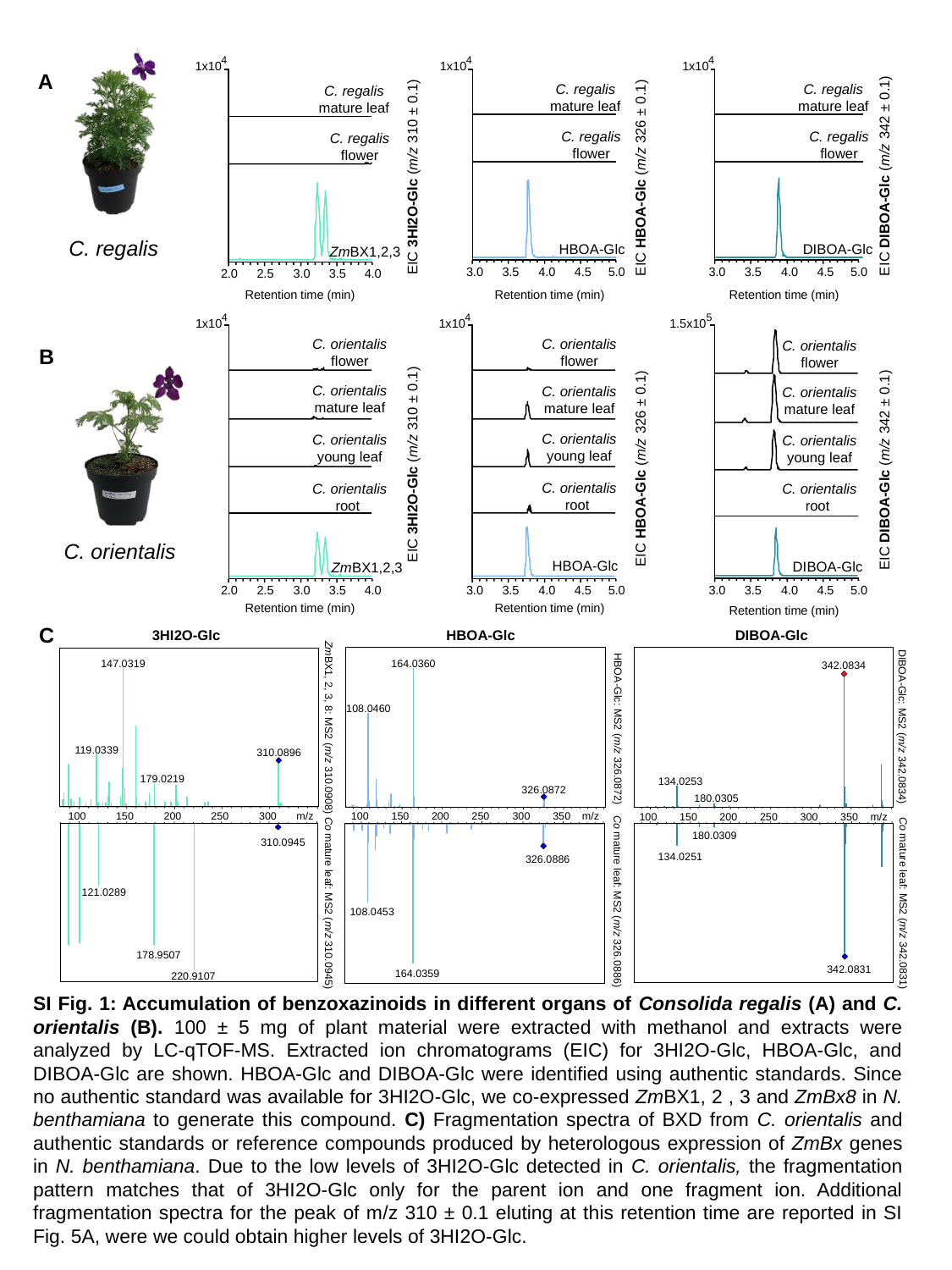

4
1x10
4
1x10
4
1x10
C. regalis
mature leaf
C. regalis
mature leaf
C. regalis
mature leaf
C. regalis
flower
C. regalis
flower
C. regalis
flower
EIC DIBOA-Glc (m/z 342 ± 0.1)
EIC 3HI2O-Glc (m/z 310 ± 0.1)
EIC HBOA-Glc (m/z 326 ± 0.1)
HBOA-Glc
DIBOA-Glc
ZmBX1,2,3
3.0
3.5
4.0
4.5
5.0
3.0
3.5
4.0
4.5
5.0
2.0
2.5
3.0
3.5
4.0
Retention time (min)
Retention time (min)
Retention time (min)
A
C. regalis
4
1x10
4
1x10
5
1.5x10
C. orientalis
flower
C. orientalis
mature leaf
C. orientalis
young leaf
C. orientalis
root
ZmBX1,2,3
2.0
2.5
3.0
3.5
4.0
Retention time (min)
C. orientalis
flower
C. orientalis
flower
C. orientalis
mature leaf
C. orientalis
mature leaf
C. orientalis
young leaf
C. orientalis
young leaf
EIC 3HI2O-Glc (m/z 310 ± 0.1)
EIC HBOA-Glc (m/z 326 ± 0.1)
EIC DIBOA-Glc (m/z 342 ± 0.1)
C. orientalis
root
C. orientalis
root
HBOA-Glc
DIBOA-Glc
3.0
3.5
4.0
4.5
5.0
3.0
3.5
4.0
4.5
5.0
Retention time (min)
Retention time (min)
B
C. orientalis
C
3HI2O-Glc
HBOA-Glc
DIBOA-Glc
147.0319
ZmBX1, 2, 3, 8: MS2 (m/z 310.0908)
119.0339
310.0896
179.0219
100
150
200
250
300
m/z
164.0360
108.0460
HBOA-Glc: MS2 (m/z 326.0872)
326.0872
100
150
200
250
300
350
m/z
342.0834
DIBOA-Glc: MS2 (m/z 342.0834)
134.0253
180.0305
100
150
200
250
300
350
m/z
326.0886
Co mature leaf: MS2 (m/z 326.0886)
108.0453
164.0359
310.0945
121.0289
Co mature leaf: MS2 (m/z 310.0945)
178.9507
220.9107
180.0309
134.0251
Co mature leaf: MS2 (m/z 342.0831)
342.0831
SI Fig. 1: Accumulation of benzoxazinoids in different organs of Consolida regalis (A) and C. orientalis (B). 100 ± 5 mg of plant material were extracted with methanol and extracts were analyzed by LC-qTOF-MS. Extracted ion chromatograms (EIC) for 3HI2O-Glc, HBOA-Glc, and DIBOA-Glc are shown. HBOA-Glc and DIBOA-Glc were identified using authentic standards. Since no authentic standard was available for 3HI2O-Glc, we co-expressed ZmBX1, 2 , 3 and ZmBx8 in N. benthamiana to generate this compound. C) Fragmentation spectra of BXD from C. orientalis and authentic standards or reference compounds produced by heterologous expression of ZmBx genes in N. benthamiana. Due to the low levels of 3HI2O-Glc detected in C. orientalis, the fragmentation pattern matches that of 3HI2O-Glc only for the parent ion and one fragment ion. Additional fragmentation spectra for the peak of m/z 310 ± 0.1 eluting at this retention time are reported in SI Fig. 5A, were we could obtain higher levels of 3HI2O-Glc.

## Slide 3
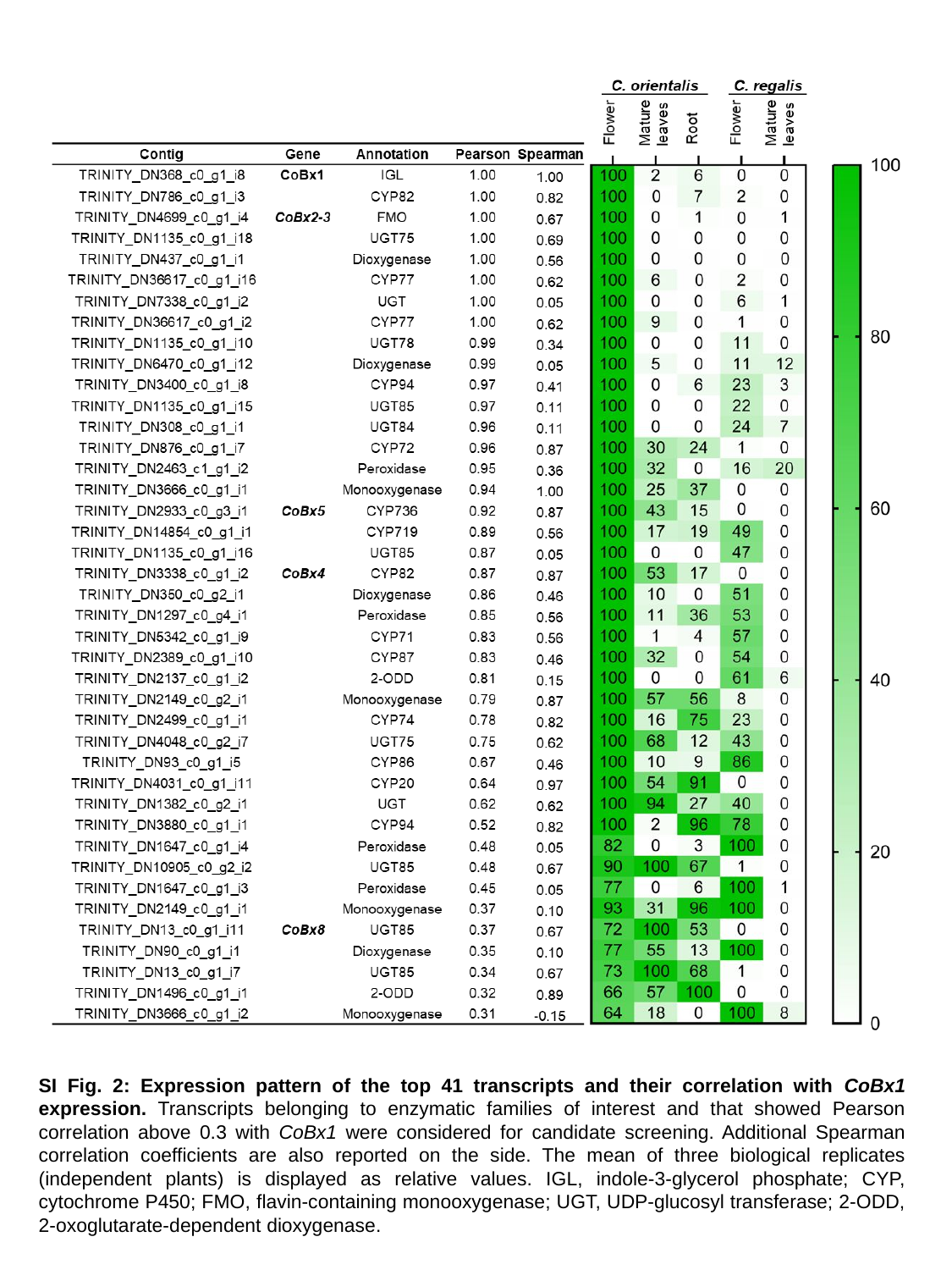

SI Fig. 2: Expression pattern of the top 41 transcripts and their correlation with CoBx1 expression. Transcripts belonging to enzymatic families of interest and that showed Pearson correlation above 0.3 with CoBx1 were considered for candidate screening. Additional Spearman correlation coefficients are also reported on the side. The mean of three biological replicates (independent plants) is displayed as relative values. IGL, indole-3-glycerol phosphate; CYP, cytochrome P450; FMO, flavin-containing monooxygenase; UGT, UDP-glucosyl transferase; 2-ODD, 2-oxoglutarate-dependent dioxygenase.

## Slide 4
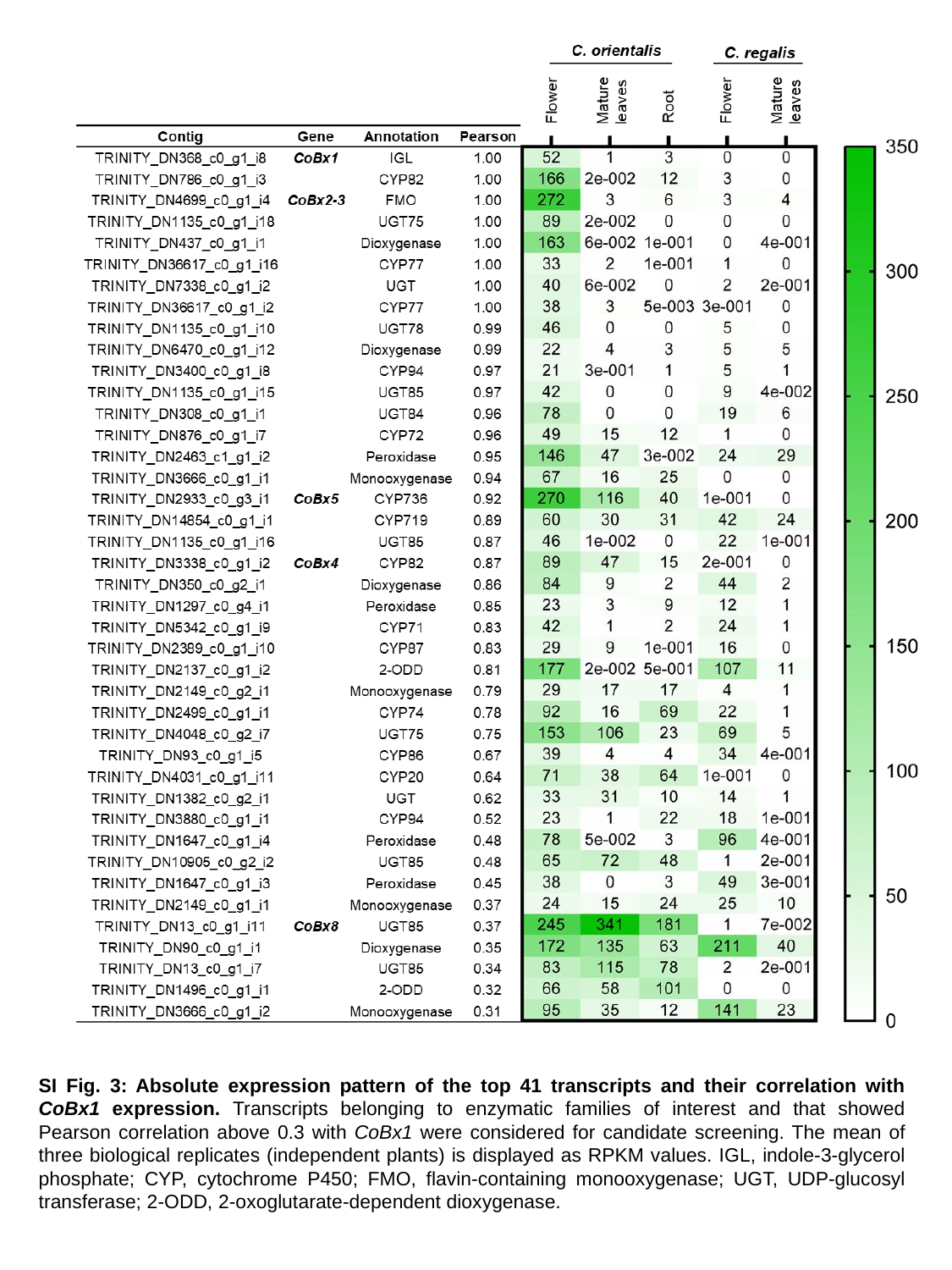

SI Fig. 3: Absolute expression pattern of the top 41 transcripts and their correlation with CoBx1 expression. Transcripts belonging to enzymatic families of interest and that showed Pearson correlation above 0.3 with CoBx1 were considered for candidate screening. The mean of three biological replicates (independent plants) is displayed as RPKM values. IGL, indole-3-glycerol phosphate; CYP, cytochrome P450; FMO, flavin-containing monooxygenase; UGT, UDP-glucosyl transferase; 2-ODD, 2-oxoglutarate-dependent dioxygenase.

## Slide 5
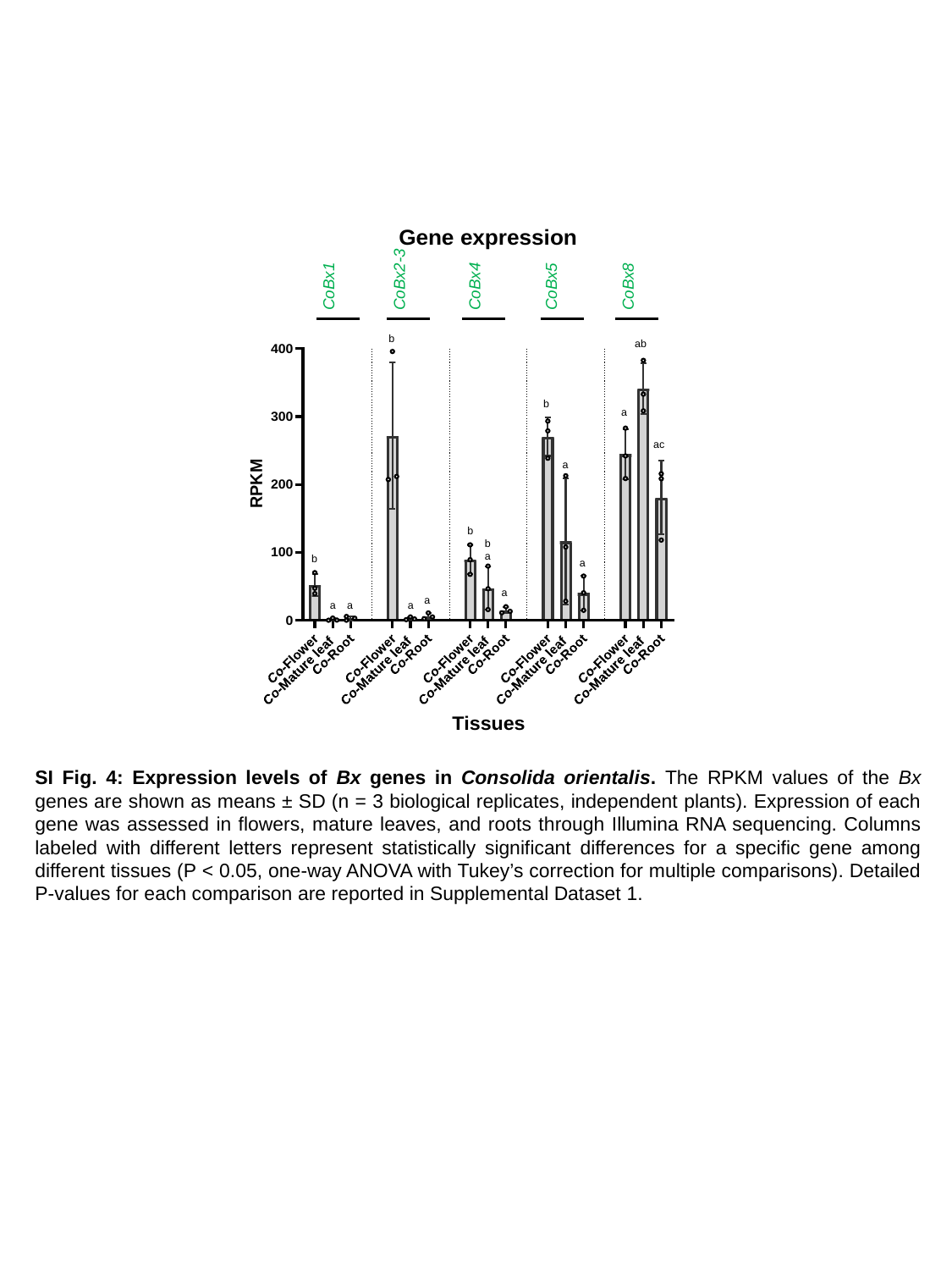

CoBx1
CoBx2-3
CoBx4
CoBx5
CoBx8
b
ab
b
a
ac
a
b
b
a
b
a
a
a
a
a
a
SI Fig. 4: Expression levels of Bx genes in Consolida orientalis. The RPKM values of the Bx genes are shown as means ± SD (n = 3 biological replicates, independent plants). Expression of each gene was assessed in flowers, mature leaves, and roots through Illumina RNA sequencing. Columns labeled with different letters represent statistically significant differences for a specific gene among different tissues (P < 0.05, one-way ANOVA with Tukey’s correction for multiple comparisons). Detailed P-values for each comparison are reported in Supplemental Dataset 1.

## Slide 6
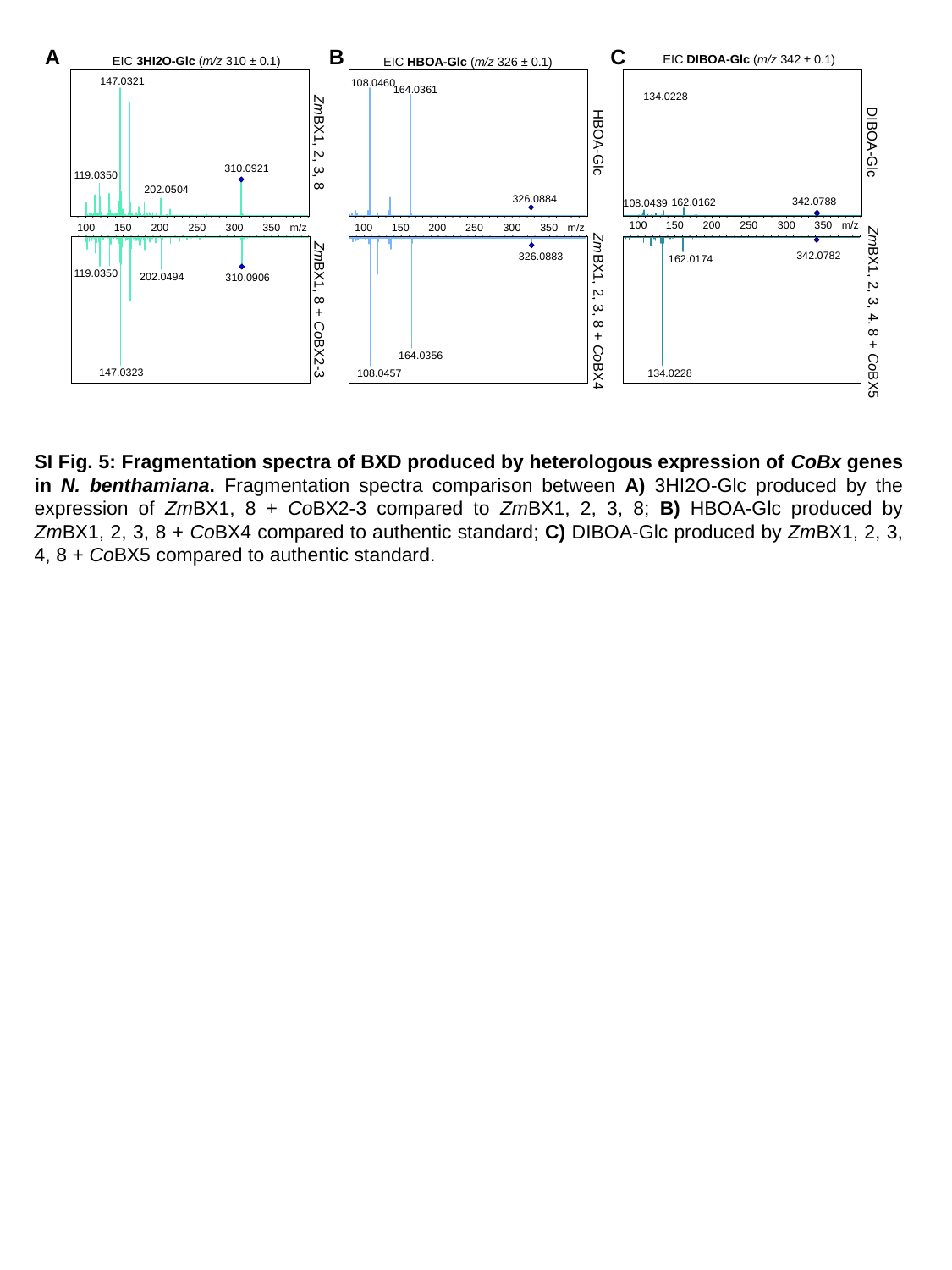

A
B
C
EIC DIBOA-Glc (m/z 342 ± 0.1)
EIC 3HI2O-Glc (m/z 310 ± 0.1)
EIC HBOA-Glc (m/z 326 ± 0.1)
147.0321
310.0921
119.0350
202.0504
108.0460
164.0361
326.0884
100
150
200
250
300
350
m/z
134.0228
342.0788
162.0162
108.0439
100
150
200
250
300
350
m/z
ZmBX1, 2, 3, 8
HBOA-Glc
DIBOA-Glc
147.0323
310.0906
202.0494
119.0350
100
150
200
250
300
350
m/z
342.0782
162.0174
134.0228
326.0883
164.0356
108.0457
ZmBX1, 8 + CoBX2-3
ZmBX1, 2, 3, 8 + CoBX4
ZmBX1, 2, 3, 4, 8 + CoBX5
SI Fig. 5: Fragmentation spectra of BXD produced by heterologous expression of CoBx genes in N. benthamiana. Fragmentation spectra comparison between A) 3HI2O-Glc produced by the expression of ZmBX1, 8 + CoBX2-3 compared to ZmBX1, 2, 3, 8; B) HBOA-Glc produced by ZmBX1, 2, 3, 8 + CoBX4 compared to authentic standard; C) DIBOA-Glc produced by ZmBX1, 2, 3, 4, 8 + CoBX5 compared to authentic standard.

## Slide 7
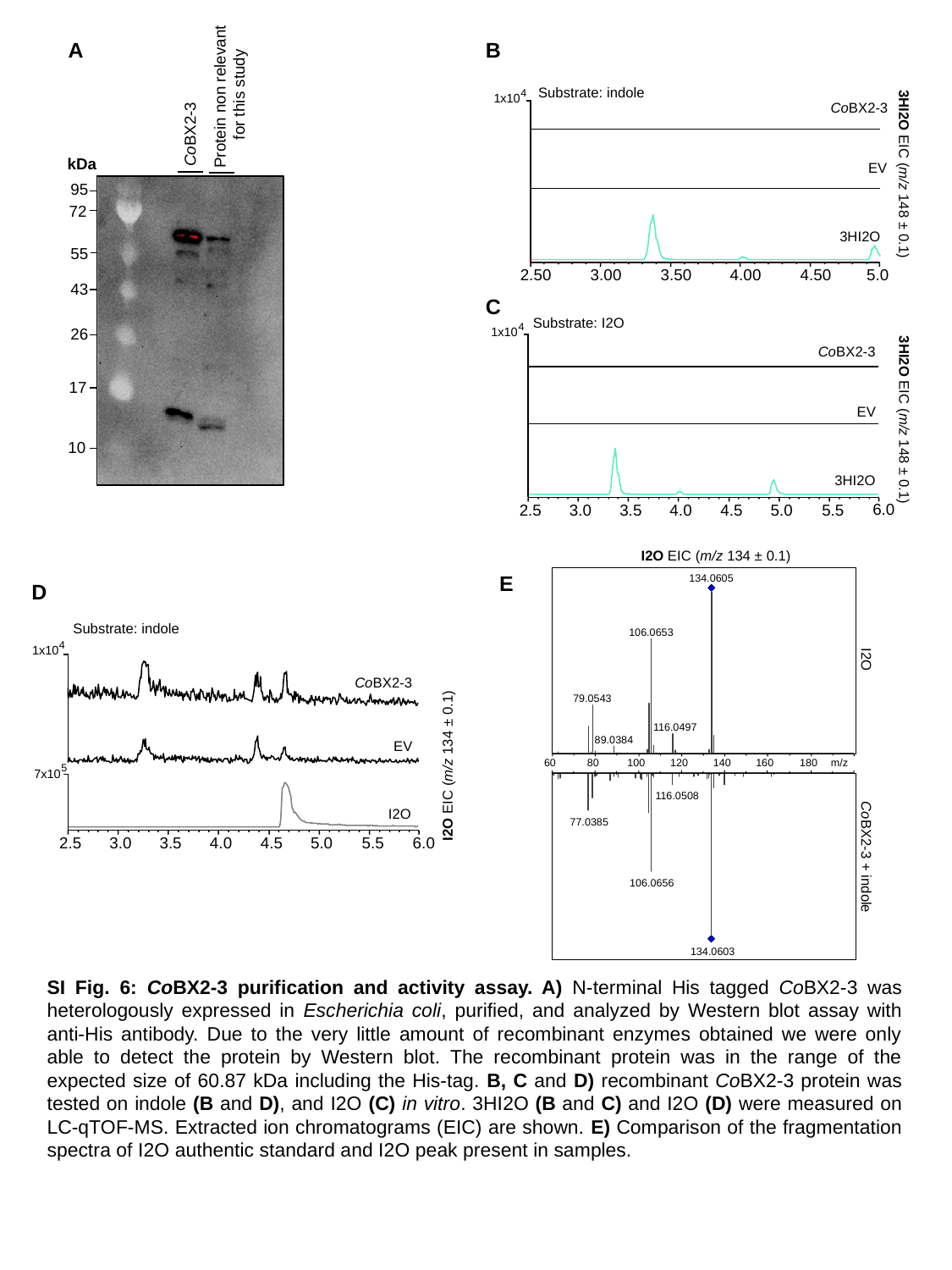

A
B
Protein non relevant
for this study
Substrate: indole
4
1x10
CoBX2-3
EV
3HI2O
2.50
3.00
3.50
4.00
4.50
5.0
CoBX2-3
kDa
95
72
55
43
26
17
3HI2O EIC (m/z 148 ± 0.1)
C
Substrate: I2O
4
1x10
CoBX2-3
EV
3HI2O EIC (m/z 148 ± 0.1)
3HI2O
6.0
5.5
2.5
3.0
3.5
4.0
4.5
5.0
10
I2O EIC (m/z 134 ± 0.1)
E
134.0605
D
CoBX2-3
I2O EIC (m/z 134 ± 0.1)
EV
I2O
6.0
5.5
2.5
3.0
3.5
4.0
4.5
5.0
Substrate: indole
4
1x10
5
7x10
106.0653
I2O
79.0543
116.0497
89.0384
60
80
100
120
140
160
180
m/z
116.0508
77.0385
CoBX2-3 + indole
106.0656
134.0603
SI Fig. 6: CoBX2-3 purification and activity assay. A) N-terminal His tagged CoBX2-3 was heterologously expressed in Escherichia coli, purified, and analyzed by Western blot assay with anti-His antibody. Due to the very little amount of recombinant enzymes obtained we were only able to detect the protein by Western blot. The recombinant protein was in the range of the expected size of 60.87 kDa including the His-tag. B, C and D) recombinant CoBX2-3 protein was tested on indole (B and D), and I2O (C) in vitro. 3HI2O (B and C) and I2O (D) were measured on LC-qTOF-MS. Extracted ion chromatograms (EIC) are shown. E) Comparison of the fragmentation spectra of I2O authentic standard and I2O peak present in samples.

## Slide 8
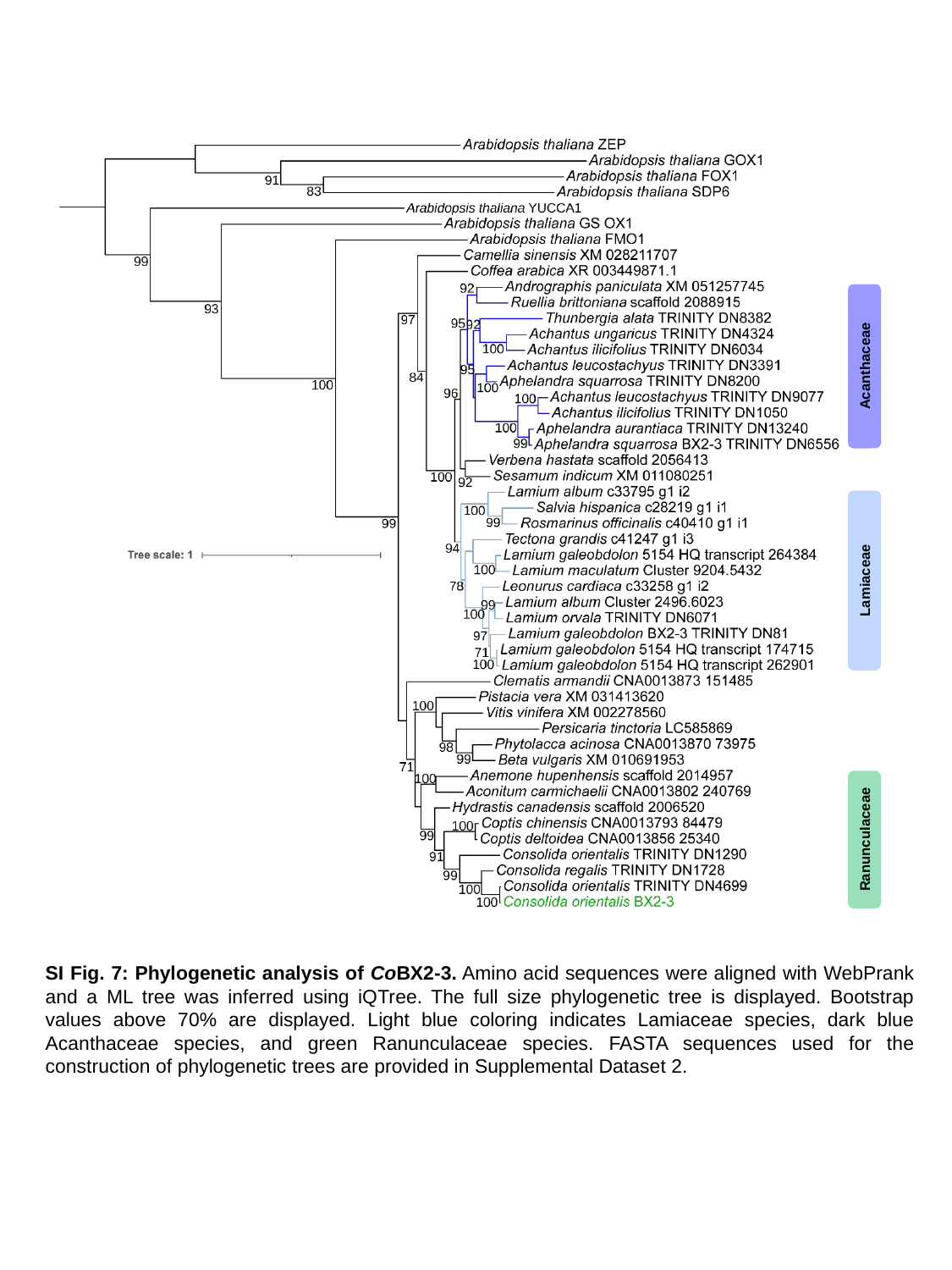

Acanthaceae
Lamiaceae
Ranunculaceae
SI Fig. 7: Phylogenetic analysis of CoBX2-3. Amino acid sequences were aligned with WebPrank and a ML tree was inferred using iQTree. The full size phylogenetic tree is displayed. Bootstrap values above 70% are displayed. Light blue coloring indicates Lamiaceae species, dark blue Acanthaceae species, and green Ranunculaceae species. FASTA sequences used for the construction of phylogenetic trees are provided in Supplemental Dataset 2.

## Slide 9
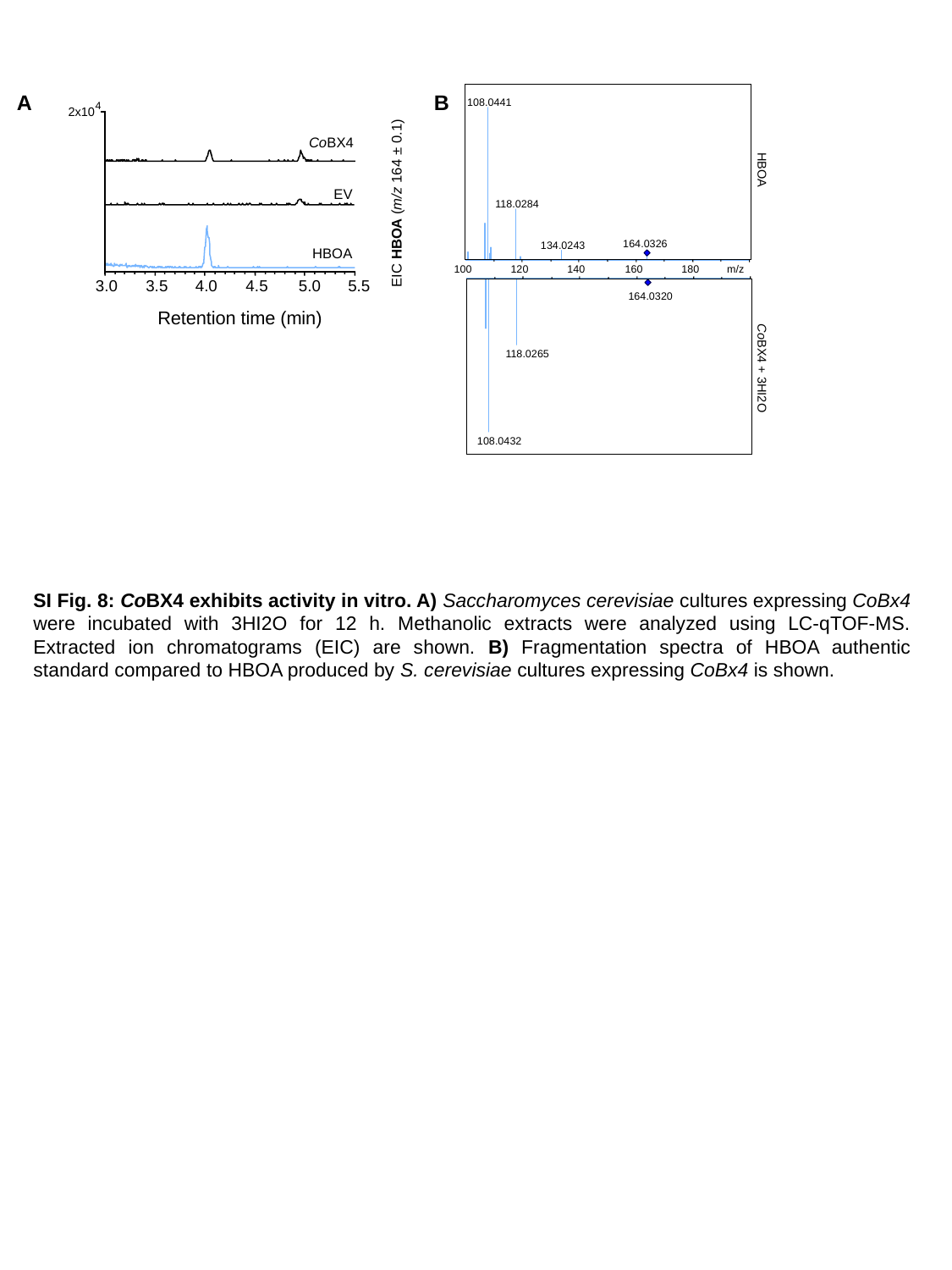

CoBX4
EV
HBOA
3.0
3.5
4.0
4.5
5.0
5.5
EIC HBOA (m/z 164 ± 0.1)
A
B
4
2x10
108.0441
HBOA
118.0284
164.0326
134.0243
100
120
140
160
180
m/z
164.0320
Retention time (min)
118.0265
CoBX4 + 3HI2O
108.0432
SI Fig. 8: CoBX4 exhibits activity in vitro. A) Saccharomyces cerevisiae cultures expressing CoBx4 were incubated with 3HI2O for 12 h. Methanolic extracts were analyzed using LC-qTOF-MS. Extracted ion chromatograms (EIC) are shown. B) Fragmentation spectra of HBOA authentic standard compared to HBOA produced by S. cerevisiae cultures expressing CoBx4 is shown.

## Slide 10
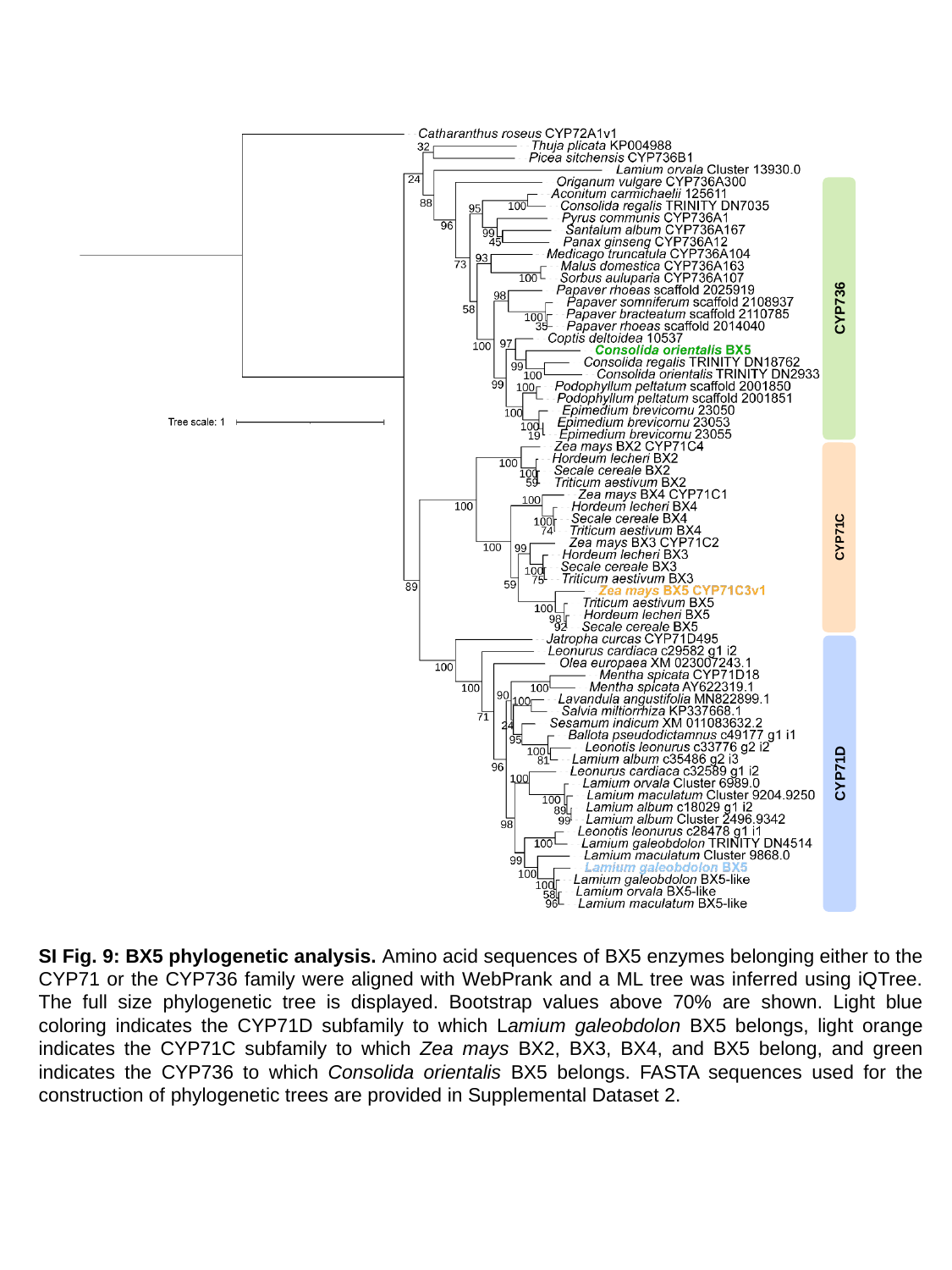

CYP736
CYP71C
CYP71D
SI Fig. 9: BX5 phylogenetic analysis. Amino acid sequences of BX5 enzymes belonging either to the CYP71 or the CYP736 family were aligned with WebPrank and a ML tree was inferred using iQTree. The full size phylogenetic tree is displayed. Bootstrap values above 70% are shown. Light blue coloring indicates the CYP71D subfamily to which Lamium galeobdolon BX5 belongs, light orange indicates the CYP71C subfamily to which Zea mays BX2, BX3, BX4, and BX5 belong, and green indicates the CYP736 to which Consolida orientalis BX5 belongs. FASTA sequences used for the construction of phylogenetic trees are provided in Supplemental Dataset 2.

## Slide 11
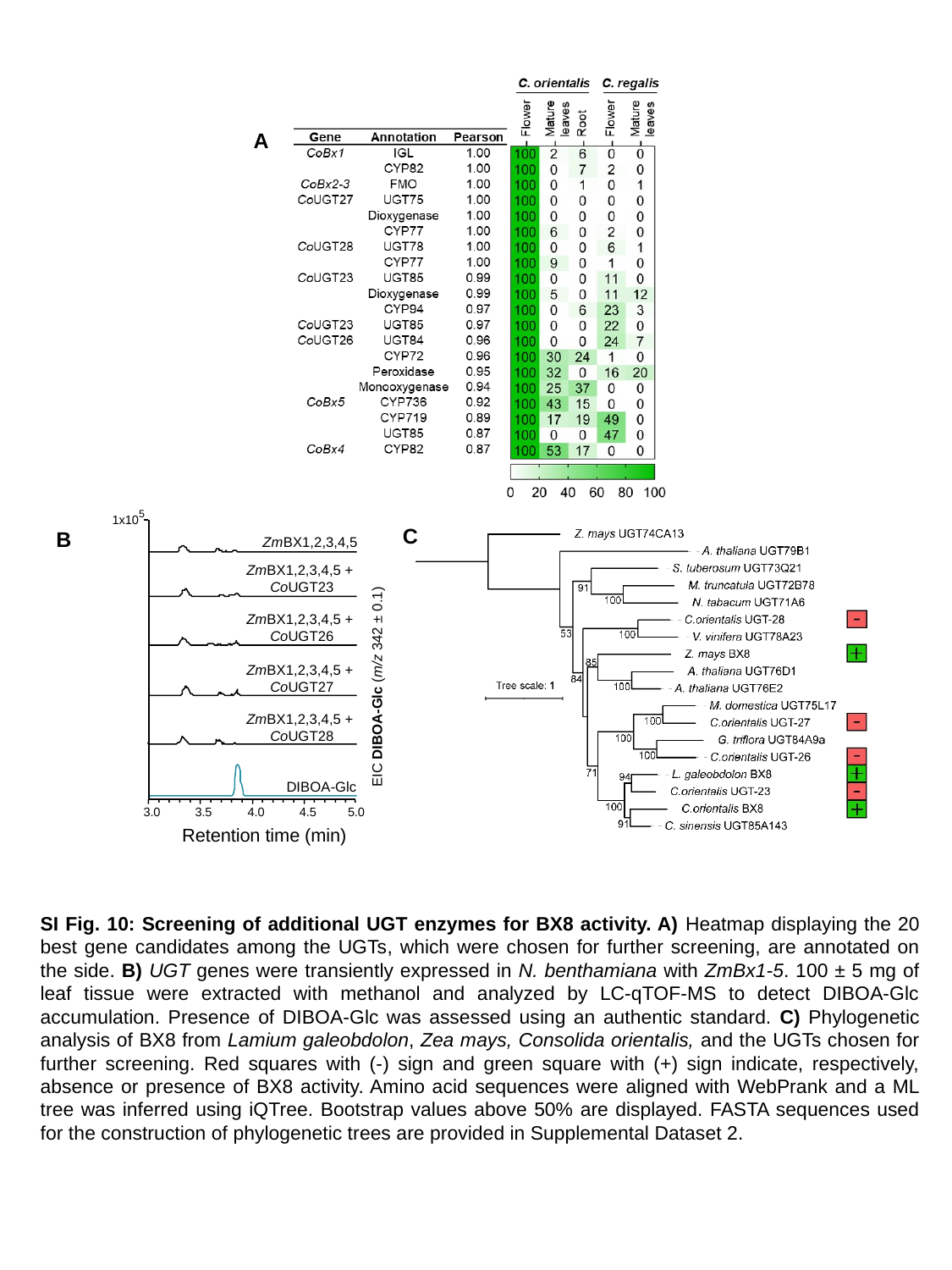

A
5
1x10
C
B
ZmBX1,2,3,4,5
ZmBX1,2,3,4,5 +
CoUGT23
ZmBX1,2,3,4,5 +
CoUGT26
ZmBX1,2,3,4,5 +
CoUGT27
ZmBX1,2,3,4,5 +
CoUGT28
DIBOA-Glc
3.0
3.5
4.0
4.5
5.0
EIC DIBOA-Glc (m/z 342 ± 0.1)
Retention time (min)
SI Fig. 10: Screening of additional UGT enzymes for BX8 activity. A) Heatmap displaying the 20 best gene candidates among the UGTs, which were chosen for further screening, are annotated on the side. B) UGT genes were transiently expressed in N. benthamiana with ZmBx1-5. 100 ± 5 mg of leaf tissue were extracted with methanol and analyzed by LC-qTOF-MS to detect DIBOA-Glc accumulation. Presence of DIBOA-Glc was assessed using an authentic standard. C) Phylogenetic analysis of BX8 from Lamium galeobdolon, Zea mays, Consolida orientalis, and the UGTs chosen for further screening. Red squares with (-) sign and green square with (+) sign indicate, respectively, absence or presence of BX8 activity. Amino acid sequences were aligned with WebPrank and a ML tree was inferred using iQTree. Bootstrap values above 50% are displayed. FASTA sequences used for the construction of phylogenetic trees are provided in Supplemental Dataset 2.
