# Supplementary material for: Independent evolution of plant natural products: Formation of benzoxazinoids in Consolida orientalis (Ranunculaceae)
Source: J Biol Chem. 2024 Nov 26;301(1):108019. doi: 10.1016/j.jbc.2024.108019 (PMC11742589; doi:10.1016/j.jbc.2024.108019)
Supplement: Supplemental Dataset 2 [file mmc5.docx]

**Additional datasets for:**

**Fig. 4 and SI Fig. 7, FMO phylogeny:**

>Arabidopsis thaliana_FMO1_AT1G19250

MASNYDKLTSSRVAIIGAGVSGLAAAKNLVHHNPTVFEASDSVGGVWRSCTYETTKLQSARVDYEFSDFPWPNNRDDTTFPPYLEILDYLESYAKHFDLLKFMKFGSKVIEVRFIGDGETPQMVDLGAYGNLLPGKPVWEVAVQIGDSGDIQWHAFEFVVVCTGKYGDVPRIPAFPAKKGPEMFQGKVMHSMDYCKLEKEEASTLLSGKKVAVIGFKKSAIDLALESALANQGEGGKACTMVVRTTHWGIPHYWVWGLPFFLFYSSRASQFLHDRPNQSFLRTLFCLLFSLLRAVVSKFIESYVLWKLPLEKYGLKPNHSFEEDYASCQMAIIPENFFEEADKGMIRFKKSSKWWFYEEGIVFEDGTTLEADVVILATGYDGKKKLKAIVPEPFRTWLEFPSGVMPLYRGTIHPLIPNMGFVGYVQSSSNLHTSELRSMWLSRLVDEKFRLPSKEKMLDQFLKEMEVTRNSSRFYKRHCISTFSIQHADDMCNDMGLNPWRKSNFLLEAFSPYGSQDYRLGQEEKEDMTA

>Arabidopsis thaliana_GOX1_AT3G14420

MEITNVTEYDAIAKQKLPKMVYDYYASGAEDQWTLQENRNAFARILFRPRILIDVSKIDMTTTVLGFKISMPIMVAPTAMQKMAHPDGEYATARAASAAGTIMTLSSWATSSVEEVASTGPGIRFFQLYVYKNRNVVEQLVRRAERAGFKAIALTVDTPRLGRRESDIKNRFTLPPNLTLKNFEGLDLGKMDEANDSGLASYVAGQIDRTLSWKDVQWLQTITKLPILVKGVLTGEDARIAIQAGAAGIIVSNHGARQLDYVPATISALEEVVKATQGRIPVFLDGGVRRGTDVFKALALGASGIFIGRPVVFSLAAEGEAGVRKVLQMLRDEFELTMALSGCRSLKEISRNHITTEWDTPRPSARL

>Arabidopsis thaliana_SDP6_AT3G10370

MSLASIRRLAAGAAVIAAASGGAVYLSPSVASSDKGGGPILDSLRRRLGDPTASVPSRSAQESALIAATASDPLDVLVIGGGATGSGVALDAVTRGLRVGLVEREDFSSGTSSRSTKLIHGGVRYLEKAVFNLDYGQLKLVFHALEERKQLIENAPHLCHALPCMTPCFDWFEVIYFWMGLKMYDLVAGPRLLHLSRYYSAKESIELFPTLARKGKDKNLRGTVVYYDGQMNDSRLNVGLACTAALAGAAVLNHAEVVSLITDDATKRIIGARIRNNLTGQEFNSYAKVVVNAAGPFCDSIRKMIDEDTKPMICPSSGVHIVLPDYYSPEGMGLIVPKTKDGRVVFMLPWLGRTVAGTTDSNTSITSLPEPHEDEIQFILDAISDYLNIKVRRTDVLSAWSGIRPLAMDPTAKSTESISRDHVVFEENPGLVTITGGKWTTYRSMAEDAVDAAIKSGQLKPTNECVTQKLQLLGSYGWEPSSFTTLAQQYVRMKKTYGGKVVPGAMDTAAAKHLSHAYGSMADRVATIAQEEGLGKRLAHGHPFLEAEVAYCARHEYCESAVDFIARRCRIAFLDTDAAARALQRVVEILASEHKWDKSRQKQELQKAKEFLETFKSSKNAQFNDGKHN

>Arabidopsis thaliana_FOX1_AT1G26380

MKEALFGLYLVLLVSGLEAAVTKPNSGNFIECLRYQASPENPITDAIFTVDNTTTFLSSYVSYTKNTRFSNPNNKNLLAIVVAKDVSHVQATVVCAKSNGIQIRIRSGGHDNEGLSYVSSVPFVILDMHKLRDITVDVSSKKAWVQAGATLGELYVKIDEASQTLAFPAGICATVGAGGHISGGGYGNLMRKFGTTVDHVIDAELVDVNGKLLNRSTMGEDLFWAIRGGGGASFGVILSWKINLVEVPKIFTVFQVNKTLEQGGTDVVYKWQLVANKFPDNLFLRAMPQVVNGTKHGERTIAIVFWAQFLGRTDELMEIMNQSFPELGLRREDCQEMSWLNTTLFWAMLPAGTPKTVLLGRPTDPVFFKSKSDYVKKPIPKEGLEKIWKTMLKFNNIVWLHFNPYGGMMDRIPSNATAFPHRKGNLFKVQYYTTWLDPNATESNLSIMKELYEVAEPYVSSNPREAFFNYRDIDIGSNPSGETDVDEAKIYGYKYFLGNLKRLMDVKAKSDPENFFKNEQSIPPLLSRVRRDDEL

>Arabidopsis thaliana_YUCCA1_

METVVVIVGAGPAGLATSVCLNQHSIPNVILEKEDIYASLWKKRAYDRLKLHLAKEFCQLPFMPHGREVPTFMSKELFVNYLDAYVARFDINPRYNRTVKSSTFDESNNKWRVVAENTVTGETEVYWSEFLVVATGENGDGNIPMVEGIDTFGGEIMHSSEYKSGRDFKDKNVLVVGGGNSGMEISFDLCNFGANTTILIRTPRHVVTKEVIHLGMTLLKYAPVAMVDTLVTTMAKILYGDLSKYGLFRPKQGPFATKLFTGKAPVIDVGTVEKIRDGEIQVINGGIGSINGKTLTFENGHKQDFDAIVFATGYKSSVCNWLEDYEYVMKKDGFPKAPMPKHWKGEKNLYCAGFSRKGIAGGAEDAMSVADDIRSILATLKNN

>Arabidopsis thaliana_GS-OX1_AT1G65860

MAPTQNTICSKHVAVIGAGAAGLVTARELRREGHTVVVFDREKQVGGLWNYSSKADSDPLSLDTTRTIVHTSIYESLRTNLPRECMGFTDFPFVPRIHDISRDSRRYPSHREVLAYLQDFAREFKIEEMVRFETEVVCVEPVNGKWSVRSKNSVGFAAHEIFDAVVVCSGHFTEPNVAHIPGIKSWPGKQIHSHNYRVPGPFNNEVVVVIGNYASGADISRDIAKVAKEVHIASRASESDTYQKLPVPQNNLWVHSEIDFAHQDGSILFKNGKVVYADTIVHCTGYKYYFPFLETNGYININENRVEPLYKHVFLPALAPSLSFIGLPGMAIQFVMFEIQSKWVAAVLSGRVILPSQDKMMEDIIEWYATLDVLGIPKRHTHKLGKISCEYLNWIAEECHCSPVENWRIQEVERGFQRMVSHPEIYRDEWDDDDLMEEAYKDFARKKLISSHPSYFLES

>Arabidopsis thaliana_ZEP_AT5G67030

MGSTPFCYSINPSPSKLDFTRTHVFSPVSKQFYLDLSSFSGKPGGVSGFRSRRALLGVKAATALVEKEEKREAVTEKKKKSRVLVAGGGIGGLVFALAAKKKGFDVLVFEKDLSAIRGEGKYRGPIQIQSNALAALEAIDIEVAEQVMEAGCITGDRINGLVDGISGTWYVKFDTFTPAASRGLPVTRVISRMTLQQILARAVGEDVIRNESNVVDFEDSGDKVTVVLENGQRYEGDLLVGADGIWSKVRNNLFGRSEATYSGYTCYTGIADFIPADIESVGYRVFLGHKQYFVSSDVGGGKMQWYAFHEEPAGGADAPNGMKKRLFEIFDGWCDNVLDLLHATEEEAILRRDIYDRSPGFTWGKGRVTLLGDSIHAMQPNMGQGGCMAIEDSFQLALELDEAWKQSVETTTPVDVVSSLKRYEESRRLRVAIIHAMARMAAIMASTYKAYLGVGLGPLSFLTKFRVPHPGRVGGRFFVDIAMPSMLDWVLGGNSEKLQGRPPSCRLTDKADDRLREWFEDDDALERTIKGEWYLIPHGDDCCVSETLCLTKDEDQPCIVGSEPDQDFPGMRIVIPSSQVSKMHARVIYKDGAFFLMDLRSEHGTYVTDNEGRRYRATPNFPARFRSSDIIEFGSDKKAAFRVKVIRKTPKSTRKNESNNDKLLQTA

>Persicaria tinctoria_LC585869.2

MERKVGIIGAGISGLLACKHALSKGFHPVVLEAQPDIGGVWANALETTRLQTPKDFYQFSDFPWPSSVQDMHPTGEQVMEYIRSYADHFGLLKHVRFNTKVLSISFEGTSDQEMEAWSHWNGTGEGFANKGKWTLLLQDVPTQSQQVMEFNFVILGIGKFSDRMRMPEFPAGEGPEVFDGKVIHSKDYSEKSYGDARRLVKGKRVVVVGFQKSAIDIANECSSANGKEVPCNLIYRTPPWNIPDFFIWGLPLPYLYFNRFSELLLHKPGEGLFLSLLATILSPLRWLIAKFVESNIKYRHPLKKYGLVPEHGFGEAISSCVLSVLPKGFYENLEKGSILLNKAEKFRFCKEGIVMEGKPKPLEADLVIFATGFEADEKLRDIFASPKFQGHIMGSPHSILPLYRQCIHPQIPQLAIIGYSESFANLYTSEMRCRWIVELLDGTFKLPGFKEMEKETANWNKFIKGNAPKHYRRSCIGQLHIWYNDQLCKDMGWNPRRKKGFISEWFQPHGPLDYIG

>Aphelandra squarrosa_Bx2/Bx3_TRINITY_DN6556_c0_g1_i1

MQMKKTVAIVGAGISGLLACKYALSIGLNPVVFEAESCVGGLWNHTIECTRLQSLKDYFQFSDFPWPSTIKTMFPKNEQVLEYVNSYADHFQLRQYVRFNSQVMSIGYEGESKEEMGSWKSWGGTGMAFGSKGKWRLQVEAGGGMQEYHVADFVIVCIGKFSGLPAVPDFPPGSGPEVFSGKVLHSMEYSDMDNASAAQFIKGKRIAVIGSGKSAIDIAFECGRANGRDTPCTVIHRNAHWMLPYEKPWLLAFALLCFTRFSELLVHKPGEGFFASVLATLLSPLRWLVSKFLEISLRWRLPLKKYDMIPSENFLEEANSCQVIMLQDNFFDKVVDGSIVFKKSEHFSFCKEGLIVEGEDSPVEVDIVIFATGYKGDEKLKNIFASPAFQNHIFGSRNSTVPLFRQMIHPRIPQLAVVGYSESLSNLYTFEMRCRWLAFFLDRAFALPSIKEMEDDIKMWERYMKKYAGNGEFKRGCIAGIHIWYNDQLCKDIGCSPRRKKGISSELFQPYGLEDYDGITPAQINPSYFAK

>Lamium galeobdolon_Bx2/Bx3_TRINITY_DN81_c0_g1_i13

MLMEKRVAIIGAGISGLLACKYTASIGLKPIVFEEQDNVGGLWNHTIEITRLQQVKQSFQFSDFPWPSSADDNPKSAQLLEYLQSYAHNFDLLPFIKFNSKVMNIDYVGESKEEMQSWDLWGGSGKAFGSKGRWILKVLHTKDDSIKVYESEFVVVCTGRFSGLPNIPEFAPGYGPEIFSGQALHSMDYSNMDNASAAQFIKGKRIAIIGSGKSAFDIAFECANANGNDNPCTLVQRTIHWTFPSTKTWGLNFAFLIFTRFSELMVRKPGQGFVSSALTTLLTPLRWGMSKYVESYLRWKLPLKKYGMVPKESFAAESSACQIFYLPRGNFYGKVEDGSIVLKKSQEFRFCKHGLIIDGDDKNPINADIVIFATGYKGDEKLKSIFASPTFQNYIVGSPTSTVPLYRQMIQPRIPQLAVIGYSESFSNLFTFEMRCKWLAFFLDQAFELPSIRAMKKEVEAWETYMKRYAGNNKFRRACIGRVHVWYNDQLCRDIGCNPRRKKGFLSELFEPYGSADYVGL

>Consolida orientalis_Co_10

MEGRVGIIGAGLSGLLACKYVSQMGFTPVVFEAKAEIGGVWNQTIETTKLQTVKQYYQFMDFPWPSSVKEDFPDHNQVLDYIESYARHFGLLQYIKFNSRVTSIDFEGVSDKEMSSWDFWGGNNNPFGSKGKWNITVEDTLTQSTEVYQVKFVILCIGRFSDVPKIPDFPPKRGPEAFDGKVIHAMEYAARGAECIRGKRVAIVGIQKSALDIAVECAAENGLEHPCTMIYRRAHWNFPDYYPWGVPLALLYFNRFAELLIHKPGEGYLLSLLATLLSPLKWSRTKFVESYLKWKLPMKKYDMLPEHSFDRDLSASTISITPETFYEKVKKGSIVLKKSSGFSFCKKGLMLDGVADPLEVDVVILATGYKSDEKLKDIFASSTFKDHIMSSSIQFYRECVHPRIPQLAVIGYSESLFNLYPFEMRCRWLGHLLKGMFKLPRIKEMEMDVNRWEQYMKKYTGEYHRRSSSSWIQTWCNDQLCKDMKCNPRRKDGVLAELFEPYGPMDYADLS

>Consolida orientalis_TRINITY_DN1290_c0_g2_i1

MEKKQVGIIGAGLSGVLACKYVLEMGFTPVVFEAKSRVGGVWTQTIQTTKLQSNKKSYQFTDFPWPSSVKEAFPDHNQVNEYIKSYACHFGLLQYIKFDSRVTAIDYEGVSNEEMASWDSWGGTGDAFNPKARWSIAVENTRTQSTALYHVDFLILCIGRFSEVPNIPDFPPNKGPEAFDGEVIHSIEYAARGIEIIKGKWVTVIGGLKSALDIASECAAENGVEHPCTMIYRKAHWSFPDYFPWGVPLVRLYFNRFAELMIHKPGEGFMLSILATLLSPLRWLVTKFVESYIKWKLPLKKYNMVPEHSFSTELASCTLTVSPENFYNKVEQGSIVLKKSATFSFCMKGLMTDGATAPLETDIVILATGYRGDQKLKDIFASSIFHNYIPTSSKTVPLYRECIHPRIPQLAIIGYSESLVNLYVFEMRCRWLGHFLKGTFKMPRLREMEMDVERWEIFRRQYSGDYYKRSCLSLINIWFNDQLCRDMRCNPRRKTGIFAELFEPYGPMDYVNLCPQ

>Consolida orientalis_TRINITY_DN4699_c0_g1_i4

MEGRVGIIGAGLSGLLACKYVSQMGFTPVVFEAKAEIGGVWNQTIETTKLQTVKQYYQFMDFPWPSSVKEDFPDHNQVLDYIESYARHFGLLQYIKFNSRVTSIDFEGVSDKEMSSWDFWGGNNNPFGSKGKWNITVEDTLTQSTEVYQVKFVILCIGRFSDVPKIPDFPPKRGPEAFDGKVIHAMEYAARGAECIRGKRVAIVGIQKSALDIAVECAAENGLEHPCTMIYRRAHWNFPDYYPWGVPLALLYFNRFAELLIHKPGEGYLLSLLATLLSPLKWSRTKFVESYLKWKLPMKKYDMLPEHSFDRDLSASTISITPETFYEKVGNGSIVLKKSSGFSFCKKGLMLDGVADPLEADVVILATGYKSDEKLKDIFASSTFKDHIMSSSIQFYRECVHPRIPQLAVIGYSESLFNLYPFEMRCRWLGHLLKGMFKLPRIKEMEMDVNRWEQYMKKYTGEYHRRSSSSWIQTWCNDQLCKDMKCNPRRKDGVLAELFEPYGPMDYADLS

>Consolida regalis_TRINITY_DN1728_c0_g2_i1

MNSKQTSSMEGQVGIIGAGLSGLLACKYVSQMGFTPIVFEARAGLGGVWNQTIETTKLQTVKQYYQFMDFPWPSSVKEDFPDHNQVLDYFESYARHFDLLQYIKFNSRVTSIDFEGVSDEEMSSWDVWGGNNDPFGSRGKWNITVEDTLMQSTQVYQVKFVTLCIGRFSDVPNIPHFPPNKGPEAFDGKVIHSMDYAANGVESIKEKRVTVVGIQKSALDIATECAAENGVGHPCTVIYRRAHWNFPDYFPWGVSLALLYFNRFSELLIHKPGEGFVLSLLATLLSPLRCLRSKFVESYLKWKHPMKKYNMIPEHSFDQDFSSCTTSITPETFYKKVEEGSIVLKKSSIFSFCKKGLMLDGVAAPLETDVVILATGYKGDKKLKDIFASSTFKDYIMSSSSTIQLYRECVHPRIPQLAFIGYSESLFNLYPFEMRCRWLGHLLIGTFKLPRIKEMEIDVKRWEQYMKNYTGEYYRRSCSSLIQTWCNDQLCNDMKCNPRRKDGLLAELFEPYGPMDYADLSPQ

>Clematis_armandii_CNA0013873_151485

MEGKRVAIVGAGISGLAACKQAVDRGFDPTVFESERGVGGVWARTLDSTRLQSPRTAFQFSDFPWPPSVAEVFPDHLQVMDYLRAYAAEFGLLRRVKFDHRVLGIDYVGAGEAEIAAWEMWAGNGEAFGGGGKSRGEWHLTVQHGYETEIYHADFVILCIGRFSGVPNIPEFPAKKGPENFDGKIIHSMDYSNMGTAKASELIRDKLVTVVGYLKSAIDIASECADVNGTKYPCTMICRTKRWIIPDYYAWGVPLAFFYLNRFSELLIHKPGEGFLLCLLATILSPLKWLLSKFTESYYKWAIPMKKHGMVPDHSFFQAITSCLIAILPENFYSRVDEGSIVLKKAKEFSFCKDGVMVDGEDTPIKSDLVIFATGFKGDQKLKDIFKSPFFRNIVAGSSSTTVPLYRECIHPRIPQLAIIGYSESLANLYTSELRSKWLAHFLDGGFKLPSIKNMEKSIMEWEKFMKKYSQDYFRRSCIGTLHIWYNDQLCRDMGCNPRRKKGFFAEWFEPYGPQDYVDVEPKKKK

>Hydrastis_canadensis_VGHH_scaffold_2006520

MERRVAIIGAGISGLLACKYTSEKGFDPIVFEAKNGIGGVWTHTVETTKLQTPKEAYQFSDFPWPSSVKEEFPDHNQVLEYIESYSRHFDLLRYIKFNSKVINIDYEGVSEDELVSWDLWGSTGEPFSPKGKWNITVEDIQKQSTEVYQVEFVILCIGRFSDVPNIPDFPLNKGPEAFNGKVIHSMDYSAMDDASAAEFIKGKRVTVVGIQKSGLDIAAECAMANGLEYPCTVIYKTAHWIFPNYFPWGVPLALLYLNRFAEFMIHKPGEGFLLSLLATLLSPLRWLVSKFVESYIKWKLPLKKYNMIPKHSFDKDISACTLAMIPDNFYDRVEEGSIILKKSTNFSFCKDGLMIDGDNAPLKTDIVIFATGYKGDQKLQNIFVSPTFQKYIMGSPRTTIPLYRECIPPRIPQLAIIGYSESLVNLYTSEMRCRWLAHFLDGTFKMPSVKEMEEDVLRWEKYMKRYSGEYYRRSCLSVLHIWYNDQL

>Coptis_deltoidea_CNA0013856_25340

MEKSVGIIGAGISGLLACKYVLEMGFNPIVFEATNGIGGVWDHQTVKTTRLQTHKLFYQFSDFPWPSSVLEDLPDHKQVKEYIESYASHFDLRKYIKFNSKVISIDYEGVFDEEMVSWDLWSGTGEPFSPKGKWNVTVEDLDLQSTKDYQIEFVILCIGKFSDVPNIPEFPPNKGPEVFGGKVVHAMDYSAMDDASAAEFIKGKRVTVVGLQKSGLDIATECAMANGVEHPCTVIYKKAHWSFPDYLPWGVPLALLYFNRFAEFMIHKPGEGLVLSLLATVLSPLRWLRSKFVESYLKSKLRLKKYNMTPEHSFNKDFACCTLAVTPDKFFDRVEEGSIILNKSTTLIFSKDGLMIDGKTAPLETDIVILATGYRGDQKLHNIFASSTFQKYIIGSSRTRIPLYRECIHPRIPQLVIIGYSESIANLFTSEMRCRWLAHLLKGTFKLPRITEMEKDVLRWEKFMKQYQGEEYYQRSSISISHIWYNDLLCKDMKCNPRRKEGFFADLFEPYGPMDYVNLNPQ

>Coptis_chinensis_CNA0013793_84479

MEKSVGIIGAGISGLLACKYVLEMGFNPIVFEATNGIGGVWDHQTVKTTRLQTHKLFYQFSDFPWPSSVLEDLPDHKQVKEYIKSYASHFDLRKYIKFNSKVISIDYEGVFDEEMVSWDLWSGTGEPFSPKGKWNVTVEDLDLQSTKDYQIEFVILCIGRFSDVPNIPEFPPNKGPEVFGGKVIHAMDYSAMDDASAAEFIKGKRVTVVGLQKSGLDIATECAMANGVAHPCTVIYKKVHWSFPDYLPWGVPLASLYFNRFAEFMIHKPGEGLVLSLLATVLSPLRWLRSKFVESYLKSKLRLKKYSMTPEHSFNKDFACCTLALTPDKFFDRVEEGSIILNKSTTLSFSKDGLMIDGKTTPLETDVVILATGYRGDQKLHNIFASSTFQKYIIGSSRTRIPLYRECIHPRIPQLTIIGYSESIANLFTSEMRCRWLAHLLKGTFKLPRITEMEKDVLRWEKFMKQYQGEYYQRSSISISHIWYNDLLCKDMKCNPRRKKGFFADLFEPYGPMDYVNLNPQ

>Aconitum_carmichaelii_CNA0013802_240769

MEKRVAIVGAGISGLAACKYISQKGYNPVVFESQTGIGGVWTHTVETTRLQTPKDFYQFSDFPWPSSVKEEFPNHNQVMEYLQSYAHHFNLLPYIKFNTKVVAINYEGPPCEEVATWDLWSGTGEPFSPRGKWNITVQHTQNKSTQVYQVEFVVLCIGRFSNVPNIPDFPPNKGPEAFNGQVIHSMDYSAMDDTTAAKFVKGKRVTVIGLQKSALDIASECATVNGEKNPCTVIYKTEHWNVPDYLPWGVPLAYFYLNRFAELMVHKPGEGLLLSLVATMLSPLRWGFSKFVESYIRSKFPLKKYKMIPKHSFHQEISSCLISTVPEKFYDKVEEGSISLKKSASFWFYKEGLMLDGHTAPLETDLVILATGYKSDVNLKNIFIPPFFQECIMGSPTNTIRLYRECIHPRIPKLAILGYSESLSNLYTTEIRCKWLAQLLEGTFKLPHIKEMENDTLKWEKYMKRYSGKYYRRSCLAVVHIWYNDQLCKDMGCNPKRKKGILAELFEPYGGTDYADIS

>Anemone_hupenhensis_ZUHO_scaffold_2014957

MARRVAIIGAGISGLLACKYISEKGFNPIVFEAQSGVGGVWIKTVETTRLQTPRDSYQFSDFPWPSSVKDEFPNHDLVLEYLESFTRHFELYRYIKFYSRVLAIEYEGPSEQEMLAWDLWGGTGEPFSPKGTWNVTVQNTQDLSTEIYQVEFVVLCIGRFSTLPNIPDFPPNKGPEVFDGKVIHSMDYSAMDDAAAAEFVKGKRVTVVGLQRSALDVASECATVNGVKLPCTLVYRTEHWNVPDYLPWGVPLEYLYCNRLAELMVHKPGEGFLLCLLATMLSPVRWVFSKFVESHIRWKFPLKKYKMIPKHSFHQEISTCLISTIPSKFYDKVEEGSIVLRKSPAFTFCKNGLMVEGETAPLETDLVILGTGFKSDVKLKNVFTSPTFQKYIMGEPTSTIRLYRECIHPRIPQLAVIGYSESLSNVYTSEIRCQWLAQLLDGRFTLPSIEVMEEDTLKWEKYMKRYSGPYYRRSCLGVVQIWYNDLLCKDMGCNPRRKKRFLAEIFEPYGSTDYANLFE

>Beta vulgaris_XM_010691953.4

MERKVGIIGAGISGLLACKYALAKGYHPIVFESRSTIGGVWTKTVETTKLQTPKPLYEFSDFPWPSSVETVFPDQNQVFDYLKSYANHFDLLKHIRFNTKVVSIKYEGPSDEEIQSWALWGGNGDPFGNKGKWVVTTQDLKNQSTEVMQVDFVILCLGRFSDVPKMPEFPSGKGPEVFAGEVMHSMDYSAMDFESARNLVKGKQVTVVGFQKSALDIAMECSIANGVENPCTVICRTPHWNVPDYFPWGFPLANLYLNRFSEFLVHKPGEGALLGLVASLLSPLRWGISKFVESDIKKKHQLEKFGMVPEHSFLQEMNSCLISTVPQGFYDNAEKGSIILKRSPTFTFCNEGVLLDDRVGTKEPIKSDLVIFATGFRGDQKLKDIFASPKFQDYIFGSSNNAVPLYRECIHPRIPQVAVIGFSESIANLYTSELRCRWLFELLDGKFKLPNVQKMEKDVLEWDKFMKRYSGQYYRRSCIGALHIWYNDQLCKDMGLNPKRKKGLWAEMFEPYGPMDYASTV

>Vitis vinifera_XM_002278560

MERKVAIIGAGISGLLACKYTLSKGYTPVVFESRSSIGGVWTQTFETTKLQTPRSVYQFSDFPWPSSVPEFPNHSQVLDYIVSYATHFNLLPHIKFNSEVLGIEYEGPSDEEVQAWELWSGTGEPFNSKGKWSITVKDTNSLSTEVYQVGFVILCVGQYSDVPKIPEFPAGKGPEAFRGKVIHSMEYAAMDFERAAEFIKGKRTTVVGFQKSALDIAMECSAANGALNPCRLLYRTEHWNIPDYYPWGVPLAYLYFNRFAELLVHKPGEGFFLSLLATILSPLKWGFSKFVETYVNQKLSLAKFGMVPEQSFHKDISSCLISSMPEEFYDRVEKGSIILKKAPKFSFCKEGIVVDGEASPLESDLVILATGFKGDEKLKDIFVSPTFQNHIIGSPNASIPLFRQCIHPRVPQLAVIGFSESVSNLYTSEMRSRWLAEFLDGTFKLPSIKEMEKDAERWDQYLKRCSEKHYRRSCIAALHIWYNDQLCKDMGWNPKRKKGFFAELFEPYGPLDYRPIS

>Phytolacca_acinosa_CNA0013870_73975

MERKVGIVGAGISGLLACKYALSKGYHPLVFESQDSIGGVWTKTIETTKLQTPKPLYQFSDYPWPSSVETVFPDHNQVFDYLQSYANHFDLLKYIRFNTKVVSIKYEGPTDEEMQSWALWGCVGVPFGNKGKWIVTVQDQLTLSTEVMQVDFVILCVGRFSDIPNMPEFPPGKGPDAFEGKVIHSMEYATMDYQSARNLVKGKRITVVGFQKFALDIAMECSLANGVEHPCTVICRTPHWNLPDYFPWGFPLAKLYLNRFAELLIHKPGEGPFLSLLATLLSPLRWGIIKFVESDIKKRHHLEKFNMVPEHNFLQEINSCLVSTVPDGFYDNAEKGSIILKRSQTFTFCKEGVLLGDDGELVKSDLIIFATGFKGDQKLKDIFVSPKFQDHIMGSTDTIVPLYRQCIHPRIPQLAVIGFSESVANLYTSEVRCRWLAELLDGKFKLPSIKEMEKDVLEWDKFMKKYSGKYYRRSCIGALHIWYNDQLCKDMGWNPKRKKGLWAELFEPYGPMDYASQ

>Pistacia vera_XM_031413620

MEKKIAIIGAGISGLLACKYVLSKGFHPIVFEARSSIGGVWIKTVETTKIQTPKPAYQFSDFPWPSSVTVDFPSQHQVLDYIQSYAHHFDLLKHIKFNTKVVGIEYEGASDEEIGSWSSWNGSGTPFSSQGKWKVVAEDIQSHSTEVYQVDFVILCLGRFSDVPNMPEFPPNKGPEAFHGKVIHSMEYVAMDYKSAAEFLKGKRVTVVGFQKNALDIAMECSAANGLEFPCTVMYKTEHWNVPDYLPWGVPLAYFYLNRFSELLVHKPGEGILLSLLATLFSPLRWAFSKFVESHIKHKLRLAKYGTVPKHSFLKELNSCLIATVPEKFYDKVDEGSIILKKSPSFSFCEEGILVNGETEPLKTDLVILATGFRGEKKLKDIFVSQTFQDYILGSPKEAVPLYREIIHPRIPQLAVIGFSESVANLYTSEVRCQWLAELLYGTFILPNIKEMEKDIKKWDEFMKQSSGEYYRRSCIGGLHIWYNDQLYKDMGRNPKRKKGFFAELFEPYGPSDYLPSSRSN

>Sesamum indicum_XM_011080251.2

MRRISSIQAQATLKSSCKIQELLSSSMEKRVAIIGAGISGLLACKYAASRGFNPVVFEAEEQVGGLWNHTIESTRLQNIKDFFQFSDFPWPSSVQDAQPTNTQLLEYLQSYARHFELLPYIKFNSKVVSVSYVGECEEEIQSWELWGGSGKAFGSKGKWNLKVLHTKEESVEEYAAEFVVICIGRFSGLPEIPSFPPGFGPEVFSGKVLHSMDYSAMDNASAAKFIKGKRIAIIGSGKSAIDIAFECAKANGNDIPCTVIQRTIHWMLPHDAQPLGLSFGFLCFSRFAELLVHKPGEGFLSSILATSLSPLRWGMSKLIESYLRWKLPLKKYNMIPKEDVLQEKSSCQILFLPPNFYDKVEDGSIILKKSQCFSFCKEGLILDGEDDPVKADIVIYATGYKGDEKLKNIFSSPTFQNCIAGSPTSAIPLYRQMIHPRIPQLVVISYSESLSNLYTFEMRCKWLGYFLDEAFKLPSIKKMEEDIKMWEKYMKKYSGNGKFRRACISGVPIWYNDQLCKDIGCNPRRKKGFFTDLFEPYRLADYKEL

>Coffea arabica_XR_003449871.1

MEKRIAIIGAGISGLLACKYALAKGYHPTVFEAQDCAGGLWNHTIESTKLQNPKEVFSFSDFPWPSSVKETFPRNTEVLKYVQSYAQHFGLLPYIKFNTKVIGIDYVGVSREEMQAWDLWGGTGQAFGSKGKWNIKVQHTTEDKSIKEYEVEFVILCIGRFSGVPNIPEFPPNHGPEIFGGKVLHSMDYSAMDNASAAALVEGKNIAIIGSEKSAVDLAAQCANVNGIDNPCTMVHRAAHWMLPSYYVWGGVRIDYLYYNRFAEILVHKPGEGFLHSALAFLLSPLRWGIAKLVESYLRWKFPLKKYNMIPPHGFLQDVFSCEFLMLPENFFDRVVDGSIVLKKSKNISFSREGLMIDGETQPLKADVIILATGYRGDQKLGNIFTCPSFQEYILGSTSSTIALYRQIIHPRIPQLAVIGYSESLNNLYTLEMKCRWLGHFLDQTFQLPSIKEMEKDVRMWDKYMKRYAGKYYRRSCLGASHIWYNDQLCKDIGCNPRRKKGFFSELIEPYGPADYAELNPRS

>Aphelandra squarrosa_TRINITY_DN8200_c0_g1_i3

MEKRVAIIGAGISGLLACKYAVSVGLRPTVFEEQHHTGGLWNHTIESTRLQNGKEFFQFSDFPWPTSVEDLFPKNTQVLEYVQSYAHHFQLLQFIKFDSRVLSIDYVGESEEDMESWELWGGAGKAFGSKGKWNLKVFQTEEGGSIKEYVAEFVVLCIGRFSGLPAIPEVPPGHGPQVFSGKVLHSLDYSAMDNAAAAQFIKGKRVAIIGSGKSAIDIAFECALANGSEKPCKVIQRTTHWMSPDAQPWGVSFGHLCFTRFSELMVHKPGEGFVTSVLATLLSPLRWGMSKFVESYLKWKLPLQKYAMIPEESFVQEASSCQIFFLPENFYDKVVDGSIVFKKSEHFSFCKEGLIIDGEDSPIEADIIIFATGYKGEEKLKNIFASPTFQNYILGSPNTIVPLYRQMVHPRIPQLAVIGYSESLANLYTFEIRCKWLAFFLDQTFHFPSITEMEGDVKMWEKYMKKYAGNGKFRRGCIGGVHIWYNDQLCKDIGCNPRRKKGVLSELFEPYGLADYSGISSGNRFMFNPPL

>Leonurus cardiaca_LECA_c33258_g1_i2_len_1832

MEKNRVAIIGAGISGLLACKYIASIGLNPIVLEAQHNIGGLWNHTIESTRLQQNKQTFQFSDFPWPSSADDNPNSAQLLEYLQSYAQNFDLLRYVKFDSKVMSVDYVGESQEEMQSWELWGGAGKAFGSKGRWILNVLDAKDNSIKEYEAEFLVLCIGRFSGLPNIPEFAPGYGPEIFSGQVLHSMDYSAMDNATAAQLIKGKRIAIIGSGKSAFDIAFECANANGNDNPCTLVQRTIHWAFPDARPWGVDYGFLVLNRFSELMVHKPGEGILSSVLATLLTPLRWGMSKFVESYLRWKLPLKKYGMIPKERFVAGASACQIFFLPHENFYDKVEDGSIVLRKSQQFSFCKEGLIINGDPINADIVIFATGYKGDEKLKNMFASPSFQNYIIGSPNSAVPLYRQMIQPRIPQVAVIGYSESFSNLFTFEMRCKWLAFFLDQAFELPSIRAMETDIEAWEKYMKRYAGNHKFRRACIGCVHVWYNDQLCRDIGCNSRRKKGLFSELFQPYGSKDYDGLP

>Lamium album_LAAL_c33795_g1_i2_len_1839

MEKRVAIIGGGISGLLACKYAAAKGFAPTVFEEQDNVGGLWNHTLESTVLQVAKEFYQFSDFPWPDSADMFPHNTQLAQYLQSYAHKFQLLQFVNLNSKVMNINYVGESEQEMQSWDSWGGVGKAFGSKGKWILRVLHKEDDSIKEYEAEFLVLCIGRFSGLPNKPEFELGYGPEEFSGQVLHSMDYANMENAAAADLIKGKRVVVIGSGKSAVDIAFECANVNGKDNPCTMIQRTVHWMLPHDSQPWGLSFGFLCFTRFAELMVHKPGESFLSSAAATVLAPLRWGLNKLVEIYVKWRLPLKKYDMIPTESFVGEASSCQILFLQPHFYDKVEDGSILLRKSHNFTFCKEGVILDGEGTPLQADVVILATGYKGDEKLKNMFASPTFQDYIEGSPTSILPLYRQMIHPRIPQLAVIGYSEALSNIFTFEMRCKWLAQFLDESFRLPSIGAMEKEIEMWDKYMKRYAGNGKFRRSCIGGVHIWYNDQICRDIGSTPRRKKGFFSELFEPYGPADYHGL

>Tectona grandis_TEGR_c41247_g1_i3_len_1839

MGKRVAIVGAGIGGLLACKYTASIGLKPILFEEQDQIGGLWNHTIESTRLQVGREVFQFSDFPWPSSADKIPHSPQLLEYLRSYAHNFELLQYIKFNSKVINIDYVGESEEEMQCWELWGGVGKAFESKGKWILKVFHTKEDTTEEYEAEFVVLCIGRFSGLPDIPELSPGCGPEIFSGEVLHSMDYSAMDNATAAELIQGKRVAIIGSGKSAIDIAFECAKANGNDKPCTVIQRTIHWMLPDIKVWGVSYEFLCFTRVIELMVHKPGEGFLSSVVATLLTPLRWAASKFVESYARWKLPLRKYNMIPKESFVRDASSCQAFFLPHENFYDKVEDGSIVLRKSQHFRFCKEGLILDGEDDPLKADIVIFATGYKGDEKLKNMFASPTLQNYIAGTPTSTIPLYRQMIHPRIPQLAAIGYSESLSNLYTFEIRCKWLAYFLDQVFHLPRIKAMEKEIETWEKYMKRYTGNSMFRRSCIGLIHIWYNDQLCKDIGCNPRRKKGFFAELFKPYGSVDYKELAPGL

>Rosmarinus officinalis_ROOF_c40410_g1_i1_len_1849

MEKRVAIVGAGISGLLACKYAVARGFTPIVFEEQDHVGGLWNHTLESTTLQVPRQLYEFSDFPWPSSIEEMFPHNTQLTEYLRSYAQKFQLLQYVNFKCKVIDVDYVGESEDEIKCWDLWGGDGKAFGSKGKWILRVSHAADESIKEHEAEFLVLCIGRFSGLPNIPEFATGYGSDLFSGEVMHSMDFANMENAAAAQFIKGKRVVVIGSGKSAVDIAYECANVNGRDNPCTMIQRTIHWMLPHDAKPWGLSLGFLCFTRFAELMVHKPCEGFLYSAAATMMSPLRWVVNKLVESYLKWRLPLKKYDMVPTESFVGEASSCQILFLQPNFYDRVEDGSIVMRKSQNFTFCKEGVILDGEGVPLQADIVILATGYKGDEKLKNLFASPTFQNYIFGSPTSTIPLYRQMIHPRIPQLAVIGYSESLSNIFTFEMRSIWLSHFLDGAFALPSIGAMEKEIETWERYLKRYSGNNSFRRSCIGGVPIWYNDQICRDIGSTPRRKKGFFSELFEPYGPADYQGL

>Salvia hispanica_SAHI_c28219_g1_i1_len_1864

MDEKRVAIVGAGISGLLACKYAAAKGFTPVVLEEQDKVGGLWNHTLESTTLQVPKQLYEFSDFPWPSSVQELRPHNTQLVEYLQSYAHKFQLLHYVKFNTKVIDVDYVGGSKEEMNCWGGDGKAFASKGKWILRVSHSGDHHDSIKEYEAEFLVLCIGKFSGLPNIPDFAPGYGSDAFSGKVMHSMDLAKMENAAAAQLIKGKRIVIIGSGKSAVDIAYECANLNGRDNPCTMVQRRIHWMIPHDAQPWGLSFSFLFFTRFAELMLHKPGQGFFFTAATILLTPLRWVVNKLVEIYLKWRLPIKKYDMVPTENFIGQASSCQIHFLQPNFYDRVEDGSILMKKSKTFTFCKEGLILDGEALPLHADVVIFATGYKGKEKLKNMFASPTYQNYIFGSPNSIVPLYRQMIHPRIPQLAVIGYSESLSCLFTFEMRCKWLSHFLDGEFGLPNIRDMEKEMEGWEKYMKRYAGKGRSCIGGVPIWYNDQLCRDIGSTPKRKNGFVSELFQPYGPADYHHL

>Lamium maculatum_Cluster-9204.5432

MVFEAQDNVGGLWNHTIESTRLQVGREVFQFSDFPWPSSADMIPHNSQLVEYLQSYAQNFDLLPYIKFNSKVMSIDYVGESEQEMQSWELWGGAAKAFGSKGKWHLKVLDTKENSVKDYETEFVILCIGRFSGLPNIPQLSPECGPEIFSGKVMHSMDYSAMKNSDAAQFIKGKRIAIIGSGKSATDIAFECANANGNDKPCTMIQRNVHWGLPNLYPLGIKFTYVFFTRFPELMVHKPGEGFLANILATLLTPLNMLMSKLVESHIRWKLPLKKHGMIPKESIARDAASSCRALVLPDEKFYDKVEDGSIVLKKSQQFSFCKQGLIIDGDDKNPIYADIVIFATGYKGDEKLKNIFASPTFQTYITASPTSILPLYRQMIHPRIPQLAVIGYSESFSNLYTSEIRSIWLAHFLDEVFQFPSIKAMEEEIETWENYMKRYAGNGGFRRACIAGVQIWYNDQLCRDIGCNPRRKKGFFSELFEPYTSVDYKDLAPNEPLIK

>Lamium orvala_TRINITY_DN6071_c0_g3_i2

MEKRVAIIGAGISGLLACKYTASIGLKPIVFEEQDNVGGLWNHTIESTRLQQIKHSFQFSDFPWPSSADDNPKSAQLVEYLQSYAQNFDLLPYIKFNSRVMNIDYVGESKEEMQSWDLWGGAGKAFGSRGKWLLKVLDIKEGSVKEYEAEFVVLCTGRFSGLPNIPEFAPGYGPEMFSGQVLHSMDYAAMENASAAEFIKGKRIAIIGSGKSAFDIAFECAKANGNDNPCTLIQRTINWTFPNAQPWGMNYNFLVLTRFSELMVHKPGEEFLYSALATLLTPLRWGISKLVENYLRWKLPLKKYGMVPKESFVAGASACQIFLLPHENFYDKVEDGSILLKKSQQFRFCKQGLIIDGDEKNPINADIVIFATGYKGDEKLKNIFASPTFQNYIAGSPTSTIPLYRQMIQPRIPQVAVIGYSESFSNLFTFEMRCKWLAFFLDQALELPSIASMEKEVEAWETYMKTYAGKHKFRRACIGCVHVWYNDQLCRDIGCNPRRKKGFFSELFEPYGSADYVGMGTS

>Achantus ilicifolius_TRINITY_DN1050_c0_g1_i3

MAKRVAIIGAGISGLLACKYALSIGLDPVVFEAESYVGGLWNHTIECTRLQSHKDYFQFSDFPWPNSIKSNYPKNEQVLEYVNSYADHFNLFQYIHFNSRVTSISYEGESKDEMAKWTSWGGAGTAFGSQGKWRLQVHVQPSGDFQFHDADFVIVCIGKFSGLPSVPEFPPGRGPEVFSGKVLHSMDYSDMDNGSAAEFIKGKRVAVIGSGKSAIDIAYECGRANGSEIPCTVIHRNAHWMLPYDLPWVVAFGLLCFTRFSEMMVHKPGETFFATILATLLLPLRWLMSKILEISLQWKLPLKKYDMIPKENFLEEANSCQVIMLQDNFFDWVVNESIVFKKIENFSFCKEGLIVDGEDKTIEVDIVIFATGYKGDEKLKSIFASHTFQNNIFGLPNSTVPLYRQMINPRIPRLAVVGYSESLSNLYTFEMRCRWLAFFLSGAFPLPSIKEMEDDIRMWERYMKKYAGNGEFKRGCIAGIHIWYNDQLCRDIGCNPMRKSGILAELFEPYGMADYDGITPARWMKSSYFNK

>Achantus ilicifolius_TRINITY_DN6034_c0_g1_i4

MAKRVGIVGAGMSGLLACKYAVSVGLRPMVFEAQQRVGGLWNQAMESTRLQIDKAHYQFSDFPWPSSVEGDFPKNTQVSEYAESYAHHFQLLDYVKFDSRVISIDYVGESHDEMERWELWGGSGKAFASKGKWILKVEQPGSVQEYEVEFVVLCTGRFSGLPDIPEFPPGHGPDVFTGEVIHSMDYSAMDNARAAEFIKGKRIAVIGSGKSAIDLAFECACANGKSYRQFSCHCSKLKAYTFTSLPGRDQPCILIQRTSHWMFPSTRLWLFLYGFLFFIRFSELMVHKPGEGLFFSILATVLSPLRWLLSNCIESGLRWVLPLKKFGMIPNESFVQEASSCQLLFLPENFFRKVEDGSIVVKKLQHFSFCKEGLIIDGIKQPIEADIVIFATGYKGDEKLKNIFASPTFQSYIFGSPNSIFPLYRQMVHPLIPQLAVLGYSESLSNLYTFEIRSKWLAFFLDQAFHLPSIQQMEEEIKMWDRYMKKYAGNSKFRRNCIGGIHIWYNDQLCRDMGFNPRRKNGVFSELFETYGPEDYSEINPAQQIL

>Achantus leucostachyus_TRINITY_DN9077_c1_g1_i1

MATAKRVAIIGAGISGLLACKYALSIGLDPVVFEAESSVGGLWKHTIECTRLQSHKDYFQFSDFPWPDSIKSNFPKNEQVLEYINSYADHFKLVHYVRFNSRVTSIAYEGESKDEMAAWTYWGGAGTAFGSQGKWRLQVDASSGDVQYHDADFVIVCIGKFSGLPSVPEFPPGRGPEVFSGKVLHSMDYSDMDNASAAEFIKGKRIAVVGSGKSAIDIAFECGRANGGDKPCTVIHRNAHWMLPYDLPWVVAFGLLCFTRFSEMMVHKPGEGFFASILATLLLPLRWLMSKILEMSLEWKLPLKKYDMIPKERFLEEANSCQVIMLQDNFFDWVVNGSIIFKKIENFSFCKQGLIVEGEEKPIEVDVVIFATGYKGDEKLKNIFASQTFQNSIFGLPNSTVPLYRQMINPRIPRLAVVGYSESLSNLYTFEMRCRWLAFFLSGAFPLPSIREMEDDIRMWERYMKKYAGNGEFKRGCIAGIHIWYNDQLCKDIGCNPMRKNGFLAELFEPYGLADYDGITPPTWIT

>Achantus leucostachyus_TRINITY_DN3391_c0_g2_i5

MERRVGIIGAGISGLLACKYAVSVGLHPMVFEAQHHTGGLWNHTIQSTRLQNVKEFFQFSDFPWSSSVKDLFPTNTQVLEYVQSYAHHFQLLRYIKFDSRVISIDYVGESEEDMENWELWGGAGKAFGSKGKWILRVIHAEDQASIKEYVVEFVVVCIGRFSGLPDIPEFPPGRGPEAFSGKVLHSLDYSAMDNAAAAQFIKGKRIAIIGSGKSATDIAFECALANGSEKPCKVIQRTIHWMCPDAQPWGVSFGHLFFTRFTELMVHKPGEGFVTSAVATLLSPLRWVMSKFVESYLRWKLPLQKYGMIPEESFVQEASSCQISFLADNFYDKVVDGSIVFKKSEHFSFCKEGLVIDGESNPIEADVIIFATGYKGDEKLKNIIASPTFQKYILGSPNTIIPLYRQMVHPRIPQLAVIGYSESLSNLYTFEIRCKWLAFFLDQAFQFPSIKEMENEVKLWEKYMKKYSGNGKFRRGCIGGVHIYYNDQLCKDIVCNPRRKKGVFSELFEPYGLADYNGIVPGKASSLMPL

>Achantus ungaricus_TRINITY_DN4324_c0_g1_i27

MAKRVGIVGAGLSGLLACKYAVSVGLRPVVFEAQQRVGGLWNHTLESTRLQIDKAHYRFSDFPWPSSVEGDFPTNTQVVEYAVSYARHFQLLPYIKFGSRVTTIDYVGESHEEIESWESWGGSGEAFASKGKWILEVEEAGSVQEYEVEFVVLCIGRFSGLPDIPEFPPGHGPEVFAGKVLHSMDYSDMDNARAAEFIEGKRIAVIGSSKSAIDVAFECASANGSEKPCTMIQRTSHWMKGLFSSLLASVLSPLRWGVSKCIEMCLRWVLPLKKFGMIPNDSFVQEASSCQLLFLPEKFFNKVEDGSIVVKKSQFFSFCKEGLIIEGTEQPVEADIVVFATGYKGDEKLKNIFASPTFQSYIFGSPSSIVPLYRQMVHPRIPQLAVLGYSESLANLYMFEIRSKWLAFFLDQAFQLPSIQQMEEETKMWDRHMKKYAGNGMFRRNCIAGAHVWYNDQLCKDMGCNPRRKNGMLSELFEPYGPEDYTGIDPAQHLL

>Aphelandra aurantiaca_TRINITY_DN13240_c0_g1_i3

MEMKKTVAIVGAGISGLLACKYALSIGLNPVVFEAESCVGGLWNHTIECTRLQSIKDYFQFSDFPWPSTIKTMFPKNEQVLEYVNSYADHFQLRQYVTFNSKVMSIGYEGESKEEMGSWKSWGGTGMAFGSKGKWRLQVEAGGGMQEYHVADFVIVCIGKFSGLPAVPDFPPGRGPEVFPGKVLHSMEYSDMDNASAAQFIKGKRIAVIGSGKSAIDIAFECGRANGSDTPCTVIHRNAHWMLPYEKPWLLAFALLCFTRFSELLVHKPGEGFFASVLATLLSPLRWLVSKFLEISLRWRLPLKKYDMIPSENFLEEAHSCQVIMLQDNFFNKVVDGSIVFKKSEHFSFCKEGLIVEGKDSPVEVDIVILATGYKGDEKLKNIFASPTFQNHIFGSPNSTVPLFRQMIHPRIPQLAVVGYSESLSNLYTFEMRCRWLAFFLYRAFALPSIKEMEDDIKMWERYMKKYAGNGEFKRGCIAGIHIWYNDQLCKDMGCSPRRKKGILSELFQPYGLEDYDGITPVRIHPSYFAK

>Lamium album_Cluster-2496.6023

MEKRIAIIGAGISGLLACKYTASIGLNPMVFEEQDNVGGLWNHTIESTRLQQIKQSFQFSDFPWPSCADDNPKSAQLVEYLQSYAQNFDLLPYIKFNSRVMNIDYVGESKEEMQSWDLWGGSGKAFGSKGRWILKVVHTKDDDDSIKEYEAEFVVLCTGRFSGLPNIPEFAPGYGPEIFSGQVLHSMDYSAMENGSAAEFIKGKRIAIIGSGKSAYDIAFECANANGNDNPCTLIQRTINWTFPDAKPWAMNYNFLVLTRFSELMIHKPGEGFLSRALATFLTPLRWGISKSVESYLRWKLPLKKYGMVPKESFVAGASACQIFLLPRENFYDKVEDGSIVLKKSQQFRFCKQGLIIDGDEKNPINADIVIFATGYKGDEKLKNIFASPTFQNYIVGSPTSTIPLYRQMIQPRIPQVAIIGYSESFSNLFTFEMRCKWLAFFLDQAFELPSIGSMEREVESWETYMKMYAGKHKFRRACIGCVHVWYNDQLCRDIGCNPRRKKGFFSELFQPYGSADYVGMGTS

>Thunbergia alata_TRINITY_DN8382_c0_g1_i1

MYKTQIFQRVEINGGKPNQLLRINISLNTLESSFMEKRVAIIGAGISGLVACKHLASAGLRPLVFEAAAGIGGVWNRTMACTRLQSEKEFYCFSDFPWPSSVPDLFPKNTQILDYIHSYAHHFDLFQYIRFNSTVTRIEYVGEDMPLWPSSGKAFASNGKWILHVLQQNSTKEYVFEFVVLCIGRFSGLADIPKFPPGCGPEVFTGEVLHSMDYTAMDHTDAARLVKGKRVAIIGSGKSAIDLAFECANANGNEKPCTVIQRSIHWMFLDAHAWGVPFSFLCFTRLGELMLHKPGESFFSSLLATLLSPVRWGLSKFVESYLKWRFPLKKHNMIPKESLIQEVSSCQILFLPSENFYDKVEDGSIHLKRSNQIRFYKQGLIIDDTTKVEADLVVFATGFKGDEKLKNIFASPTFRNIISGSPDSIVPLYRQMIHPRIPQLAVIGYSESLANLYTSELRSKWLAFFLDEAFSLPSIKEMEEEIEEMEKFLKKYSGNGQFRRSCIASAQIWYNDQLCKDMGFNPNRKKGVLLNLFQPYGPSDYIDIDPAH

>Lamium galeobdolon_5154_HQ_transcript/262901

MEKRVAIIGAGISGLLACKYTASIGLKPIVFEEQDNVGGLWNHTTESTRLQQIKQSFQFSDFPWPSSADDNPNSAQLLEYLQSYARNFDLLPYIKFNSKVINIDYLGESKQEMQSWDLWGGSGKAFGSKGKWILKVLHTKDDSIKEYEAEFVVVCTGRFSGLPNIPEFAPGYGPEMFSGQVLHSMGYSAMDNASAAEFIKGKRIVIIGSGKSAFDIAFECANANGNDNPSTLIQRTVHWTFPDAQPWGVNYAFFVLTRFSELMVHKPGEGFFSSALATLLTPLRWGMSKFVESYLRWKLPLQKYGMVPKESFVAGASACQIFFLPDENFYDKVEDGSIVLKKSQQFRFCKHGLIIDGDDKNPINADIVIFATGYKGDEKLKNIFTSLTFQNYIAGSPTSTVPLYRQMIPPRIPQVAVIGYSESFSNLFTFEMRCKWLAFFLDQAFELPSIRGMEKEVEAWETYMKRYTGNNKFRRACIGCVHVWYNDQLCRDIGCNPRRKKGFFSELFEPYGSADYVGMHGN

>Lamium galeobdolon_5154_HQ_transcript/264384

MEKRVAIVGAGISGLLACKYAASKGFNPTVFEAQDNVGGLWNHTIESTRLQVGREVLQFSDFPWPSSADKIPHTSQLMEYLQSYAQNFDLLPYIKFNSKVMSIDYVGESEEEMQSWELWGGAAKAFGSKGKWTLKVFHTKEDSIKDYETEFVILCIGRFSGLPNIPQLSPECGPETFSGKVMHSMDYSAMKNSDAAQLIKGKRIAIIGSGKSATDIAFECANANGNDKPCTMIQRSVHWGLPDLYPLGIKFTYLFFTRFSELMVHKPGEGFLANVLATLLTPLGWLMSKLIESYLRWKLPLKKHGMIPKDSVARDAASSCRALFLPHENFYDKVEDGSIVLRKSQQFSFCKQGLIIDGDDKNPINVDIVMFATGYKGDEKLKNIFASPTFQNYIAGSPTSILPLYRQMIHPRIPQLAVIGYS

>Lamium galeobdolon_5154_HQ_transcript/174715

MEKRVAIIGAGISGLLACKYTASIGLKPIVFEEQDNVGGLWNHTTESTRLQQIKQSFQFSDFPWPSSADDNPNSAQLLEYLQSYAQNFDLLPYIKFNSKVINIDYVGESKQEMQSWDLWGGSGKAFGSKGKWILKVLHTKDDSIKEYEAEFVVVCTGRFSGLPNIPEFAPGYGPEIFSGQVLHSMGYSAMDNASAAEFIKGKRIVIIGSGKSAFDIAFECANANGNDNPCTLIQRTIHWTFPDAQPWGVNYAFFVLTRFSELMVHKPGEGFFSSALATLLTPLRWGMSKFVESYLRWKLPLQKYGMVPKESFVAGASACQIFFLPDENFYDKVEDGSIVLKKSQQFRFCKHGLIIDGDDKNPINSDIVIFATGYKGDEKLKNIFTSLTFQNYIAGSPTSTVPLYRQMIPPRIPQVAVIGYSESFSNLFTFEMRCKWLAFFLDQAFELPSIRGMEKEVEAWETYMKRYTGNNKFRRACIGCVHVWYNDQLCRDIGCNPRRKKGFFSELFEPYGSADYVGMHGN

>Camellia sinensis_XM_028211707

MENRVAIVGAGMSGLLACRYTLEKGFQPVVFEAQAGIGGLWAQTIESTKLQNDKEAYQFSDFPWPSSVQVFPSNTEVMDYFESYAQHFGIFPYIKFKSKVISIDYVGESDEELQRWDLWGGTGKPFGSKGKWHLIVEHTEKFSTEEYQFEFVILCIGRFSGLPNIPEFHPQYGPEVYTGKVMHSMDYSAMDNAKAAELIKGKRIAIIGSQKSAVDIAAECANANGVDYPCTMIQRSTQWMFPTGYIWGMNLAFLYFNRFAELLVHKPGETFLQSILASLLSPLRWGVSKFVESYLRWKLPLKKYGMIPKHSFLQGVSSCQILMLPENFYGKVEEGSIILMKSQSVGFCREGLIIDEEVEPLKTDLVILATGYKGDEKLKNMFHSPTFQNWIMGSPTSTVPLYRQIIHPRIPQLAIIGFSESFSNIYTSEIRCQWLAHFLNRTFELPSITAMEREVTMWENYMKRYAGGLYRRSCIGALHTWYNVQLCRDIGCKLRRKKGLFAELFEPHGPTDYVGLEPQ

>Ruellia brittoniana_AYIY_scaffold_2088915

MEKRVAVIGAGISGLLACKYAASKGFTPIVFEEQSQVGGLWNHTIEITRLQNVRYFFEFSDFPWPPSVQDLFPTSTQLLEYVQSYANHFQLLPYIKFNSRVISVDYVGESEEEMSTWGLWGGSGKAFGSKGKWNLTVVDINDQSVKEYVAEFLVQCIGRFSGLPFIPENFSPGEGPQVFSGKVLHSMDYSAMDNAAAAEFVRGKRVAIIGSGKSAVDLAFQCAMANGSDIPCTVIQRTIHWMFPDPEPWGVNFGYLCFTRFAELMVHKPGEGFFSSALATVLSPLRWAVSKFVESYLRWKLPLKKYDMIPKASFYDETSSCRILFLPENFYDKVEDGSIVFRKSEQFSFCKEGLMIEGQDEPIKADIVIYATGYKGDEKLRDIFASPTFQNYILGSPKSILPLYRQIIHPRIPQLAVIGYSESLSNLYTFEMRCKWLSFFLEQTFNLPSIKEMEDDIDKWDKYMKKYAGNGHFRRSCIVGVQIWYNDQLCKDIGCNPRRKKGFWSDLFEPYRLKDYDGITPHQI

>Verbena hastata_GCFE_scaffold_2056413

MEKRVAIIGAGISGLLACKYTASKGFNPVVFEAQDQVGGLWNHTLESTRLQNVKEFYQFSDFPWPSSVEDMFPKNTQLLEYLQSYAQHFELLPYIKFNSKVMSIDYVGESEEEMQSWVLWGGVGKAFGSKGKWNLRVLDTKEDLVKEYAAEFVVICSGRFSGLPDIPEFPSGFGPEIFSGKVLHSMDLSAMDNESAAAFIKGKKVAVIGSGKSAVDVAYECAKANGNNIPCTVVQRTVHWMLPDAQPWGVSFGFLCFSRFAELLVHKPGEGFLSSVLAKLFSPLRWMISKFVESYVRWKLPLKKYGMIPKESFVQEASSCQITFLLENFYDKVEDGSIVFRKSQKFIFCKEGLIIDGEENPIKADIVIFATGYKGDEKLKNIFASPTFQQCISGSPTSIIPLYRQMINPRIPQLAVIGYSESLSNLYTFEMRCKWLSFFLDQTFHLPSIKEMEEDITMWEEYMKKYAGNGKFRRSCIGGIPIWYNDQLCKDIGCNPRRKKGFFSDLFQTYGLADYDGISPNHHF

>Andrographis paniculata_XM_051257745

MEPKRVAIVGAGISGLLACKYAASVGLTPVVFEEQPHAGGLWNHTIESTRLQNSKDFFQFSDFPWPESVRDVFPTNAQLLHYLQSYARHFHLLPYINFNCRLLSLDYVGVSQEEMASWHLWGGSGQAFASEGNWNLKVLHIDDHSVKEYAAEFVVLCIGRFSGLPSIPVFPPGNGPEVFAGKVLHSMDYSAMDNAAAAEFIKGKRIAIVGSGKSAIDIAFECASANGSSNPCSVIQRTTHWTSPDAMPWGVNFGYLFFTRFSELLVHKPGEGFFSSALATLLSPLRWAISKFVESHIRWKLPLKKYGMIPKESFVREASSCQILFLPENFYDKVVDGSIVFRKSQHFSFCKEGLMIEGEVNPIKADVVIFATGYKGDEKLKNIFVSPTFQNYIFGSPKSSVPLYRQMIHPRIPQLAVIGYSESLSNLYTIEMWCKWLGFFLDQSFSLPSIKEMEDDLKKWDAYMKKYAGNGKFRRGCIGGVHIWYNDQLCKDIGCNPRRKKGFFSDLFEPYGLRDYNEIGPNQR

**Fig. 5 BX4 phylogeny**

>Gossypium hirsutum_CYP82D1

MDLLDFSTFGYAVVLGITLLFLYTKLKKSSSGSSSKAAPVAAGAWPIIGHLPLLGGPKTPHETLGDLGEKYGPAYMIRIGVHPALVVNSSEVAKEIFTVNDMYVSSRSEFAAAEHLGYNYAMFGFSPYGQYWREMRKITMLEVLSNHRIDQLKKVFVSEIEGSMKLLYKTWAAKKDGSSKVLVEMKKHFSDLTLNVIMRTVAGKRYSVVAEEDQKEVLRYRKALRDFFHLTGMFVLGDAVPFLRWLDIGGYEKWMKKTAKELDEISGGWLDDHRKGGRWDENKKEKDFMDVMNSVLKGASLAGYDADTINKATSLNMILAGSDTTTVTLIWGLSLMLNKPHILKKAQEELDTYIGRDRFVNETDIGKLVYIQAIVKETLRMYPPAPLSAPRELSESCSIGGYDIPKGTRLIINLHKIQRDPKKWPEPSEFKPERFLTTHKDVDVRGQHFELMPFGSGRRSCPGTSFALHMLYLTMSNFLHAFDFSTPSNGLIDLTGTVGLTNIKSTPLEALVSPRLAPELYN

>Arabidopsis thaliana_CYP82C2

MDTSLFSLFVPILVFVFIALFKKSKKPKHVKAPAPSGAWPIIGHLHLLSGKEQLLYRTLGKMADQYGPAMSLRLGSSETFVVSSFEVAKDCFTVNDKALASRPITAAAKHMGYDCAVFGFAPYSAFWREMRKIATLELLSNRRLQMLKHVRVSEISMVMQDLYSLWVKKGGSEPVMVDLKSWLEDMSLNMMVRMVAGKRYFGGGSLSPEDAEEARQCRKGVANFFHLVGIFTVSDAFPKLGWFDFQGHEKEMKQTGRELDVILERWIENHRQQRKVSGTKHNDSDFVDVMLSLAEQGKFSHLQHDAITSIKSTCLALILGGSETSPSTLTWAISLLLNNKDMLKKAQDEIDIHVGRDRNVEDSDIENLVYIQAIIKETLRLYPAGPLLGHREAIEDCTVAGYNVRRGTRMLVNVWKIQRDPRVYMEPNEFRPERFITGEAKEFDVRGQNFELMPFGSGRRSCPGSSLAMQVLHLGLARFLQSFDVKTVMDMPVDMTESPGLTIPKATPLEILISPRLKEGLYV

>Scutellaria baicalensis_CYP82D1

MELSSVIYGAIALLSLFYCYLHFSKPKKSSLNAPPEAGGARFITGHLHLMDGRSASDKLPHINLGLLADQHGPIFTIRLGVHRAVVVSSWELAKEIFTTHDTAVMARPRLIADDYLSYDGASLGFSPYGPYWREIRKLVTTELLSARRIELQRATRVREITQFTGELYKLWEEKKDGSGRVLVDMKQWLGNLSLNLVSRMVVGKRFYGGDDSETTKRWRGVMREFFQLIGQFIPGDGLPFLRWLDLGGFEKRTRDTAYELDKIIAMWLAEYRKREYSGDDKEQCFMALMLSLVQANPTLQLHYDADTIIKATCQVLISAASDTTTVILIWVISLLLNNADVLKKVQEELDEQVGRERRVEESDISNLPYLQAVVKETMRLYPPAPFAGVRAFSEDCTVGGYHIQKGTFLIVNLWKLHRDPRVWSDDALEFKPQRFFDKKVEVKGQDFELMPFGGGRRMCPGSNLGMHMVHFVLANILQAFDITTGSTVDMTESVGLTNMKATPLDAILTPRLSPTLY

>Scutellaria baicalensis_CYP82D2

MLYDNSQYLAMEFSSAIYGAIAFFLFLYYCLLYTTSSKPNTRAYKAPPEAGGARLFSGHLHLMAGGTTGELPHINLANLADKHGPVFTIRLGVKRALVVSSWESAKELFTTCDVAVSSRPRMKAAKLLGYDFAMFGFAPYGAYWRELRKLISVELLSTRRMELQKEVRDSETRESIKELYKLWEERSEGSDSVLVDMKQWFGDLNLNVVLRMVAGKRWIGPETGRWREVLRDFFYLAGMFVPADAFPFLGWLDLGGHEKRMRQTAKELDGIVGGWVAEHREKEYSGEDKPKDFVDVMLSVVQGSSLRADYDADTIIKATCEALIVGGSDTTTVMLIWTLSFLLNNRHVLRKAQEELDKHVGRERRVNQSDINNLVYLQAIVKETLRLYPAGPIGGIREFTQDCQVGGYHVPKGTWLIVNLWKLHRDPKVWSEDCLEFRPERFLNKNIDVRGQDFELIPFGGGRRICPGANFGLHMLHLVLANLLQAFELTTVSDQVIDMTESAGMTNMKATPLNVLVAPRLSPTLY

>Arabidopsis thaliana_CYP82G1

MTFLFSTLQLSLFSLALVIFGYIFLRKQLSRCEVDSSTIPEPLGALPLFGHLHLLRGKKLLCKKLAAMSQKHGPIFSLKLGFYRLVVASDPKTVKDCFTTNDLATATRPNIAFGRYVGYNNASLTLAPYGDYWRELRKIVTVHLFSNHSIEMLGHIRSSEVNTLIKHLYKGNGGTSIVKIDMLFEFLTFNIILRKMVGKRIGFGEVNSDEWRYKEALKHCEYLAVIPMIGDVIPWLGWLDFAKNSQMKRLFKELDSVNTKWLHEHLKKRSRNEKDQERTIMDLLLDILPEDIVISGHVRDVIVKATILALTLTGSDSTSITLTWAVSLLLNNPAALEAAQEEIDNSVGKGRWIEESDIQNLKYLQAIVKETHRLYPPAPLTGIREAREDCFVGGYRVEKGTRLLVNIWKLHRDPKIWPDPKTFKPERFMEDKSQCEKSNFEYIPFGSGRRSCPGVNLGLRVVHFVLARLLQGFELHKVSDEPLDMAEGPGLALPKINPVEVVVMPRLDPKLYSLL

>Rauvolfia serpentina_KY926696

MDLLQILLAIAGLLAILLLQKQWRTKTSPGAKAGRKLPPEPAGAWPVIGHLHKLGGPNPIYRNLAEWSDKYGPVMTLKLGMQNAVVVSDREAIKECFTTNDKALADRPPSSIGLHLGFNYAAIGAAPYGPYWRDMRKLVLLEVLSSRRLEMLRNVRISEIGTSIKELYSNIIRSSGGSGPAKVVISHWIEQLTLNYILRTIAGRRFSDDSSKDAQYVKGVINDFMYFAGQFVVSDVIPIPLLRWLDPQGHLKGMKRVAKEVDTMCEAWIQEHVQRRMREKPGPGQEQDFIDVLLNNRDVMRKAQEEIDNHVGKERWVDETDLKHLVYLQAIVKEGLRLYPPGPLGAPHRAIEDCQVGGYFIPKGTQLLVNVWKLHRDPRVWSEPEKFMPERFLTRQAEVDVFGHHFELLPFGSGRRACPGITFAVQVMHLTVARLLQGFDMTTPSNLPVDMTEGPGVTMPKAHPVEVLMMPRLPSALYEP

>Arabidopsis thaliana_CYP82C4

MDTSLFSLFVPILVFVFIALFKKSKKPKYVKAPAPSGAWPIIGHLHLLGGKEQLLYRTLGKMADHYGPAMSLQLGSNEAFVVSSFEVAKDCFTVNDKALASRPMTAAAKHMGYNFAVFGFAPYSAFWREMRKIATIELLSNRRLQMLKHVRVSEITMGVKDLYSLWFKNGGTKPVMVDLKSWLEDMTLNMIVRMVAGKRYFGGGGSVSSEDTEEAMQCKKAIAKFFHLIGIFTVSDAFPTLSFFDLQGHEKEMKQTGSELDVILERWIENHRQQRKFSGTKENDSDFIDVMMSLAEQGKLSHLQYDANTSIKSTCLALILGGSDTSASTLTWAISLLLNNKEMLKKAQDEIDIHVGRDRNVEDSDIENLVYLQAIIKETLRLYPAGPLLGPREAMEDCTVAGYYVPCGTRLIVNVWKIQRDPKVYMEPNEFRPERFITGEAKEFDVRGQNFELMPFGSGRRSCPGSSLAMQVLHLGLARFLHSFDVKTVMDMPVDMSENPGLTIPKATPLEVLISPRIKEELFV

>Aphelandra squarrosa_BX4

MAKVCNQILHTGSSFSMGMQMEGLLWWVLAVALAYIVASFFFNNGKKPKLPPGPKPWPIIGNLNLIGPIPHQSLHDLFQKYGELMHLKFGHFPVVVASTPEMAKQFLKTHDTAFASRPALDAGRLTSYNYTDLTWAPYGPYWRLARKIYVVDVFSAKKLDSDAFHQIRVEERQTFFSLMHSLAGKPVVLRGHLGRAAVITISRMVMGKAYFGEDESCLIGTDGFQELLDEWFFLNGVFSICDWIPWLRFIDPQGYVRRMKNLHKELDRIFDFVVADHEATRMKDTDSCSEKDVVYKMLDMTEEPTLGVALSREHVKALLQNLILGGTDTTPTSVEWILCELMKNPPIVRKAKEELNRAVGRDRWVEESDFPELPYLDAIIKETMRLHPIATFLAPHCAMEDCKVAGYDIPKGATVFINSWSLGRDPDSWDRPLEFLPERFMEKEIDIMGSNFAMLPFGAGRRRCPGYGHALKNIRAIVANLLHGFELKLPNGMKPSDVNMEEEYGLTTHPKEPLALIMEPTLPPHLY

>Pisum sativum_CYP82A6

MDFVLNYLNTTTIAFISLISLLFFLFRFSKVSHTKEPPIVSGSWPLLGHLPLMRNTQTPHKTLGALVDKYGPIFTIKLGATNALVLSNWELAKECFTKNDIVVSSRPKPVAVELMSYNQAFIGWAPYGTYWRQLRKIVTLEILSNRRIELLSHIRVSEVQTSIKELVNVWSNQMSSQYGLLDDTKSSSTNDYASVELKKWFAQLTLNMVLRMVVGKRCFGDVDVENKEEAKKFLENIRDFMRLIGTFTVGDGVPFLKWLDLGGHEKEMKKCAKKFDEMLNEWLEEHREKKGLGSEDKVVGERDFMDAMLLVLKDKPIEGFDVDTIIKATTLELILGGSDTTAGTLTWAMCLLLKHPHVLEKLKEELNTYIGKERCVKESDINKLVYLHAIIKETLRLYPPAPFSSPREFTEDCTIGGYHIKKGTRLMPNLWKIHRDPNVWPDPLEFKPERFLSTHKDVDVRGQNFELLPFGSGRRMCAGMSLGLHMVHYILANFLHSFEILNPSPESIDVTEVLEFVTTKATPLEVLVKPCLSFKCYESM

>Eschscholzia californica_CYP82B1

MEKPILLQLQAGILGLLALICFLYYVIKVSLSTRNCNQLVKHPPEAAGSWPIVGHLPQLVGSGKPLFRVLGDMADKFGPIFMVRFGVYPTLVVSTWEMAKECFTSNDKFLASRPPSAASSYMTYDHAMFGFSFYGPYWREIRKISTLHLLSHRRLELLKHVPHTEIHNFIKGLFGIWKDHQKQQQPTGREDRDSVMLEMSQLFGYLTLNVVLSLVVGKRVCNYHADGHLDDGEEAGQGQKLHQTITDFFKLSGVSVASDALPLLGLFDLGGKKESMKRVAKEMDFFAERWLQDKKLSLSLSSETNNKQNDAGEGDGDDFMDVLMSILPDDDDSLFTKYSRDTVIKATSLSMVVAASDTTSVSLTWALSLLLNNIQVLRKAQDELDTKVGRDRHVEEKDIDNLVYLQAIVKETLRMYPAGPLSVPHEAIEDCNVGGYHIKTGTRLLVNIWKLQRDPRVWSNPSEFRPERFLDNQSNGTLLDFRGQHFEYIPFGSGRRMCPGVNFATLILHMTLARLLQAFDLSTPSSSPVDMTEGSGLTMPKVTPLKVLLTPRLPLPLYDY

>Nicotiana tabacum_CYP82E1

MYHLLSPIEAIVGLVTFAFLLYLLWTKKQSKILNPLPPKIPGGWPVIGHLFYFNNNGDDDRHFSQKLGDLADKYGPVFTFRLGFRRFLAVSSYEAMKECFSTNDIHFADRPALLYGEYLCYNNAMLAVAKYGPYWKKNRKLVNQELLSVSRLEKFKHVRFSIVQKNIKQLYNCDSPMVKINLSDWIDKLTFDIILKMVVGKTYNNGHGEILKAAFQKFMVQAMEIELYDVFHIPFFKWLDLTGNIKAMKQTFKDIDNIIQGWLDEHIKKRETKDVGGENEQDFIDVLLSKRSNEHLGDGYSHDTTIKATVFTLVLDATDTLALHIKWVMALMINNKNVMKKAQEEMDTIVGRDRWVEENDIKNLVYLQAIVKEVLRLHPPAPLSVQHLSVKDCVVNGYHIPKGTALLTNIMKLQRDPQIWVDPDTFDPERFLTTNAAIDYRGQHYELIPFGSGRRACPAMNYSLQVEHLSIAHLIQGFNFATTTNEPLDMKQGVGLTLPKKTDVEVLITPRLPPTLYQY

>Consolida orientalis _BX4

MYFHVTHSHSVFTPSLSVHSSTMESLNQSYPVIICFWSFALLLLLYTMPISWKRRRDMAPEPSGAWPLIGHLPLLMGHTLPQKTLGALADKYGPVYTIRLGLRKVLVINSWEAAKECFTTNDKAFAHRPSSVALRIMGYNNAILFYASYGPYWREVRRIAILELLSNRRIELIKHIQVSEVDVSMRELYKMWQAGCTDGGYSLVEMKQWFIDINLNMIVRMVVGERYFGTRMDDDGIRFQKAVHSFFHLVGLFVLSDAVPFLGWLDFRKKEMKRTAKELDRIMQKWLDEHKQKKLAGGGKGEQDFMDMMLSVLEDKKLPDFDADTINKAVSMNMIFGGVDTITVGLTWTFSLLINNPRVLKKAQDEIEATVGKHRPVGALDVEKLVYLQAIVKESLRLYPPSPMPPQHLTVEDCTVAGYHVPAGTCLVANIWKIQRDPRVWSDPCEFQPERFLGDGANVDLRGKNFEFIPFGAGRRQCPGISYALQVLNLVLARLLQGFDFKTKSNQALDMTESPGLTNMKATPIHVFITPRLPSDLY

>Nelumbo nucifera_XM_010266410

MDSLLQSPTNIGALLAVLVFIYYLSSLISKNIEVKKRKMAPEAAGAWPVIGHLPLLGGPELPHITLGAMADKYGPAFTIRLGVRRALVVSDWEVAKECFTINDRALATRPSGVAMKHMGYNYAMFAFAPYGPYWREVRKIATLELLSNRRLEMLKHVRVSEVNMSVRELYGLWLKNKNATGADSVKVEMKRWFGDLTLNNVLRMVAGKRYFGATAACGSAEAVRCQTVMRSFFRLVGQFVIADAIPFLGWLDMQGHEKAMKRTAKELDSILEGWMEEHRKMRLSNDEANGEQDFMDVMLSILKDSKLSGYDADTINKATCMNLILGGNDTIKVCLTWALSLLVNNQEVLKRVQDELDRHVGKDRNVEESDMKKLEYLQAVVKEALRLYPPAPLSGPHEAMEECTVAGYHVPVGTRILVNLSKIQRDPRVWSDPLEFRPERFLTSHVGVDVLGQHFELIPFGSGRRACPGTSLGLKVVHLALARLLHAFHLHTPSGAPVDMTESFGLTNLKATPLEVLLLPRLPSHLYQY

>Alnus glutinosa_XM_062302999

MDFLSPYPNSAIAGLLGVILLSYYLVRRSRVGSAKIAPVAAGAWPIIGHLPLLGGTEPPHITLGAMAEKYGPVFTIQLGLQPALVISCWEMAKECFTTNDLAVSSRPKLVAAKHFGYNFAMFGFAPYGPYWRELRKIAALELLSNHRLELLSYVRVSEVETTLQELYILWTKKKNVSGQILVELKQWFGDMSLNVILRMVAGKRYFSSSAVADEESRRCQKSVRAFFHYLGMFVVSDAIPYLGWLDLGGHEKAMKKTAKEFDGFLAGWLEEHKRKRALGEAKGERDFMDVMLSVLDDKDVAGYDADTINKATCMNIIAGGNDTSTVTLTWAISLLLNNRRVLKKAQDELDVQVGKERAVKESDISQLVYLQAIVKEIFRLYPPAPLSGPREFCEDCIIGGYHVPKGTRLITNLWKIHIDPRIWSDPLTFQPERFLTTQKDIDIRGKNFELIPFGSGRRVCPGISFGLQMIHLALASFLQMYEISTPSNALVDMTESSGLTNLKATPLEVLITPRLPSKLYGLTTV

>Macadamia integrifolia_XM_042638198

MDFLLEFPTVVFFFILVSLYYLLPWRSRNTKLSNSNKRVAPEAVGAWPIIGHLHLLGGNSIPHITLGALADKYGPAFTIRLGMQPALVVSSWEVAKECFSTNDRALATRSKSIATKLMGYNYALFAFTYYGTYWREIRKIVMLQLLSNRRLEMLKHVRASEIEAFIKEIYKMWEEKKGGQNIQVLVDMKKWFADLTLNIIVRMVAGKRYFSNKASDAEEALQCQEGVRDFFHYIGLFVVSDSFPFLEGLDLQGYEKAMKKTAKNLDSIVEGWLKEHKLKRSSANYGQAKGDQDFMDVVISIMEDSKLSSEEYDADTIIKATCLNMILGANDTTVVTFTWTLSLLLNNPHVLKRAQDELDIHVGRDRQVDESDIVKLEYLQAIIKETLRLYPAAPLSVPHEAIEKCTIAGYHVPIGTRIVTNLYKIHRDPRVWKDPDEYRPERFLTSHVDLDLRGQHFEFIPFGSGRRMCPGISFALQVLHLGLARLLHGFDFSTPSNALVDMTESPGLTNIKATPLEVLLTPRLPPEVYG

>Papaver somniferum_XM_026598259

MANLDLLNQVSKPTLFYGTASIALLVYLIIKRFSSNKKSSSKLQQAPEPAGAWPIIGHLPLLAGPDLPHITLGKLADKYGPAFVIRIGVHKALVINSWEVAKECFTTNDKVFSSRPRQVAMKHMGYDYAMFGFAPYGDYWRELRKIINREVLSHSRIESLYHIWGKEINTSIKELYGLCGKKPALVEMKQWFSDMTLNMSVMMVAGKRYNFGADKADDEAKRCQDGLRNFFRLVGLFVPSDAVPSLAWLDIGGYEKEMKKVAKELDELMQEWLDEHKKKRALLKAQGKPRGDQDFMDVMMTILENEKLSEFDSDTVNKATGLTLILGGTDTNMVNSVWALALLVNHQDALKKAHDELDLHVGRDRQVSESDVKNLVYIQAVLKETLRLYSGPLSGLRESTEDCTVAGYHVPAGTRLIINASKIHRDPRVWSDPTSFNPERFLEEHKGMDVRGQDFELLPFGAGRRICPGTAFALQVLPLALARLLHGFDFKRPTVAPIDMTESPGLTNAKSTPLEVLVSPRLSPELYEC

>Populus trichocarpa_XM_024584927

MDPSPQLIAIALFFSCILLYNALIKKNSIKGNQIKEAPEPAGAWPIIGHLHLLGGGDQLLYRTLGAMADKHGSAFTIRLGSRRAFVVSSWEVVKECFTINDKALASRPTTVAAKHMGYNYAVFGFAPYSSFWREMRKIATLELLSNRRLEMLKHVRASEVDIGIRELYNSWANNSSSPVVVELKQWLEDLTLNVVVRMVAGKRYFGSAAASDDGEARRCQKAINQFFRLIGIFVVSDALPFLGWLDLQGHERAMKNTAKELDAILEGWLDEHRQRRVSAGIKDEGEQDFIDVMLSLKEEGQLSNFQYDANTSIKSTCLALILGGSDTTAGTLTWAISLLLNNRHMLKKAQEELDLHVGKERQVEDSDVKNLVYLQAIIKETLRLYPAGPLLGPREAMEDCKVAGYHVPAGTRLIVNVWKIQRDPRVWTNPSAFLPERFLTSHGDVDVRGQQFELIPFGSGRRSCPGVSFALQVLHLTLARLLHSFELATPMDQPVDLTESSGLTIPKATPLEVILTPRLPPKLYGY

>Gossypium hirsutum_XM_016812277

MNSLSLRYTTMDPFFQLTAVTIFLSIVFYKVLLGREESNAGSSKRMAPEPEGAWPILGHLHLLGGSDQLLYRTLGAMADKHGPAFTIRLGARRAFVVSSWEVVKECFTINDKALASRPTTVAAKHMGYNYAVFGFAPYTPFWREMRKIATLELLSNRRLEMLKHVRISEVEMGIRELYNLCYHNGSIPILVELKQWFEDLTLNVVVRMVAGKRYFGDSAVCDDGEARRCQKAISQFFHLIGIFVVSDALPFLWWLDMQGHEKAMKKTAKELDDLLEGWLKEHRQRRISGGIKAEGDQDFIDVMLSLQEEGGLSNFQYDADTSIKSTCLALILGGSDTTAGTLTWAISLLLNNREMLRKAQDELDLHVGKERPVDESDXKNLVYLQAIIKETLRLYPAGPLLGPREAMNDCTVAGYHIPAGTRLVVNVWKIQRDPRVWPNPSAFAPERFLTSHADIDVRGQQFELIPFGSGRRSCPGASFALQVLHLTLARFLHAFELSTLLDQPVDMTESPGLTIPKATPLDVLLKPRLPTKVYAS

>Citrus junos_OQ126882

MDLLLPLSSIIFVFSLLLFLYNASLRGRKSSNVRKAQKAPEPAGAWPIIGHLHLLGGGDQLLYRTLGAMADKWGPAFNIRLGSRSAFVVSSWEVVKECFTINDKALASRPKTAAAKHMGYDYAVFGFAPYTPFWREMRKIATLELLSNRRLEALKHVRISEVDMGLRDLYGLWAKNDSRPVLVELKKWLEDMTLNMVVRMVAGKRYFGASATCDDSEARQCQKAINQFFHLIGIFTVSDALPFFWWLDWQGHEMAMKKTAKELDAILEGWLEEHRQRRVSGGIESESEQDFIDVMLSLQEEGQLSQFQHSADISIKSTCLALILGGSDTTSGTLTWAISLLLNNPDMLKKAQEELDQQIGMDRQVDDSDIKNLVYLQAIIKETLRLYPAGPLLGPREAMEDCTVAGYHIPAGTRLIVNVWKIQRDPRVWKNPSAFQPERFLTSHTDVDFRGQQFELIPFGSGRRSCPGASFALQVLHLTLARLLHAFELATPSDQPVDMTESPGLTIPKATPLEVVLSPRLPPNPYGF

>Arachis stenosperma_XM_057873675

MNISFQLTIVAILLSFICYAFLSKRKNKESGKEAPVPNGAWPIIGHLHLLGSHHQLLYRTLGRMADQYGPAFNIWLGSRRAFVVSSWEVAKECFTTNDKALASRPTTVAAKHMGYNYAVFGFAPYSAFWREMRKIATLELLSNRRLEMLKHVRISELNMGIRDLYNSWVKNNSNPVVVELNRWLEDLSLNTVVRMVAGKRYFGASAKCDDDEARRCQKAINQFFHLIGIFVVSDAVPFLRWLDVQGHEKAMKKTAKELDEILEGWLQEHRKRRVHGEEDDRDFIDVMLSLQKRGHLSNFQYDSDTSIKSTCLALILGGSDTTAGTLTWSISLLLNNPDALRKAQEELDLHVGKERQVEDSDIRNLRYIEAIIKETLRLYPAGPLLGPREAQEDCTVAGYHVPAGTRLVVNLWKIHRDPRVWKDPCSFQPERFLTSHHAAIDVRGQNFELIPFGSGRRSCPGMSFALQVLHLTLARLLHAFDFATISNEPVDMTESPGLTIPKATPLEILITPRLQPQLYA

>Consolida orientalis_TRINITY_DN5568_c2_g1_i5

MESLDLKSPNQACIATTSIGAFMLFVLLYNVLWKKKASGKIAPAPAGAWPLVGHLPLLLEPNLPHKVFGALADKYGPVFHIRLGLHRALVINNWEAAKECFTTNDKALATRPSSVAIKIMGYDYAMFGFTFYGPYWREVRRIAILELLSNNRLNMLKDVRISEVDMSVRELYEFWEVNCKHDKYAMVEIKGWFSDLTLNVITRMVGGKRYFGSSSKLDDSTDEGKRLQKAIKDFFHLVGLFVLSDSVPFLGWLDFRGYEKAMKRTAKEADIIMERWLEEHKQKKLSSAGVKADGDFMDVMLSVLEDNKLPGYDADTVNKATCWNMILGGADTTTVTMTWALSLLLNNRHTLKKAQDEIDANVGKDRKVNNVDLDKLVYLQAIVKETLRLYPATPLLAPHEAIEDCTIAGYHVSAGTRLIPNVWKIQRDPRLWPNPDEFQPERFLTSHANIDFRGQHFGLIPFGSGRRSCPGMSFGIQVIHLVFARLLQGFNFETPSGEAVDMTESAGLTNLKPTPLQVLITPRLSSNRY

>Consolida orientalis_TRINITY_DN16314_c0_g2_i11

MESVQKLLHTTGTPVVALVVLLVTIIILFVWTRRSKSSKGKQAPELPGAWPIIGHLPFLASGKLLHRAFGELADKYGPIFSIRIGAHKALVVSSWEVAKECYTTNDKLFGSRPNFVAAEIIGHNYANFAFSPYGPYWVELRKITMSELLSHRRLDLLKHVRVSEIDASMKELYKMCVDGKGTAEVEMKKWYGDLNLNVILRMIAGKRYSSTTSAADESEAERWRTAMRDFMRLMGAFVPEDSLPSLRWFDLQGYKKEMRHTINELDSLFQGWLQEHKKKKLAGEIKSDSDFMYVMMTSLADAKMTYHDNDTIIKATCLSIISGGTDTTMLSLTWMLCLLLNNRRVLKKVQEELDNVVGRDRRVEESDIKNLVYLQAVVKETLRLYPAAPLSGPRQAADDCTVAGYHVPAGTRLMVNLWKLQRDPSVWSEPNEFRPERFLTDHPDMDVKGQDYELIPFATGRRMCPGIAFALQMMPLVTAHVLHGFELTTPNDEPVDMAETGGTTNDKVMPMEVVITPRIRPELYGL

>Amborella trichopoda_XM_020674533

MKSHTHMPSQQAQQTSMDYLLLLQPILAIFFALFLHQIWLKIKKSKNTYAKEAPEPGRAIPVIGHLHLLSGSKLLHHKLAEMADVYGPAFTLRLGRRRTLVISNWELAKECFTTNDKNFANRPRDSAAKLLGYDLAMIGFAPYGPYWRETRRIATLQLLSNRRLESLKHVRSSEVDASIREIYELWKEKERRAVEVNIQQKLLDLTFSTVVRMVAGKRFLRDGEDNEEGKRFRELIEEQFYLAGTSVVSDAVPILEWVDFQGTLKSMRRVAKEVGEIVEGWVSEHRRKRATGETEAAQDFIDVLLSLVEDGSYSSSIDPAIFIKANVVNLVAAATDTTAITMTWALSLLLNNRHALRRAQAELDLHVGKDRNVDESDVKNLTFLDAIVKETLRLYPAGPLLVPHEAIQDCHVGGFNVYAGTRLVVNAWKLQRDPRVWSDPERFDPDRFLTSHVDIDVWGKNYEFIPFGSGRRSCPGISFALHLLSLTLARFLHAFEWDTPFGECVDMTEGFGLTLAKATPLRVVLTPRLPSHLYQ

>Arabidopsis thaliana_Q9SZ46

MDTSLFSLFVPILVFVFIALFKKSKKPKYVKAPAPSGAWPIIGHLHLLGGKEQLLYRTLGKMADHYGPAMSLQLGSNEAFVVSSFEVAKDCFTVNDKALASRPMTAAAKHMGYNFAVFGFAPYSAFWREMRKIATIELLSNRRLQMLKHVRVSEITMGVKDLYSLWFKNGGTKPVMVDLKSWLEDMTLNMIVRMVAGKRYFGGGGSVSSEDTEEAMQCKKAIAKFFHLIGIFTVSDAFPTLSFFDLQGHEKEMKQTGSELDVILERWIENHRQQRKFSGTKENDSDFIDVMMSLAEQGKLSHLQYDANTSIKSTCLALILGGSDTSASTLTWAISLLLNNKEMLKKAQDEIDIHVGRDRNVEDSDIENLVYLQAIIKETLRLYPAGPLLGPREAMEDCTVAGYYVPCGTRLIVNVWKIQRDPKVYMEPNEFRPERFITGEAKEFDVRGQNFELMPFGSGRRSCPGSSLAMQVLHLGLARFLHSFDVKTVMDMPVDMSENPGLTIPKATPLEVLISPRIKEELFV

>Papaver somniferum_CYP82N4

MDLLLQYLQPISVALVVIALVWNYGRRNPTKKLAPEASGGRPIMGHLHLFNDGELTHRKLGAMADTYGPVFNIRFGSHKTLVVSDWEIVKECFTTNDKLFSNRPGTLGIKLMFYDADSVGYAPYGAYWRDLRKISTLKLLSNHRIDTIKHLRSSEVESCFESLYSQWGNGEKSGEFAPVRMDSWLGDLTFNVVARIVAGKKNFSANGDVGAQRYKAAMDEAMRLMRFFAFSDVIPSLSWLDNLRGLVRDMKKCASEIDSIMATWVEEHRVKRNSGGDSQLEHDFIDVCLDIMEHSSLPGDDPDLVVKSTCLDMILGGSDTTTVTLTWAMSLLLNHPQVLQKAKEELETQVGKNRQVDDSDIPNLPFIQAIIKETMRLYPAGPLIERRTMEDCEVAGYQVPAGTRLLVNVWKMQRDGNVYKGDPLEFRPDRFLTSNADVDLKGQHYELIPFGAGRRICPGVSFAVQLMHLVLARLLHEFEITTVEPETKVDMAESGGLLCYKIMPLEVLIKPRLEI

>Nicotiana tabacum_CYP82E4

MLSPIEAIVGLVTFTFLFFFLWTKKSQKPSKPLPPKIPGGWPVIGHLFHFNDDGDDRPLARKLGDLADKYGPVFTFRLGLPLVLVVSSYEAVKDCFSTNDAIFSNRPAFLYGDYLGYNNAMLFLANYGPYWRKNRKLVIQEVLSASRLEKFKHVRFARIQASIKNLYTRIDGNSSTINLTDWLEELNFGLIVKMIAGKNYESGKGDEQVERFKKAFKDFMILSMEFVLWDAFPIPLFKWVDFQGHVKAMKRTFKDIDSVFQNWLEEHINKREKMEVNAEGNEQDFIDVVLSKMSNEYLGEGYSRDTVIKATVFSLVLDAADTVALHINWGMALLINNQKALTKAQEEIDTKVGKDRWVEESDIKDLVYLQAIVKEVLRLYPPGPLLVPHENVEDCVVSGYHIPKGTRLFANVMKLQCDPKLWSDPDTFDPERFIAIDIDFRGQYYKYIPFGSGRRSCPGMTYALQVEHLTMAHLIQGFNYRTPNDEPLDMKEGAGITIRKVNPVELIIAPRLAPELY

**SI Fig. 9, BX5 phylogeny**

>Lamium maculatum_14367-like

MEVINFTTLVLFSSVIFLLVKAWRKPPNPNPQNLPPSPPSLPVIGHLHLLGGGAVPALARLKKKYGPIISLKLGEVTAVVISSREATKEALKIHDPACADRPDSTALEIMFYSYGDIAFCPYNEYWRQMRKICILEMLSAKNVKSYGYIRAEEIDSLVESLRLSSGEAVNITENISSLTFAVTCRASFGRVLQGHAKLIALLKELSTMAGGFEVADLFPSLKFLHPFSLNKYRLLRMRREMDAILDPAVEEHKLKKSGEFEGEDFVDVLLRMQKNKELQFPITTDNIKSVILDMFAGGIETTATTTDWAMAELMRNPRVMAKLQSEIREVLKGKTTVENRDVQGLKYLKLVVKESLRLHPPIPVLPRKCRQECRVGGFTIPNKAKVMIDVHSLGRDPQYWNDPETFLPERFENSSLDFLGSEYEFLPFGAGKRNCPGLNFGIANVEFTLAQLLYHFDWKLPQGMSLADVDMTEIEGLAVLRKNPLMVIPTPYEQPL

>Lamium galeobdolon_14367-like

MEVIDFTTLLLFSSLIFLLIKAWRKPQNPNPQNLPPSPPSLPVIGHLHLLGGGTVPALARIRKKYGPIVSLKLGEVTAVVISSRETVKEALKIHDPACADRPDSTALEIMFYSYGDIAFCPYNEYWRQMRKICILEMLSAKNVKSFGYIRVEEIDSLVESLRLSSGEAVNITEKISSLTFAVTCRASFGRVLKGHGKLIALLKELSTMAAGFEVADLFPSLKFLHPFSLNKYRLLRMRREMDDILDPAVEEHKLKRSGEFEGEDFVDVLLRVQKNKELQFPITTDNIKSVILDMFAGGIETTATTTDWAMAELMRNPRVMAKVQSEIREALKGKTTVENKDVQNLKYLKLVVKESLRLHPPIPLLPRKCRQECIVGGFTIPNKAKVMIDVHSLGRDPQYWNDPETFLPERFENSSLDFLGSDYEFLPFGAGKRNCPGLNFGIANVEFPLAQLLYHFDWKLPQGMSHADVDMTEIEGLAVLRKNPLMVIPTPYNPSL

>Lamium orvala_14367-like

MEVINFTTLLLFSSLIFLLIKAWRKPQNPNPQNLPPSPPSLPVIGHLHLLGGGAVPALARLKKKYGPIISLKLGEVTAVVISSREATKEALKIHDPACADRPDSTALEIMFYSYGDIAFCPYNEYWRQMRKICILEMLSAKNVKSYGYIRAEEIDSLVESLRLSSGEAVNITEKISSLTFAITCRASFGRVLKGHAKLIALLKELSTMAGGFEVADLFPSLTFLHPFSLNKYRLLRMRREMDAILDPAVEEHKLKKSGEFEGEDFVDVLLRVQKNKELQFPITTDNIKSIILDMFAGGIETTATTTDWAMAELMRNPRVMTKVQSEIREALKGKTTVENKDVQNLKYLKLVVKESLRLHPPIPLLPRKCRQECKVGAFTIPNKAKVMIDVHSLGRDPQYWNDPETFLPERFEESSLDFLGSNYEFLPFGAGKRNCPGINFGIANVEFPLAQLLYHFDWKLPQGMSHADVDMTEIEGLAVLRKNPLMVIPTPYEQPL

>Lamium galeobdolon_Bx5

MEVIDFTTLLAFSSLIFLLIKAWRNPNPQNLPPSPPSLPVIGHLHLLGGGAIQALARIRKKYGPIVSLKLGEVTTVVISSREATKEALKIHDPACADRPDSTAVKIMFYGCEDISFGPYNENWRQLRKICIMEMLSPKNVKSFGYIREEEIDSLLESLRLSSGEAVNLTEKISTLTFAVTCRASFGRVLKGHGTVVALLKELSPMSTGFQVADLFPSMKFLHPFSLNKYRAQQMRREMDDILDTAVEEQKLKKSGEFEAENFVDVLLRVQKNKELKFPITTDNIKSVILDIFAGGIETTATTNDWAMAELMRNPRVMAKVQSEIREALKGKTTVEEKDIQSLKYLKLVVKESLRLHPPVPLLPRKCRQECRVGGFTIPNKAKVLINVHSLGRDPLYWNDPETFLPERFENSSLDFLGNDCEFLPFGAGKRNCPGLNFGIANVEFPLAQLLYHFDWKLPQGMSHADVDMTEAEGMALLRKSPLMVIPTPYKQPL

>Lamium galeobdolon_TRINITY_DN4514_c0_g1_i3

MDIFHQIPLNFTTAVLIASFVFLLIKAITKKSPQNLPPSPPSLPVIGHLHHLAGGLLHEGLDKLSKTYGPIISLNLGQVRAVAISSPELAKEVTKVLDPACADRPESIALRTMFYENTDIAFCAYNDYWRQMRKICILELLSAKNVKSFGYIRQDETNTLIESLKFVSGKTVNLTEKVFVFSCTVTCRATFGNVLGDRDKFIVLVKKLAHMAGGMELADLFPSIKFLTVFTWNRFQLWKMKRGMDAILDPILAEHKLKRSGEGGSEDIVDVLLRVKKSGQLQFPITNDNVKAVILDMFAAGTESSATTIDWAMAELMRDPRVMTRVQSEIRETLKGKTTVEESDVQGLTYLKLVIKETLRLHPPFPLLPRQCKDEFKVDGYTIPVKTKVMVNVWGIARDPKYWEDAESFKPERFENSSIDFLGSNYEFLPFGAGRRNCPGLSFGIANLELPLAQLLFHFDWKLPQGMNPSDVDMTGVEGLAVGRKTPLVLIPTIHNPSN

>Lamium album_Cluster-2496.9342

MEIISLTTLLLLSTFIFFLIKTIKKPKPSENEGKLPPSPPKLPLIGHLHHLTGDLPHHALSKISKKYGDIVSLKLGETNAVIVSSRELAKEAMKVHDLHFADRPNSVSVDILWYNNSGLAFCPYGEEWRQMRKICMLEMLSPKNVKSFGYIREDEIINLVKSLRSLSSGESVDFTETISSFNCSITCRAALGKVLGDRDTLIPLIKTAVGMSGGFEVADLFPSFKLLHFLSVKKYMLPGMRRKIDAILDRMVEDHKVKKSGEYDGEDIVDVLLRMQETGELRFPVTTENIKAIILDMFAAGTETVSTSMDWIMTELMRHPRVMTKLQEEIRGALKGKTRLEESDVQGLKYMKSVIKETMRLHPPVPIIPRKCREECRIGGYSIPLNSSLIINVWSLGRDPKYWNDPETFLPERFDENSLDMLGHDFEFLPFGSGRRICPGLNFGLTNVQFTLAQLLYNFDWKLPDGMKPSDVDMSELDGLAVGRKTPLLMVPTPYNHHS

>Lamium maculatum_Cluster-9868.0

MESLSTLILLLFSSSIVLLLLIKSWRKDKNPHNFPPSPPALPLIGHLHHLSGTLVHHAMTRINKKYGPVVTLKLGEVTTVVISSRETTKEALRTNDPACADRPDSGVLEIMFYNYSDIAFGAYSEYWRQMRKICILEMLSAKNVKSFGYIRAEEMDGAVESVRSRSGEPLNFTEMIFMMTCAVTCRATFGKVLKGRETFIKLLKKMATMAGGYELTDLFPSIKILNLVSKNKAKLLKMRDEMDAILDPAVEEHRSNKSGEFEGEDIVDVLLRMQKNKELQFPITTDNIKAVILDMFTAGTETSATTTDWTMALLMKNPRVMAKLQSEIREAFKGKSSVEEKDVQKLKYLKNVVKESLRLHPPFPLLPRKCRQECKVGGYTIPNKAKVMINVGSLCRDPLYWEDPEAFQPERFDNSSIDFLGNDYEYLPFGSGKRNCPGLNFGIANVEFPLAQLLFHFDWKLPQGMAPADVDMTEQEGQPIARKNPLILLPTLYNPPSSN

>Lamium maculatum_Cluster-9204.9250

MEIITFTVLLLLPTFIFFLIKTIKKRKPEEKLPPSPPKLPLIGHLHHLAGGLPHQALSQISKKYGDIVSLKLGETNAVIVSSRELAKEAMKVHDLHFADRPNSVSVDILWYNNSGLAFSPYGEEWRQMRKICMLEMLSPKNVKSFGHIREDEIINLVKTLRSLPSGESVDFTETISIFNSSITCRAALGKVLGDRDTLIPLIKMAVGMSGGFEVADLFPSFKLLHFLSVKKYKLPGMRRKIDGILDRMVEDHKVKKSGEYDGEDIVDVLLRMQETGELRFPVTTENIKAIILDMFAAGTETVSTSMDWIMTELMRHPRVMTKLQEEIRGALKGKTRLEESDVQELKYMKLVIKETMRLHPPVPIIPRKCREECRIGGYSIPLNSSLIINVWSLGRDPKYWNDPETFLPERFEENSRDMLGHDFEFLPFGSGRRICPGLNFGLANVQFTLAQLIYNFDWKLPNGMKPSDVDMSELDGLAVGRKTPLLLVPTPYNPSK

>Lamium orvala_Cluster-6989.0

MEIISFTTLLLLSTFIFFLVKTIKKPKPSKNEEKLPPSPPKLPLIGHLHHLAGGLPHQALSKISKKYGDIVSLKLGETNAVIVSSRELAKEAMKVHDLHFADRPNSVLVDILWYNNSGLAFCPYGEEWRQMRKICMVEMLSPKNVKSFGYIREDEIINLVKTLRSLSSGESVDFTETISRFNSSITCRAALGKVLGDRDTLIPLIKTAVGMSGGFEIADLFPSLKLLHLFSVGKYKLPGMRRKIDAILDLMVEDHKLKKSGEYDGEDIVDVLLRMQETGELKFPVTTENIKAIILDMFAAGTETVSTSMDWIMTELMRHPRVMVKLQEEIRGALKGKTRLEESDVQELKYMKLVIKETMRLHPPVPIIPRKCREECRIGGYSIPLNTSLIINVWSLGRDPKYWNDPETFLPERFEENSLDLLGHDFEFLPFGSGRRICPGLNFGLTNVHFTLAQLLYNFDWKLPNGMKPSDVDMSELDGLAVGRNTPLLLVPTPYNHS

>Sesamum indicum_XM_011083632.2

MEIQLPVSFTTLLLFSSFIFLLIRAWMKPKSPRKYEKLPPSPPQLPVIGHLHHLVGGLPHHALRRVAQKYGPVLYLKLGEVSAVVISSREATKEVVKVQDPACADRPESIGSKIMWYDYTDIAFSAYNEYWRQMRKICILELLSAKNVKSFGSIRQDEVSRLMKSLQSSSGQAINLTEKVFAFTSSITCRAAFGKVMRDRDVLIAMLKKAVTMAGGFELADLFPSSKLLHVLSWNKYKLLRMRRKLDTILDAILEEHKLKQSGEFGGEDIVDVLLRMQKTGELQFPITNDNVKAVIFDMFSAGTETSSTTIDWAMAELMRNPRVMAKVQAEVREAFKGKTTVEESDVQALKYLKLVIKETFRLHPPIPLLPRACRDECKVNGYTIPLKSKVMINIWAMGRDPAYWDQPDTFQPERFDNNSIDFLGNNFEYIPFGAGRRICPGMNFGLANVELPLAQLLYHFDWKMPKGMTPDDIDMTEAEGIAVSRRNALLLVPTIYSASN

>Salvia miltiorrhiza_KP337668.1

MEMDLPFNFTTTLLLLSSFIFFLLKAWTKPKSPKAHQNLPPSPPKLPVIGHIHLLVGDLPHKALQRQAQKHGPVMHLQLGEVPAVVISSREATREVLKVQDPACADRPESIASKILWYDYTDIAFSAYNEYWRQMRKICIVELLSSKNVKSFGHIRRDESSRLMKSLECSSGNAIDLTDKIFTFTSTITCRAAFGKVMTDRDGLIALFKEAVAMAGGFELADLFPSWKLLNVLSWSKYRLWRMRGKLDAILDGIIDEHKLKQSGEFGGEDIVDVLIRMQQTGELKFPITTDNIKAVIFDMFVAGTETSSTTTVWAMSEMMRNPRVMEKAQAEVRAALKGKAAVEESDVQELKYLKLVIKETFRLHPPIPLLPRQTREECKVDGYSIPIKTKVMLNIWSMGRDPQYWEQPEKFQPERFENSPKDFIGNDFEYIPFGAGRRICPGLNFGLANIELPLAKILYHFDWKLPKGMSYERLDMSEGEGLTVSRKKPLIIIPTLCNPVN

>Lavandula angustifolia_MN822899.1

MAVELPFTFTALLLFSSFIFFLIKAWSTKPKPHQNLPPSPPKLPVIGHLHHLVGDLPHKQLKKLSTKHGPVIHLQLGEVPAVVISSREATKEVLKVHDPACADRPESIASKILWYDYTDIAFSAYNEYWRQMRKICIVELLSTKNVKSFGYIRQDEAVRLIKSLQSSSGQVVDLTDKIFTFTGTITCRAAFGRVMTDREGLIDLFKKAVAMAGGFELADLFPSWKALHLLSWNKYKLLRMRGKLDAILDRIVDEHKLKKSGEFGGEDIVDVLLRMQRTGELKFPITTDNIKAVIFDMFVAGTETSSTMTIWAMSELMKNPEAMQKAQAEVREALKGKTAVEESDVVELKYLKLVIKETFRLHPPIPLLPRQSREECKASGYSIPVKTKVMLNIWSMGRDPLYWEEPEMFRPERFENSPKDFIGNDFEYIPFGAGRRICPGLNFGLANIELPLAKLLYHFDWKLSKGTSSDLDMSEGEGLTVSRKNPLLLVPTVCYPAN

>Mentha spicata_AY622319.1

MELQISSAIIILVATFVASLLIKQWRKSESRQNLPPGPPKLPLVGHLHLLWGKLPQHAMADMAKKYGPVTHVQLGEVFSVVLSSREATKEAMKLLDPACADRFESIGTRIMWYDNDDIIFSPYSDHWRQMRKICVSELLSARNVRSFGFIRQDEMSRLLRHLQSSAGETVDMTERIATLTCSIICRAAFGAIINDHEELVELVKDSLSMASGFELADLFPSSKLLNLLCWNKSKLWRMRRRVDTILEAIVDEHKLKKSGEFGGEDIIDVLFRMQKDSQIKVPITTNAIKAFIFDTFSAGTETSSTTTLWVMAELMRNPAVMAKAQAEVRAALKGKTSVDVDDVQELKYMKSVVKETMRMHPPIPLIPRSCREECEVNGYKIPNKARIMINVWSMGRNPLYWEKPETFWPERFDQVSRDFMGSDFEFIPFGAGRRICPGLNFGLANVEVPLAQLLYHFDWKLAEGMKPSDMDMSEAEGLTGIRKNNLLLVPTLYKSP

>Olea europaea_XM_023007243.1

MEIHLLQFNFTTFLLFTSFVYLLVKGWKKSKSLKENKKYPPSPGWKLPLIGHLHLMMGALPHRSLSKLSKQYGPIIHLKLGEVSSIVISSREIAKKVLTVNDLAVADRPESIGMKTLWYDYKDLAFSPYNEHWRQMRKICIMELLSNKNVRSFGHIRLDETSNLIKSVRSHGGEPFNMTDKIFAFTSIATSRAAFGEILKEQDTLIKFMKKGVTLAGGFELADLYPSLKFLQIFSWNKHQLLKMRHKLDSILDVMLNEHKHKLARGKRDNGELGSENIVDVLLRKQQSDDLDVPITDDNIKAIIFDMFTAGTETSSTTFDWVMTELMRNPRVMAKAQAEIREAFKGKKTIEESDIQNLKYLKLIIKENFRVHPAVTLLPRACREECEVDGYTIPYKAKVAVNIWALGRDPEYWEEPEIFKPERFENSSVDFLGNNFEFIPFGSGRRFCPGMNFGIANIDVPLAQLLYHFDWKLPHGMNHSDLDMSEIPGVSVQRKNNLYLIATPYNPPIEG

>Lamium orvala _Cluster-13930.0

MVMEMEVSLLYIFVALCLIFVIVIAKRFTRKHSRLPPSPRPALPILGHLHLLKPPLHRTFHRLSQTHGPIFSLKLGVRRVVVVSSPNLVEECFTLNDIVFSNRPWVMVDKYIGYNHTTIAGAPYGQQWRNLRRLGAQEVLSASRLNSLSEIRQDEMRRTLQTLTQSEEFEKLQLRPKLFELIFNLIMRIVAGKRYSTGKGDDEQLGERIREMVSEVFEHAQSSNPEDFLPFLKWIDYRGLKKNLTNLGNKLDDFYQSLLEEHRKEKRNTIIGHLLSLQQSDPNFYTDQIIKGFITNMIIAGTDTSVVTIEWAMSLLLNHPNVLQKAKQELDSQVGHQRLVEEQDLPNLHYLQNIISETFRLFPAGPLVVPRESSADCRVGGYDIPRGTILLVNAWAIHRDPDVWDEPMSFKPERFEGREVETQTLMPFGMGRRACPGAGLGQRMVGLCLGSLIQCFEWERVCLEEIDLAEGVGLTMPKLKPLEAMCKPREIMLKALRGATNFTT

>Triticum aestivum_Bx5

MAFEGAYHFLQLAVGHATSSPAALLLVVVPLLLLLLASVRRSTMTGRKLRLPPSPPGSLPIIGHLHHIGAQTHISLQHLVDKYGHNGLLFLRAGAVPTVIVSSPSAAEAVMRTHDHILASRPWSMASHILRYNTTDVAFSPLGEYWQHTRKLVNTHLLSAKKVHSFANGRQEEVCLVVNKIREAATTAPSTAVDMSEFLAAYTNDVVSRSVLGATHRKKGRNTLFREMTETNVDLLVGFNLENFIPRWPLTEVLFRLVCWKVQRHLNKWDALLEEVIKEHINLKQDNSADFIHVFLSLQQEYGLTDDNVKSLLMNIFEAAIETSYLVLEYAMAELINNRHVMKKLQTEVRTFASSKGKKLDMITEEDLSSLPYLKATMKEALRLHPPGPLLLPHYSTADCSIDGYDIPAKTRILVNGWAIGRDPKAWERPEEFMPERFLQDGQEKSSNLGQDFKYLPFGSGRRICPGANFALATMEIMLVNLMYHFDWEVPNEKDGAGGKVSMAETFGLMLRRNEKLYLVPRIA

>Secale cereale_Bx5

MALEAAHHYLQLAVGHGTSTPAALLLVLVPLLLLLLASVRTSASTRKLRLPPSPPGSLPIIGHLHHIGAQTHISLQHLVDKYGHNGLLFLRAGAVPTVIVSSPSAAEAVMRTHDHILASRPWSMASHILRYNTTDVAFSPLGEYWQHTRKLVNTHLLNAKKVHSFRHGRQEEVCLVVNKIREAATNAPSTAVDMSEFLAAYTNDVVSRSVLGATHRKKGRNTLFREMTETNVDLLVGFNLENFIPRWPLTEVLFLRLVCWKVQRHLNKWDALLEEVIKEHMNLKQDNSADFIHVFLSLQQEYGLTDDNVKSLLMNIFEAAIETSYLVLEYAMAELINNRHVMKKLQTEVRTFASSKGKKLDMITEEDLSSLPYLKATMKEALRLHPPGPLLLPHYSTADCNIDGYDIPAKTRILVNGWAIGRDPTAWEKPEDFMPERFLQDGQEKSSNLGQDFKYLPFGSGRRICPGANFALATMEIMLVNLMYHFDWEVPNEKDGTGGKVSMAETFGLMLRRNEKLYLVPRIV

>Hordeum lecheri_Bx5

MALEAAHHYLRHAVGHGTSAPAALLLVCVPLLLLLLLFASLRTSASTRKLRLPPSPPGSLPIIGHLHHIGAQTHISLQHLVDKYGHNGLLFLRAGAVPTVIVSSPSAAEAVMRTHDHILASRPWSMASHILRYNTTDVAFSPLGEYWQHTRKLVNTHLLSAKKVHSFRHGRQEEVSLVVDKIREAATNAPSTVVDMSEFLAAYTNDVVSRSVLGATHRKKGRNTLFREMTETNVDLLVGFNLENFIPRWPLTEVLFRLVCWKVQRHLNKWDALLEEVIKEHINLKQDNSADFIHVFLSLQQEYGLTDDNVKSLLMNIFEAAIETSYLVLEYAMAELINNRHVMKKLQTEVRTFASSKGKKLDMITEEDLSSLPYLKATMKEALRLHPPGPLLLPHYSTADCNIDGYDIPAKTRILVNGWAIGRDPTAWERQEDFMPERFLQEGQEKSSNLGQDFKYLPFGSGRRICPGANFALATMEIMLVNLMYHFDWEVPNEKEGTGGKVSMAETFGLMLRRNEKLYLVPRIVE

>Zea mays_Bx5_CYP71C3v1

MALQAAYEYLQQAVGHGAWSSTQTLTLLLIAVPTVLLLLASLAKSTSSSGRGKPPLPPSPPGTLPIVGHLHHIGPQTHISLQELVAKYGHNGFLFLRAGAVPTLIVSSPSAAEAVMRTHDHICASRPWSMASHILRYNTCDVAFSPLGEYWQQTRKLMNTHLLSNKKVYSFRHGREEEVCLVVDNLREAAAKSPSTAVDMSEVLAAYTNDVVSRSVLGSTHRKKGRNTLFREMTMTNVDLLVGFNLEYYIPRWPLTDLLFRLVCWKVTRHLKRWDALLEEVIHEHVEMRKLSGDKEKESDDFIDIFLSRYEEYGFTMDNVKSLLMNVFEAAIETSYLVLESAMAELMNHRRVMKKLQAEVRAYGAEKKLDMIREDDLSSLPYLKASMKEALRLHPPGPLLLPHYSTADCQIDGYHIPANPRVLVNGWAIGRDPAVWEKPEEFMPERFMRDGWDKSNSYSGQDFRYLPFGSGRRICPGANFGLATMEIMLANLMYHFDWEVPNEKEDGCWKVSMDEKFGLMLRRNELLYLVPRASS

>Mentha spicata_CYP71D18

MELDLLSAIIILVATYIVSLLINQWRKSKSQQNLPPSPPKLPVIGHLHFLWGGLPQHVFRSIAQKYGPVAHVQLGEVYSVVLSSAEAAKQAMKVLDPNFADRFDGIGSRTMWYDKDDIIFSPYNDHWRQMRRICVTELLSPKNVRSFGYIRQEEIERLIRLLGSSGGAPVDVTEEVSKMSCVVVCRAAFGSVLKDQGSLAELVKESLALASGFELADLYPSSWLLNLLSLNKYRLQRMRRRLDHILDGFLEEHREKKSGEFGGEDIVDVLFRMQKGSDIKIPITSNCIKGFIFDTFSAGAETSSTTISWALSELMRNPAKMAKVQAEVREALKGKTVVDLSEVQELKYLRSVLKETLRLHPPFPLIPRQSREECEVNGYTIPAKTRIFINVWAIGRDPQYWEDPDTFRPERFDEVSRDFMGNDFEFIPFGAGRRICPGLHFGLANVEIPLAQLLYHFDWKLPQGMTDADLDMTETPGLSGPKKKNVCLVPTLYKSP

>Zea mays_BX3_CYP71C2

MALGAAYHHYLQLAGDHGTATHALLLGVLIFLVIRLVSARRTGTTSANKRKQQQRLPLPPWPPGKLPIIGHLHLIGAETHISIRDLDAKHGRNGLLLLRIGAVPTLFVSSPSAADAVLRTQDHIFASRPPWMAAEIIRYGPSDVAFVPYGEYGRQGRKLLTTHMLSTKKVQSFRHGRQEEVRLVMDKIRAAATAAPPAAVDLSDLLSGYTNDVVSRAVLGASHRNQGRNRLFSELTEINVSLLAGFNLEDYFPPNMAMADVLLRLVSVKARRLNQRWNDVFDELIQEHVQSRPSGESEESEADFIHVLLSIQQEYGLTTDNLKAILVDMFEAGIETSYLTLEYGMAELINNRHVMEKLQTEVRTTMGSPDGKKLDMLAEEDLGSMPYLKATIKETLRLHPPAPFLLPHYSTADSEIDGYFVPAGTRVLVHAWALGRDRTTWEKPEEFMPERFVQEPGAVDVHMKGKDLRFIPFGSGRRICPGMNFGFATMEVMLANLMYHFDWEVPGSGAGVSMEESFGLTLRRKEKLLLVPRIAS

>Zea mays_BX2_CYP71C4

MAAQLHHALYELLHEAAAAQRALLLAIPFSLLLLPLLLRYLAASASASATKNDGAAPASDPDKLLSLLPSPPMKLPIIGHLHLMGDIPYVSLAALATRYGPDLMLLRLGAVPTVVVSSPRVAEAVLRTYDHVFSSRPRSLVSDIIMYGATDSCFAPYGDHFRKARKLVTVHLLNASKVRSQRPAREEEVRGALDRVRRAAAAREPVDMSELLHSFVNNLVCRAVSGKFSMEEGRNRLFRELTDINAGLLGGFHIQDYFPRLGRIELVRKVACAKTRRVRKRWDDLLDKLIDDHAARMATHQDEDDDKDFIYVLLSLQKEYGLTRDHIKAILIDMFEAGTDTSYMTLEFAMTELIRKPHLMKKLQEEVRRNVPAGQEMVTEDNLPGMTDLKAVIKETLRLHPPVPLLLPHYSLDACEVAGYTIPANTRVVVNAWALGRHSGYWERENEFVPERFLSGDVAGGVDLKPNEFQFLAFGSGRRMCPGVHSASATIEAMLSNLMYRFDWQLPAGMKAEDVDMTEVFGITVSRKEKLLLVPQAA

>Triticum aestivum_Bx2

MAHVHVDEMLHEAAAAAPRSLLIATAVLFSLVVLPLLLRIIAKQGAASDAKLLSLLPSPPTRLPIIGHLHLMGDLPYVSLAGLAAKYGPELMLVHLGAVPTAVVSSPRTAEAVLRTHDHIFASRPRSMVFDIIMYGQTDSCFSPYGEHFRKARKLVTVHMLNARKIRSQRPAREEEVRQVIGKIAKAAAAREAVDMSELLHSYVNDLVCRAVSGKFSQEEGRNKLFRELTDINAALLGGFNILDYFPSLGRFELVCKVACAKARRVRKRWDLLLDKLIDDHAARMVSREGEAQPEQEEDKDFIDVSLSLQQEYGLTRDHIKAILIDMFEAGTDTSYMTLEFAMAELIRKPHLMKKLQEEVRRNVPNGQEMAAEDDLPNMTYLKAVIKETLRLHPPVPLMIPHFSLDACTVDGYTIPANTRVVINAWALGRHSGYWENENEFQPERFMNGAGVDLKPNEFHYLPFGFGRRMCPGVHSASATVETMLANLMYRFDWKLPPGLKEENIDMTEVFGITVSRKEKLILVPVTA

>Triticum aestivum_Bx3

MALEAAFHYLQLAGVHGTSTPALLLTVLLLLIIRLAWVRTTTVSTRFGKQQQLPPSPPGKLPIIGHLHLLGSQTHISIRDLDAKHGRNGLLLLRIGAVPTLFVSSPSAAEAVLRTHDQIFASRPPSMAADIIRYGPTDIAFAPYGEYWRQARKLLTTHMLSAKVVHSFRHGRQEEVRLVINKIREAATRGTVVDMSDLLSGYTNDVVCRAVLGESHRKAGRNRLFSELTEINVSLLGGFSLENYIPPNMVMADVLLRLVSVKAQRLNKRWDDLFNEIIEEHLHPSKPSSGEQQAADFIDLLLSLKEEYGLTTDNIKAILVDMFEAGIETSYLTLEYGMAELMNNRHILKKLQEEVRSQGKKLDMITEEDLSSMAYLRATIKETLRMHPPAPFLLPHFSTADCKVDGYLIPANTRVLVNAWALGRDPSSWERPDDFWPERFLQDQAGDVDTQMRGKDLRFLPFGFGRRICPGMNFGFATMEVMLANLMYHFDWDVPNMMGTGAGVDMAESFGLTLRRKEKLQLVAQIP

>Triticum aestivum_Bx4

MALEAAYHYLQRAVGHGTSTEALLLTVLLLLIIRLAWVRAFATTTASTKCKQQLPPTPPGKLPIIGHLHLIGSHPHVSFRDLAAKHGRDGLMLVHVGAVPTVVVSTPQAAEAVLRTHDHVFASRPRNPVADIIRYNSTDIAFAPYGDYWRRARKVVNTHLLSVKMVYSKRHDREEEVRLVVAKIRELAMAAPGKALDMTELLGGYASDFVCRAVLGESHRKHGRNELFRELTEISASLLGGFNLEDYFPRLANLDVFLRVVCSKAMGVSKRWDNLFNELIAEYEHGKEDNAEDFVHLLLSLKKEYGLSTDNVKAILVNMFEAAIETSFLVLEYSMAELINNRHVMAKVQKEVRESTPHGEKLDLIMEEDLSRMPYLKATIKEAMRIHPPAPFLLPHFSTNDCEINGYTIPAGTRVIVNAWALARDPSHWERAEEFYPERFLQEGRDAEVDMYGKDIRFVPFGAGRRICAGATFAIATVEVMLANLIYHFDWELPSEMEAIGAKVDMSDQFGMTLRRTERLHLVPKIYK

>Secale cereale_Bx2

MLHEAAAAAPRSLLVATAVLFSLVVLPLLLRIITKQGAASDAKLLSLLPSPPTKLPIIGHLHLMGDLPYVSLAGLAAKYGPELMLVHLGAVPTAVVSSPRTAEAILRTHDHIFASRPRSMVFDIIMYGQTDSCFAPYGEHFRKARKLVTVHMLNARKIRSQRPAREEEVRLVIGKVAKAAAARESVDMSELLHSYVNDLVCRAVSGKFSQEEGRNKLFRELTDINAALLGGFNILDYFPSLGRFELVCKVACAKARRVRKRWDLLLDKLIDDHAARMVSREDEAQAEQEEDKDFIDVSLSLQQEYGLTRDHIKAILIDMFEAGTDTSYMTLEFAMAELIRKPHLMKKLQEEVRRNVPNGQEMVAEDDLPNMTYLKAVIKETLRLHPPVPLMIPHFSLDACTVDGYTIPANTRVVVNAWALGRHSGYWENENEFQPERFMNGAGVDLKPNEFHYLPFGFGRRMCPGVHSASATVETMLANLMYRFDWKLPPGLKEEDIDMTEVFGITVSRKEKLILVPVAM

>Secale cereale_Bx3

MALEAAYHYLQLAGIHGTSTPAVLLTVLLLLIVRLAWVRTTTASTRFGKQQQLPPSPPGKLPIIGHLHLLGSQTHISIRDLDAKHGRNGLLLLRIGAVPTLFVSSPSAAEAVLRTHDQIFASRPPSMAADIIRYGPTDIAFAPYGEYWRQARKLLTTHMLSAKVVHSFRHGRQEEVRLVINKIRAAATRGTAVDMSELLSGYTNDVVCRAVLGESHRKAGRNRLFSELTEINVSLLGGFSLENYIPPNMVMADVLLRLVSVKAQRLNKRWDELFNEIIEEHLHPSKPSSGEQQAADFIDLLLSLKEEYGLTTDNIKAILVDMFEAGIETSYLTLEYGMAELMNNRHILKKLQEEVRSQGKKLDMITEEDLSSMAYLRATIKETLRLHPPAPFLLPHFSTADCKIDGYLIPSNTRVLVNAWALGRDPSSWERPEDFWPERFLQDQAGDVDTQMRGKDLRFLPFGFGRRICPGMNFGFATMEVMLANLMYHFDWDVPNIMGTGAGVDMAESFGLTLRRKEKLQLVPQIP

>Secale cereale_Bx4

MALEAAYHYLLLIIRLAWVRAFATTAASTKGKQQLPPTPPGKLPIIGHLHLIGSHPHVSFRDLAAKHGRDGLMLVHVGAVPTVVVSTPQAAEAVLRTHDHVFASRPRNPVADIIRYNSTDIAFAPYGDYWRRARKVVNTHLLSVKMVYSKRHDREEEVRLVVGKIRELAAAAPGKALDMTELLGGYASDFVRRAVLGESHRKHGRNELFRELTEISASLLGGFNLEDYFPRLANLDVFLRVVCSKAMGVSKRWDNLFNELIAEYEGGKEDNAEDFVHLLLSLKKEYGLSTDNVKAILVNMFEAAIETSFLVLEYSMAELINNRHVMAKVQKEVRESTPNGEKLDLIMEEDLSRMPYLKATIKEAMRIHPPAPFLLPHFSTNDCEINGYTIPAGTRVIVNAWALARDPLHWERAEEFYPERFLQEGRDAEVDMYGKDIRFVPFGAGRRICAGATFAIATVEVMLANLIYHFDWALPSEMEAIGAKVDMSDQFGMTLRRTERLHLVPKIYK

>Hordeum lecheri_Bx2

MAHVHVDEMLHGAAAAPRSLLIATAVLFSLVVLPLLLRIITKQGAASDAKLLSLLPSPPSKLPIIGHLHLMGDLPYVSLAGLAAKYGPELMLVRLGAVPTAVVSSPRTAEAVLRTHDHVFASRPRSMVFDIIMYGQTDSCFAPYGDHFRKARKLVTVHMLNARKIRSQRPAREEEVRLVIGKIAKAAAAREAVDMSELLHSYVNDLVCRAVSGKFSQEEGRNKLFRELTDINAALLGGFNILDYFPSLGRFELVCKVACAKARRVRKRWDLLLDKLIDDHAARMVSREDEAQGEQEEDKDFIDVSLSLQQEYGLTRDHIKAILIDMFEAGTDTSYMTLEFAMAELIRKPHLLNKLQEEVRRNVPNGQEMVAEDDLPNMTYLKAVIKETLRLHPPVPLMIPHFSLDACTVDGYTIPANTRVVINAWALGRHSSYWENENEFQPERFMNGAGVDLKPNEFHYLPFGFGRRMCPGVHSASATVETMLANLMYRFDWKLPPGLKEEDIDMTEVFGITVSRKEKLILVPVTV

>Hordeum lecheri_Bx3

MALEAAYHYLQIAVGHGTSTPAALLTVLLLLIIRLAWVRTTTASTRLSKQQQLPPSPPGKLPIIGHLHLLGSQTHISIRDLDAKHGRNGLLLLRIGAVPTLFVSSPSAAEAVLRTHDQIFASRPPSMAAAIIRYGLTDIAFAPYGEYWRQARKLLTTHMLSAKVVHSFRHGRQEEVRLVINKTREAATRGTAVDMSELLSGYTNDVVCRAVLGESHRKEGRNRLFSELTEINVSLLGGFSLENYIPPNMIMADVLLRLVSVKAQRLNKRWDDLFNEIIEEHLHPSKPSSGEQQAADFIDLLLSLKEEYGLTTDNIKAILVDMFEAGIETSYLTLEYGMAELMNNRHILTKLQEEVRSQGKKLDMITEEDVSSMAYLRATIKETSRLHPPAPFLLPHFSTADCNIDGYVVPSNTRVLVNAWALGRDLSSWERPDDFLPERFLQDQAGDVDTQMRGKDLRFLPFGFGRRICPGMNFGFATMEIMLANLMYHFDWDVPNMMGTGAGVDMAESFGLTLRRKEKLQLVPQIP

>Hordeum lecheri_Bx4

MALEAAYHYLQRAVGHGTTTEALLLTVLLLLIIRLAWVRAFTSTTTSTKFKQQLPPTPPGKLPIIGHLHLIGSHPHVTFRDLAAKHGRDGLMLVHVGAVPTVVVSTPQAAEAVLRTHDHVFASRPRNPVADIIRYGSTDIAFAPYGDYWRRARKVVNTHLLSVKMVYSKRHDREEEVRLVVAKIHELAMAAPGKALDMTELLGGYASDFVCRAVLGESHRKHGRNELFRELTEISASLLGGFNLEDYFPRLANLDVFLRVVCSKAMGVSKRWDNLFNELIAEYEGGKEDNAEDFVHLLLSLKKEYNLSTDNVKAILVNMFEAAIETSFLVLEYSMAELINNRHVMTKVQKEVRESTPEGGKLDLIMEEDLSRMPYLKATIKEAMRVHPPAPFLLPHFSTNDCEVNGYTIPAGTRVIVNAWALARDPSHWERAEEFYPERFLQEGRDAEVDMYGKDIRFVPFGAGRRICAGATFAIATVEVMLANLIYHFDWELPSEMEAVGAKVDMSDQFGMTLRRTQRLHLVPKIYK

>Zea mays_Bx4_CYP71C1

MALEAGYDYLHVAVVQCTPTQAAAVLGVLLLLAIRLAAAARSSSATSPKWKQHRLPPTPPGKLPIIGHLHLIGSHPHVSFRDLHAKYGHNGLMLVQVGAVPTIVVSTPQAAEAVLRTHDHVLASRPRNPVADIIRYNSTDVAFAPYGVYWRTARKVVNTHLLSAKMVFSKRREREEEVRLVVARIRDAAEASPGTALDMTELLGGYASDFVCRAVLGESHRKQGRNKLFRELTETSAALLGGFNVEDYFPKLADVDLFLRIICAKAKSVSKRWDSLFNELLSEYALSGGKQGDHNSEDFVHLLLSLQKDYGLTTDNIKGILVNMFEAAIETSFLVLEYSMSELMNNRHVLAKLQKEVRTATPDGRMVMEEDLSRMPYLKATIKESMRIHPPAPFLLPHFSTHDCEINGYTIPAGTRVIVNAWALARDPTCWDKAEEFFPERFLEQGRDAEVDMYGKDIRFVPFGAGRRICAGATFAIATVEIMLANLIYHFDWEMPAEMERTGAKVDMSDQFGMTLRRTQKLYLVPRIPKCVSSS

>Leonotis leonurus_LELE_c28478_g1_i1_len_1765

MEIFQLIPLNFTTVVLISSFVFFLIKLIIKPKSPQNLPPAPPGLPVIGHLHHLAGGLVHEGLAKLSKKYGPIVSLNLGQVPAVAISSRELAKEVLKVLDPACADRPESIALRTMFYGNTDIAFSPYNEYWRQMRKICILEMLSAKNVKSFGYIRQDETNNLVNSLKSVSGKAINLTERMFVFSCTVTCRATFGNVMRDRDLFIVLVKKLAHMAGGMELADLFPSIKSLTVFTWNRFQLWKMKRGMDAILDPIVAEHRLKRSGEGDSEDIVDVLLRVQESGQLQFPITNDNVKAVILDMFAAGTESSATTTDWAMAELMRNPRVMARVQSEIRETLKGKTTVEESDVPKLTYLKLVIKETLRLHPAFPLLPRQCKDEFEVGGYTIPVKTKVMINVWALARDPKYWEDAESFKPERFENNPIDFLGSDYEFLPFGAGRRNCPGLSFGIANLELPLAQLLFHFDWKLPQGMRPEDVDMAAVEGLAVGRKTPLVLIPTVHNPSN

>Lamium album_LAAL_c35486_g2_i3_len_1678

MEIQLPFTFTTLLLFSSLSFLLIKAWIKPKSQTKHKNLPPSPPKLPVIGHLHHLVGELPHRALTRVAKQYGPVLHLQLGEVSAVVISSPEAAKEAVKTRDPACADRPDSIGTKIMWYDHADIAFSPYNEYWKQMRKICILELLSAKNVKSFGDIRRDEVSRLVESLKLSSGEAVDLRKKVFGVTSSITCRSAFGKVVKQSDALIEMVSTAATMAGGFELADLFPSSKLLNLLTWNKYKLLRMRRKLDVIIDGIVEEHRLKKSGEFDGEDIVDVLLRMQRNRELKFPITNHNVKAVVFDMFSAGTETTSSTIDWAMAELMRNPDVLAKAQQEIRDGLKGKLRVEDSDIQGLKYLKLVIKETLRLHPPIPLLPRACRDECEVDGYTIPVKSKVMINIWSMGRDPEYWDEPEAFRPERFEESSIDFMGNNFEYIPFGAGRRVCPGLNFGLANVGYQLGQLLYNFDWEIPRGMSRDDIDMTEAEGIAVSRKSGLFLVPILHDFSTEN

>Lamium album_LAAL_c18029_g1_i2_len_1651

MEIISLTTLLLLSTFIFFLIKTIKKPKPSENEGKLPPSPPKLPLIGHLHHLAGDLPHHALSKISKKYGDIVSLKLGETNAVIVSSRELAKEAMKVHDLHFADRPNSVSVDILWYNNSGLAFCPYGEEWRQMRKICMLEMLSPKNVKSFGYIREDEIINLVKSLRSLSSGEAVDFTETISSFNCSITCRAALGKVLGDRDTLIPLIKTAVGMSGGFEVADLFPSFKLLHFLSVKKYMLPGMRRKIDAILDRMVEDHKVKKSGEYDGEDIVDVLLRMQETGELRFPVTTENIKAIILDMFAAGTETVSTSMDWIMTELMRHPRVMTKLQEEIRGALKGKTRLEESDVQGLKYMKSVIKETMRLHPPVPIIPRKCREECRIGGYSIPLNSSLIINVWSLGRDPKYWNDPETFLPERFDENSLDMLGHDFEFLPFGSGRRICPGLNFGLTNVQFTLAQLLYNFDWKLPDGMKPSDVDMSELDGLAVGRKTPLLMVPTPYNHHS

>Ballota pseudodictamnus_BAPS_c49177_g1_i1_len_1814

MEIQLPFTLTLTTLLLFSSFIFLLVKTCEKSKSPPKHKNLPPSPPKLPVIGHLHHLVGELPHHALTRVAKKYGPVLHLQLGEVSAVVISSREAAKEAVKVQDPACADRPDSIGTKIMWYDHADIAFSPYNEYWKQMRKICILELLSAKNVKSFGDIRQDEVSRLVESLRLCSGEAVDLRKKVFGVTSSITCRSAFGKVVKQSDALVEMLSTAVTMAGGFELADLFPSSKLLNLLTWNKYKLLRMRRKLDVIIDEIVEEHRLKQSGEFDGEDIVDVLLRMQRNRELEFPITNHNVKAVVFDMFSAGTETTSSTIDWAMAELMRNPEVLAKAQREIRDGLKGKSTVEESDMQGLKYLKSVIKETLRLHPPIPLLPRACRDECIVDGYTIPVKSKVMINIWSMGRDPEYWNEPETFRPERFEESSVDFTGNNFEYIPFGAGRRICPGINFGLANVGYQLGQLLYHFDWEIPRGMSRDDIDMTEAEGIAVSRKSGLFLVPIFRSFSTGTSK

>Leonurus cardiaca_LECA_c32589_g1_i2_len_1625

MEMIQLSPFNVTSLLLLASFIFLLIKATKKPKPPINLPPSPPKLPVIGHLHHLAGELGHRALRKISQNYGPIVRLKLGEIESVVISSKEASKEVLKTHDPAFADRPGSIAIKILWYNQTDLAFCAYDDYWRQMRKICILEMLSAKNVKSFGYIREDEISRLVESLQSRAGQTVNFSEKISAFMSSITCRAAFGKVLGDRDTLIALVNTASSMAGGFELADFFPSFTLLHLFSWRKFTLLRMRRKIDAILDRIVEDHRLKRSGEYDGEDIVDVFLRIQNTGELKFPITTDNIKAIILDVFAAGTETSSITVGWIMTELMRNPRVMAKLQQEIRGVLKGKMRLEESDVQGLKYMKLVIKESMRIHPPVPLIPRQCKDECEVGGYTIPVKTRVMVNVWAMGRNSEYWDDPESFYPERFENVSTELLGNDFELTPFGAGRRICPGLNFGLANVEFPLAQLLYHFDWKLPEGMSPADVDTSEVEALTVGRKNPLMLIPTPYNPTDNS

>Leonotis leonurus_LELE_c33776_g2_i2_len_1724

MEIQLPFTTLLLFSPFIFLLIKAAWKKSPSSHKHKNLPPSPPKLPVIGHLHHLVGELPHRALTRLSKKYGPVLHLQLGEVPAVVISSPEAAKEAVKVHDPACADRPDSIGTEIMWYGGAAIAFSPYNGYWKQMRKICVLELLSARNVKSFGDIRRDEVRRLVESLRLCSGKAVDLKRKVSGVMSSIICRSAFGKVVKQSDTLIEMLRTAVTMTGGFELADLFPSSKLLNLLTWNKYRLLHMRRKLDAIIDGIVEEHRSKQSGEFDGEDIVDVLLRMQRNQELHFPITNHNVRAIVFDLFSAGTETTSATMDWAMAELVRNPSVLAKAQGEIRDALLGKSRVEDSDMQRLKYLKLVIKETLRLHPPIPLLPRACREECQVAGYTIPIKSKVMINIWSMGRDPDYWEEPEAFRPERFEESSIDFTGNNLEYIPFGAGRRICPGIHFGLANVDYQLAQLLYHFDWEFPPGMSRDDVDMTEAEGLAVSRKTGLFLVPILHHFSTAN

>Leonurus cardiaca_LECA_c29582_g1_i2_len_3030

MPLSLSVFLPTMAAQAALFNFVIFLLSLMIKKGEKFKSPNKHQRLPPGPPKLPLIGHLHHIVGSLPHHYPLRDLANTYGPLMHLKFGEVSTIIVSSPDIARKLLKTHDLACADRPKTVATEIFWYNYTDIVFSPYGDYWKQMRKICILELLSAKNVRSFEYIRKDEASVLIKYIQTTCGVPFDLTDKIFLFMNSMTCRAAFGNVSMDRGGAIKLIKEAVGMAGHFDLSDLFPSIKVFNFMSWNKVKMLKVRKKLDMILDNIIDEHKKNLALRRKGFGELGTEDLVDVLLRFKESGEVELPITDDSIKSVIFDMFAAGTESSSTTVDWAMAELMKNPRVMAKTQDEIRQAFNGKETIKESDIRALKYLKLVINETLRLHPPVSIIPRACREEFEIDGYHIPLNANVIVNIWSIGRDPNYWHNPESFEPERFEKNPIDYLGNHFEYIPFGSGKRICPGMTFGVANVEFLLVQLLYYFDWKLPEGISIEDVDMTEVDGLAVSRKNSLYLIATPYASAFEEKEDITSASCSR

>Jatropha curcas_CYP71D495

MLFFITVLFIFIALRIWKKSKANSTPNLPPGPNKLPLIGNVHNLVGDLPYHRLRDLSKKYGPIMHLQLGENTTVVISSPELAQEVMKTHDVNFAQRPFVLAGDIVSYKCKDIAFAPYGEYWRQLRKMCSLELLTAKRVQSFKSIREEEVSKLVESISSSSGSPINFSKMASSLTYAIISRAVCGKVSRGEEVFVPAVEKLVEAGRSISLADLYPSVKLFNALSVVRRRVEKIHGEVDKIIENIVIEHRERKRMAHAGINSKEEEDLVDVLLKFQENGDLDSYLSNDGIKAVILDMFIAGSDTSSTTIEWAISEMVKNPSIMEKAQAEVREVFGSKGKVDEADLHELNYLKLVIKETLRLHPAVPLLLPRQSREDCVIEGYNIATKSTVIVNAWAIARDPKYWDEAERFYPERFINSSIDFKGTNFEFIPFGAGRRMCPGMLFGLASVELPLAQLLYHFDWKLPGGQKPEDLDMSDDLDGTATRRHALYLTATPYLPSAVGKISR

>Consolida orientalis_TRINITY_DN2933_c0_g2_i3

MLSSSFSFSWTWIFSVFILVSYLVLSLQKKKRVSNLPPGPKGLPILGNFLMLRGKFPHRVLQRLSKQHGPIMYLRMGSVPTIIVSSPQAAELFLKTYDLTFASRPFSEAAKYFTYGRKNLAWAEYGSYWRGIRKMCHRELLSSPKVESFKSMRREEANLFVESLKEDSRKSSFAVDISSKVLAITADMSFRMILGSKYTQSGSGGQHQRRIQAVFQQMQSLDSVFNIADHYPCLSGLDLQGLAKRMKAVSKDFDDMFEKILDEHLNVGFDRGRSPDFVDLLLFSMDSKENEVQIDRSNIKAILLDMLSGSIGTTATTINWAFSEILKNPSIMKKAREEIQSFVGMDRMVEESDLVNLEYLDMVIKESLRLHPPGPLLLPHESTKDCTVNGYHIPEKSRIMINTWTIGRDPDAWDDAEEFRPERFAGKNIDYIGLNFEFLPFGSGRRRCPGIQLGLTMARLVLAQLVHCFDWELSNGMLCTTSAWTKSFRMQM

>Pyrus communis_CYP736A1

MSPPEIAILILVFLTFLWSLLRLINVSSRQSRTLPPGPAALPIIGNLHMLGDLPHRSLQNLAKKYGPIMSMRLGSVPTIVVSSPKTAKLFLKTHDTIFASRPKLQASEYMAYGTKAMAFTEYGPYWRHIRKLCTLQLLCPSKIESFAPLRREEVGLLVQSLKVAAEAGEVVDFSEKVGELVEGITYRMVLGRKNDDMFDLKGIIEEALFLTGAFNISDYVPFLSPLDLQGLTKRMKRVSKTVDQLFEKIIQDHEQVSRSEQGNHHKDFVDVLLSSIHQTLKPNDEEVYMLERTNAKATLLDMIAGAFDTSATAIIWTLAELLRHPKVMKRLQEELQSVIGMDRMVEESDLPKLDYLSMVVKESFRLHPVAPLLVPHQSMEDITVDGYHTPKKSRIFINIWTIGRDPKSWDNAEEFYPERFMNRNVDLRGHDFQLIPFGSGRRGCPAMQLGLTTVRLALGNLLHCSNWELPSGMLPKDLDMTEKFGLSLSKAKHLLATPTCRLYNES

>Panax ginseng _CYP736A12

MFPLAYPLLFVLLGALSWWILPIISPLKRHHKLPPGPRGLPIIGSLHTLGALPHRTLQTLAKKYGPIMSMRLGSVPTIVVSSPQAAELFLKTHDNIFASRPKLQAAEYMSYGTKGMSFTAYGPHWRNIRKFVVLELLTPAKINSFVGMRREELGMVVKSIKEASAANEVVDLSAKVANIIENMTYRLLLGRTKDDRYDLKGIMNEALTLAGRFNIADFVPFLGPLDIQGLTRQFKDTGKRLDKILEFIIDEHEQNSSNGNASGDFIDDMLSLKNKPSNTHDELSKVIDRSVIKAIMIDIISAAIDTSDTSIEWILTELIKHPRAMKKCQEEIDAVVGVDRMVEETDLPNLEYVYMVVKEGLRLHPVAPLLGPHESMEDITINGYFIPKQSRVIVNSWALGRDPNVWSEDADEFLPERFEGSNIDVRGRDFQLLPFGSGRRGCPGMQLGLITVQLVVARLVHCFDWNLPNGITPDNLDMTEKFGLTTPRVKHLLAVPKYRL

>Medicago truncatula_CYP736A104

MILICIAIFLVSLAFFSQWRNMNKVKRLPPGPVGLPILGSLLKLGANPHLDLHKLSQKYGPIMHLRLGLVPTIVVSSPQAAELFLKTHDIVFASRPPIEAAQLMFYNQKDVSFSVYGSYWRNMRKMCTLELLSHTKINSFRSMREQELDLLIKFIREAANDGTTVDISAKVAALTADMTCIIVFGKKYSDKDLNEKGFKASMQELMSLAATPNIADFIPYIGALDLNGLTRRMKAIGKIFDEFLEKIIDEHIQSENKDDNKTKDFVDVMLGFVGTEESDYRIERSNIKGIMMDMLIGSTDTSATSIEWTISELLKNPRVMKKVQKELETVVGMKRKVEESDLDKLEYLNMVIKESLRIHPVVPLLVPHQSMEDCTVEDFFIPKNSRIIVNGWAIMRDPNSWTDPEKFWPERFEGNNIDVGGHDFQLIPFGSGRRGCPGLHLGLTMVRLVVAQIVHCFDLKLPNDMLPSDLDMTEAFGITMPRANHLIALPVYRL

>Picea sitchensis_CYP736B1

MANELGSHGSAVLECAEFYSKLATVLLALVAAWVFLFRERKPRLPPGPFPLPIIGNFHLLGQLPHQTLAALSLKYGPLMSLRLGSALTLVVSSPDVAKEFLNNHDRVFAHRPASAAGKYLMYNFSDIVFSPDGAYWRQLRKLCALQLLNARSIESLSCTREEEVSAMICSIINSDRPVSVTKTVSAVTNAMICRMAFGRKYSDQDLIDSRGVISTIKETTLLLGSPNIGDYIPYLACLDLQGINRRLKKLNKTLDGLLEKIIDEHVSQNNPDAAPDLVDVLLAASADEAREFQISRDNIKAVINDILVAGTYMSAIVIEWAMSEVLRNPTVLKKLQDELERVIGMERMVRESDLPSLVYLQAVAKETLRLHPPAPLGLPHLSMEDCTVLGYEIPRGTRLLINLWAIGRNPNSWEDAESFKPERFMEDGSVGSKVENFESIPFGAGRRGCPGRELATRVLEFVVAQLLQCFNWKLPDEISCDQELDMTEGINGPTIPRKHELFAVPTPRLPIPLLC

>Consolida regalis_TRINITY_DN18762_c0_g2_i1

MLTGTLNMLSSSLCEYWIWITTVFILATYLVRSLQEKKRALNLPPGPKGLPILGNFHMLRGKLPHRQLHHLSKKHGPIMYLRLCSVPTIVVSSPRAAELFLKTHDLIFANRPFSEAAKYMTYDRKGLAFAEYGHYWRSLRKLCTLELLSNPKIESFKSMRMEEMKLFIASLKEASRKGLEVDLTNKVKALTTDLSFRIVLGNKYINSEDQRGFHAIAEEGDRLAGLFNIADYFPCLSGLDLQGLAKRMKAVSRVYDHIFDKIIDQHLNLEADEGRTPDFVDVMLSYMDSHENEIQIDRTNVKAVLLDMLAGSIDTSSTAIDWAFSELLKNPSVMRKVRKEIESFVGLDRVVAESDLANFEYLDMVIKESFRLHPVVPLLMPHESTEDCAVDGYHIPQKSRIIINTWSIGRDPQAWDDPEEFRPERFLGKDIDYIGRNFQFLPFGSGRRSCPGIQLGLTVIRLVVAQLVHCFDWELPNGMLCSGIDMDEEFGTTMLRASNLSVIPNYRLGN

>Consolida regalis_TRINITY_DN7035_c0_g1_i3

MESWLPAILFTAISCVFTISYLLFWSRNGSPGLPPSPPGLPLIGHLHLLGELPHHTLRKLAKQYGPIMSIRLGLVPAIIVSSPEYAELFLKTHDLVFASRPKLQAADYMSYGQKNLVFAKYGPYWRNIRRLCTIELLSTTKIEMFRGMRQQEVTAFVRSMKHTTMAGRVVVDVSAKIESLVEDMTYLLVLGRKEDMLNFKEGLQEGVRLAGLFNIADYVPSLGAFDLQGLKPRMEAVTTVLDRHLEKIIDDHVREAKELNGRHRDFVDVMLSLMECNNERQLQIERDHIKAILLDMLAAGMDTSSTTIEWVMAELLKHPRVMKLVQQELETVVGLDRMVEEADLIKLDYLNLVIKESMRIHPVAPLLIPHESMEDITINSYFIPKKSRVLINTWAIGRDMNVWSDNAEEFYPERFVGTDIDMLGHDFRLLPFGSGRRQCPGMQLGMTMVKIIVAQLVHCFNLELPDGMSPEELDMTEKFGLTLPRANHLKAIPIYRLPADL

>Coptis_deltoidea_CNA0013856_10537

MYSSFSWTWTSLVLVLLSYVFYALRKKRNNKYSESLPPGPKGIPILGNLLMLGELPHRDLHHLSKQYGSIMYLRLGFIPTIVVSSPQAAELFLKTYDLNFASRPFNEAAKYISYERKGLSFAEYGSYWRNVRKLCTLELLSNLKIESFKTMRREELGLFIESLKEASRDHVEVDLSAKVLTLSTDMSCLMVFGKKRIHNEHEQKGFHAVVQEGMQLAALFNIADYIPYVGALDLQGLAKRMKVVSKVFDDMFEQIIDEHVDAGDKGQHRDFVDVMLSFMESKENEFQIERSNIKAIILDMLVGSMDTSSTAIEWVFSELLKHPNVMKKVQQEIERVVGQDRLVEESDLTNLEYLDMVVKEALRLHPVAPLLIPHESIEDCTVNGYHIPQKSRIIVNTWAIGRDPSVWDDAENFIPERFIGSDIEYKGRDFQLLPFGQGRRGCPGMQLGLTVVRLVAAQLVHCFDWELPNGMQCTELNMDEEFGLTMPRAKHLSVIPTFRLRI

>Podophyllum_peltatum_WFBF_scaffold_2001851

MSLTWTWFLLVSFIFFLYILKKRSNPTNLPPGPKGYPILGNLPMLGKSPHRDLHRFSEEYGPIMYLSLGYVPVIVVSSPQAAELFLKAHDLIFASRPFTEAAKLAAYDRKGLSFTEYGPYWRSIRKLCTLQLLNSQKIDSFRSVRKEEVGLVGESLKDAAKTHVHVDLTAKISTVSMDISCLMIFGKKYMKTDDSQSNFLATVHEGMQLAATPNMADYIPYIGALDLQGLTKRLKVVGKVFDELFEKVIQEHILEADQGHHRGDFVDVMLSLMNSKDNEFQIERPNMKAIMLDMIAGSMDTSSTAIEWIIAELLKNPRVMKKVQEELESVVGLDRMVEESDLMNLEYLDMVVKESLRLHPVAPLLIPHESMEDCTISGYHIPKKSRIIVNSWAIGRDPSVWTDAEKFYPERFVGSNIDIRGRDFELIPFGSGRRGCPGMQLGLTVVRLVVAQLVHCFDWELANGMKPSELDMDEEFGLTMPRANHLSVIPTYRLGV

>Epimedium_brevicornu_CNA0013804_23055

MNCNLSQFTLKSFSLSPNMPWTWTWFFLVSFLSSLYILKKKLSNKSTSLPPGPIGFPILGHLPMLGKSPYLDLHRLAKKYGPIMYLRLGFVPTIVVSSPQAAELFLKVHDLVFASRPLTEAAKHVVYENKGLSFTAYGPYWRNIRKLCTLQLLSSHKIDSFKPMRKEEVGLFVESLKEDARTSTQVDLTAKICNLSMDMSCLMVVGKKYMKSGGHDQNNFISVIQDGMQLAAAPNMADYIPYIGALDLQGLTKRLKAMSKVFDNLFEKIIEDHILEAEKVHNREDFVDVMLSLMNSKDNEFQIDRSNIKAIMLDMIAGSMDTSSTAIEWAFAELLKNPPTMRKVQEELERVVGLDRTVEETDLTNLEYLDMVLKESLRLHPVAPLLIPHESIEDCTVAGYHIPRKSRIIVNSWAIGRDPSVWPDPEKFYPERFLGSNVDLRGRDFELLPFGSGRRGCPGMQLGLTVVRLVVAQLAHCFDWELPNGMLPSELDMDDEFGLTMPRANHLSVIPTYRLCV

>Epimedium_brevicornu_CNA0013804_23053

MNCNLSQFTLKSFSLSPNMPWTWTWFFLVSFLSSLYILKKKLSNKSTSLPPGPIGFPILGHLPMLGKSPYLDLHRLAKKYGPIMYLRLGFVPTIVVSSPQAAELFLKVHDLVFASRPLTEAAKHVVYENKGLSFTAYGPYWRNIRKLCTLQLLSSHKIDSFKPMRKEEVGLFVESLKEDARTSTQVDLTAKICNLSMDMSCLMVVGKKYMKSGGHDQNNFISVIQDGMQLAAAPNMADYIPYIGALDLQGLTKRLKAMSKVFDNLFEKIIEDHILEAEKVHNREDFVDVMLSLMNSKDNEFQIDRSNIKAIMLDMIAGSMDTSSTAIEWAFAELLKNPPTMRKVQEELERVVGLDRTVEETDLTNLEYLDMVLKESLRLHPVAPLLIPHESIEDCTVAGYHIPRKSRIIVNSWAIGRDPSVWPDPEKFYPERFLGSNVDVRGHDFELLPFGSGRRGCPGMQLGITVVRLVVAQLAHCFDWELPNGMLPSELDMDDEFGLTMRRANHLSVIPRYRLRV

>Podophyllum_peltatum_WFBF_scaffold_2001850

MLGEFPHRDLHRFAKEHGPIMYLRLGFVPAIVVSSPRAAELFLKAHDLIFASRPFTEAGKHVSYDRKGMSFTEYGPYWRNIRKLCTLQLLSSQKIDSFKSMRKEELGLVVESLKEEAATHVHVDLTAKISTVSMDISCLMIFGKKYMKTDDSQSNFLATVHEGMQLAATPNMADYIPYIGALDLQGLTKRLKVVGKVFDELFEKVIQEHILEADQGHHRGDFVDVMLSLMNSKDNEFQIERPNMKAIMLDMIAGSMDTSSTAIEWIIAELLKNPRVMKKVQEELESVVGLDRMVEESDLMNLEYLDMVVKESLRLHPVAPLLIPHESMEDCTISGYHIPKKSRIIVNSWAIGRDPSVWADAEKFYPERFVGSNIDVRGRDFELLPFGSGRRGCPGMQLGLTVIRLVVAQLVHCFDWELANGMKPSELDMDEEFGLTMPRANHLSVIPTYRLGV

>Epimedium_brevicornu_CNA0013804_23050

MNCNLSQFTLKSFSLSPNMPWTWTWFFLVSFLSSLYILKKKLSNKSTSLPPGPIGFPILGHLPMLGKSPYLDLHRLAKKYGPIMYLRLGFVPTIVVSSPQTAELILKVHDHVFASRPLTEAAKQVAYETKGLVFSTYGPYWRNIRKLCTLQLLSSYKINSFKSMRREEVGMLVESLKEDARTSTQVNLTAKISSLSIDMSCLMVVGKKYLKSGGHDQNNLVSVIQEGMQLAAAPNMADYIPYIGALDLQGLTKRLKAMSKLFDDLFERIIEDHIIEAEKAHDRGDFVDVMLSLMNSKDNEFHIDRSNIKAIMLDMIGASVDTSSTAIEWAFAELLKNPPIMRKVQEELERVVGLDRMVEEADLTNLEYLDMVVKESLRLHPVAPLLIPHESMEDCTVAGYHIPRKSRIIVNSWAIGRDPSVWLDAEKFYPERFLGSNVDVRGHDFELLPFGSGRRGCPGMQLGITVVRLVVAQLAHCFDWELPNGMLPSELDMDDEFGLTMRRANHLSVIPRYRLRV

>Papaver_rhoeas_QZBA_scaffold_2025919

MIGNFPHRDLHRLSTQFGPIFYMRLGFVPTIVVSTPQAAELFLKTHDLTFAGRPFSSAAKYISYDQKNLIMATYGPYWRNMRKLCTLKLLSSKKVESFRSMRSAEIGLLVTSLKHEATSSEIVNVSNKVLSLSIDMSCLMVFGNKSVNSDCDHKGFQDVVHEGLKLGALFNIADYIPYIGWLDVQGLEKRMKAVSKVFDEMLEKIIDEHVKVFDKDNQKDFVDVMLAFMQDKEADFSIDRSGIKAIVLDMLVASMDTSSTAVEWTISALLKNPRVMKKLQKELQRVXXVGLDRLVDESDLPNLEYLDMVIKESMRLYPVAPLLIPHEAMEDSIVNGFFIPKTSRIIINSWAIGRDPDSWTDPEIFSPERFIDNSIDLRGQDFQLLPFGSGRRGCPGIQLGLLAVRLIVAQMAHCFDWELPNGISPDDLDMTEEFGLTMPRAKNLFAIPRYRLRN

>Papaver_rhoeas_QZBA_scaffold_2014040

MSSSVAVTWISSLLFFLLLAYYLIHTYISKSRNKLNLLPPGPKGLPIVGNFFMLGEAVHRDLHRLSTKYGPIMYLRLGSHPTIVVSSAEAAELFLKTHDLNFASRPFSAAAKYISYDHKGFFTEYGPYWRNVRKLSTLKLLNHSKIESFGSMRRSEINLLIRSLKDAANLHEVVDITGKIFALNKDMSSLMVFGKKRVESDHDKKSFHEVVQEGMRLAAAPNLAEYIPFVGRFDLQGIEKRMKAVSKVYDKMLDKIIDEHVEVFDKDNLKDFIDVLLDCMASNDTEFSIGRSNIKAIALDILLGSMDTTATAIDWTLTELIKNPRVMRKFQDELEREVGLDRLVEESDIPKLEYLDMVVKETFRLHPVAPLLLPHESIENCQVNGFFIPKKSRVIINSWAIGRDPQTWTDPEKFMPERFMGKDIDVRGRDFQLLPFGSGRRGCPGLSLGYLVVRLVVAQLVHCFDWEVPNNMSPDDLDTNEAFGFVMPRAKHLLVIPKYRLNN

>Papaver_somniferum_MIKW_scaffold_2108937

MSSSIAVTWISSLLFFLLLAYYVTRTYIFKSRNKLNPLPPGPKGLPIVGNFFMLGDAVHRDLHRLSTKYGPIMCLRLGFLPTIVVSSAEAAELFLRTHDLNFATRPFNAAAKYISYDNKGFFTEYGPYWRNVRKLSTLKLLNHNKIESFGSMRCSEIELLIKSLKDAANLHEIVDVTSKIFALNKDMSSLMVFGKKRVESDHDKKSFHEVVQEGMRLAAAPNLAEYIPFLGRFDLQGIVKRTKAVSKVYDDMLDKIIDEHVEVFDKDNLKDFTDVLLDCMASNDTEFPIGRSNIKAIALDILLGSMDTTATAIDWTLTELIKNPRVMQKVQDELAREAGLHRLVEESDLPKLEYLDMVIKESFRLHPVAPLLLPHESIEDCQVNGFFIPKRSRVIINSWAIGRDPQTWTDPEKFMPERFMGKDIDVRGRDFQLLPFGSGRRGCPGLSLGYLVVRLVVAQLVHCFDWELPNNMSPDDLDTNEAFGIVMPRAKHLLVIPKYRLHS

>Papaver_bracteatum_ZSNV_scaffold_2110785

MSSSIAVTWISSLFFFLLVAYYLIHTYISKSRNKLNPLPPGPKGLPIVGNFFMLGEAVHRDLHRLSTKYGPIMYLRLGSLPTIVVSSAEAAQLFLKTHDLNFASRPFSAAAKYISYDHKGFFTEYGPYWRNVRKLSTLKLLNHTKIESFGPMRSSEIELLITSLKDAANLHEIVDITSKIFALNKDMSSLMVFGKKRVESDHDRKSFHEVVQEGMRLAAAPNLAEYIPFVGRFDLQGIVKGMKAVSRVYDDMFDKIIDEHVEVFDKDDLKDFIDVLLDCMASNDTEFSIGRSNIKAIALDILLGSVDTTATAIDWTLTELIKNPRVMRKVQDELEREAGLDRLVEESDLPKLEYLDMVIKETFRLHPVAPLLLPHESIEDCQVNGFFIPKKSRVIINGWAIGRDPQTWTDPEKFMPERFMGKDIDVRGRDFQLLPFGSGRRGCPGLSLGYLVVRLVVAQLVHCFDLELPNNMSPDDLDTDEAFGFVMPRAKHLLVIPKYRLNS

>Aconitum_carmichaelii_CNA0013858_125611

MKIMDSWIPAILFTIITCTSSISYLLFRTKARKCLPPSPPGLPLIGHLHLLGELPHHTLQKLAKKYGPIMSLRLGFVPAIIVTSPEYAELFLKTHDLVFASRPKLQAADYISYGQKNLAFAKYGPYWRNIRRLCTVELLSSTKIEVFMGMRKEEVTVFVKSMKDASVAGCALDVSTKIESLIEDMTYRLVLGKKEDLLNFKSGLKEAMRLAGLFNIADYVPSLGAFDLQGLKPRMAAVTKVLDGFLEKIIDEHVYDAKELNGRHRDFVDVMLSLMKSNNERELHIERDHIKAIVLDMLAATMDTSSTAIEWVIAELLKHPRVMKLVQQELETVVGLDRMVEEADLIKLDYLKLVIKESMRIHPVAPLLIPHESMEDITINGYFIPKKSRVLVNTWAIGRDTNVWSDNAEEFCPERFVGTDIDIQGHDFRLLPFGSGRRKCPGMQLGMTVVQIIVAQLVHCFDLDLPDGMSPEELDMTEKFGLTLPRANHLQAIPTYRLHINL

>Sorbus_auluparia_CYP736A107

MALWIWATIGVLALVHVLQTWISKGKNKNKMLPPGPRGFPIFGSLHLLGKVPNRDLYQLAQKYGDIMYMRLGLHHNVVISSSRAAELFLKTHDLTFASRPPHEGAKHIIFGQKNMSFAEYGSYWRDMRKMCMLELLSNHKINSFKAMRREEVSLLIQSVQEDANNRRVPINLSAKISSLGVDLTCRMVFGKKYKDEEFDERGFTAVMKEAIQLVAAPNLNDHIPFLAPLDLQGLTKKMKAVNKAFDAFFEKIIDDHLQSKDEERAKDFIDVMLGHLGSEGSDYRIERVHIKALMLDMFAAAVDTPSTAILWALSELLRHPQVMKKVQKELENVVGLNRMVEESDLEKLEYLDMVVKETLRLHPVVPLLLPHLSIEDCIVDGYHIPKNSRLIINAWAIGRDPSAWEDAEKFVPERFEGSDIDVKGNHFQLVPFGSGRRRCAGMQLGLTVIQFVLAQIVHCFDWELQDNMLPNELDMTEVFGLVVSRTKDLLLIPSYRLQN

>Malus_domestica_CYP736A163

MALWIWATVGVLALVHVLQTWISKGKNKNKKLPPGPRGFPIFGSLHLLGKVPNRNLYQLAQKYGDIMYMRLGLHHNIVISSPRAAELFLKTHDLTFASRPPHEGAKHVIFGQTNMSFAEYGPYWRDMRKMCMLELLSNHKINSFKAMRREEVSLLIQSVQEDANNRRVPINLSDKIASLGVDLTCRLVFGKKYKDVEFDERGFTAVMKEAIQLVAAPNLNDHIPFLAPLDLQGLTKKMKAINKAFDAFFENIIDDHLQSKDEERTKDFVDVMLGHMGSEESDYRIERVHVKALMLDMFAASVDTPSTVILWAFSELLRHPQVMKKLQKELENVVGLNRMVEESDLEKLEYLHMVVKETLRLHPAVPLLLPHLSIEDCTVDGYHIPKNSRLIINAWAIGRDPSAWEDAEKFVPERFEGSNIDVKGKHFQLIPFGSGRRRCAGMQLGLTAIQFVLAQIVHCFDWELPDNMLPNELDMTEVFGLVVSRSKDLLLIPSYRLQN

>Santalum_album_CYP736A167

MSPATAVILTLLVALGLSILLRRRQKRNNLPPGPPALPIIGNIHILGTLPHQSLYNLAKKYGPIMSMRLGLVPAVVISSPEAAELVLKTHDIVFASRPRLQVADYFHYGTKGVILTEYGTYWRNMRRLCTVKLLNTVKIDSFAGTRKKEVASFVQSLKEASVAHKMVNLSARVANVIENMVCLMVIGRSSDERFKLKEVIQEAAQLAGAFNIGDYVPFLMPLDLQGLTRRIKSGSKAFDDILEVIIDEHVQDIKDHDDEQHGDFIDVLLAMMNKPMDSREGLSIIDRTNIKAILVDMIGAAMDTSTSGVEWAISELIKHPRVMKKLQDEVKTVIGMNRMVEEADLPKLPYLDMVVKETMRLHPPGPLLVPRESMEDITINGYYIPKKSRIIVNAWAIGRDTNAWSNNAHEFFPERFMSSNVDLQGQDFQLIPFGSGRRGCPGMRLGLTTVRLVLAQLIHCFDLELPKGTVATDLDMSEKFGLAMPRAQHLLAFPTYRLES

>Origanum_vulgare_CYP736A300

MEWFWAALSLIVFLSLLHQLLKKEKKREANLPPSPIALPVIGHLHLLGKNLPLKLHAIAERHGPIVFLRLGLVRALVVSTAAGAELVLKTHDLVFSGRVHHQASRYLGYDQKNIVFAPYGAYWRNMRRLCMVKLLNAAKINEFRPVRRAELEETVASMRRAAEERGVVDVSAVISGVIGDMNSLMVFGRKYVDRDLDEELGFKAVIDEMLHVGALPNLGDFFPFMAALDLQGLDRRMKELSKIFDGFLERIIDDHLLKKTENTKKGDFVDTMLAVMEAGEADFEFDRRHVKAVLLDMLIAGMDTSASTVEWALSELIRHPEITKKLQKELEQVVGMDQMVDESHLDKLDYLDSVLKETLRLYPPGRLLVHETMEECTVNGFHIPKGTWTFVNMWSIGRDPAMWHEPEKFVPERFAGENLDFLGQNFKFIPFGAGRRSCPGLQLGLTFVRLVLAQLVHCFDWELPNGMVPSDLDMNEKFGIVTSRDKHLMAIPTYRLNK

>Thuja_plicata_KP004988

MDLNMSVPAIGLIIFLFTVLSSILLGRARKNKGRLSLPGPFPLPIIGNLHQLGALPHRGLQRLAEKHGPIMAVKLGSVPVVVASSSQTAKQFLKTHDLSFASRPSNASGKYMFYNRQDIVFAPYGAYWRNMRKICMMELLNGQRVESFRGDREEAAVSMVRSIWEKSQQGRVGVDLSHSVACYTSDLMWRILTGRTKADGVSGGSEFEELLWEGNEVIGAIDVGDFIPWLEWLDLQGLRRRMKNVFQRLDAVFDKIIDEHVERKGRRTVGEEEQHKDLIGVLVDMDNITDQDRKGIIMDMFLAGIETSASTVDWAMSELLRNPHVMKKLQEEIDFIVKKDEKITASHIVSMEYLHCVVKETMRLYPVGPLLIPHESIKDCVVEGPHQDYFIPTNTRVIVNAWAIGRDPKVWEDPLEFRPERFMGRNFDIIRDPELSMIPFGAGRRSCPGASMAIANMEIALAHLVSYFNWKCEGELDMRESFGINIPRKVHLFAIPTSRLRGNEPFNLKI

>Consolida orientalis_BX5

MKLFKVKQLPVCFKTFLHKLNSMAPSSFSWAWPCAVFMLVAYFVYLLRKKSSLCLPPGPKGLPIVGNLFMLGALPHRVLHRFAQQYGPIMHLQLGFIHTIVVSSPQAAELFLRTHDRIFSGRPYNQAAGYISYDTKGLTFSSYGSYWRNTRRVCALELLNHLKIDAFKHVRTEEMNLFIESLKEASTAKVAVDLSTKLKGLNTDISFRMVLGKKLVHRADQEKFSAVVHEGMHLAAFFNIADYFPYVGALNLQGKMKRIKAVHRFYDDILDKIIEEHVRVGPGGGTPPFIDMMMSYMESKENDFLKDRSNVKAIVLDMLLGAMDTASTAIEWVMAELIKHPSLMKKVRQEVDGVVGLDRMLDESDLVHLEYLDMVIKETFRVHPVAPLLIPHSSMEDCTVNGYQIPRNSRIIINTFAIGHDPEAWPDPDEFKPERFVGKNMDYKGSAFQFLPFGSGRRGCPGITMGVRVVRLVVAQLVHCFDWELPNGMVCTELDMDEDFGLTVARANPLLVIPTYRLRI

>Catharanthus roseus_CYP72A1v1

MEMDMYTIRKAIAATIFALVVAWAWRVLDWAWFTPKRIEKRLRQQGFRGNPYRFLVGDVKESGKMHQEALSKPMEFNNDIVPRLMPHINHTINTYGGNSFTWMGRIPRIHVMEPELIKEVLTHSSKYQKNFDVHNPLVKFLLTGVGSFEGAKWSKHRRIISPAFTLEKLKSMLPAFAICYHDMLTKWEKIAEKQGSHEIDIFPTFDVLTSDVISKVAFGSTYEEGGKIFRLLKELMDLTIDCMRDVYIPGWSYLPTKRNKRMKEINKEITDMLRFIINKRMKALKAGEPGEDDLLGVLLESNIQEIQKQGNKKDGGMSINDVIEECKLFYFAGQETTGVLLTWTTILLSKHPEWQERAREEVLQAFGKNKPEFERLNHLKYVSMILYEVLRLYPPVIDLTKIVHEDTKLGPYTIPAGTQVMLPTVMLHREKSIWGEDATEFNPMRFADGVANATKNNVTYLPFSWGPRVCLGQNFALLQAKLGLAMILQRFTFDVAPSYVHAPFTILTVQPQFGSHVIYKKLES

**SI Fig. 10, BX8 phylogeny**

>Nicotiana tabacum_UGT71A6

MKTTELVFIPAPGMGHLVPTVEVAKQLVDRDEQLSITVLIMTLPLETNIPSYTKSLSSDYSSRITLLQLSQPETSVSMSSFNAINFFEYISSYKDRVKDAVNETFSSSSSVKLKGFVIDMFCTAMIDVANEFGIPSYVFYTSNAAMLGLQLHFQSLSIEYSPKVHNYLDPESEVAISTYINPIPVKCLPGIILDNDKSGTMFVNHARRFRETKGIMVNTFAELESHALKALSDDEKIPPIYPVGPILNLGDGNEDHNQEYDMIMKWLDEQPHSSVVFLCFGSKGSFEEDQVKEIANALERSGNRFLWSLRRPPPKDTLQFPSEFENPEEVLPVGFFQRTKGRGKVIGWAPQLAILSHPAVGGFVSHCGWNSTLESVRSGVPIATWPLYAEQQSNAFQLVKDLGMAVEIKMDYREDFNKTNPPLVKAEEIEDGIRKLMDSENKIRAKVMEMKDKSRAALLEGGSSYVALGHFVETVMKN

>Medicago truncatula_UGT72B78

MEYQQQSRTTPKQELPPLVAMFPTPGMGHLIPMVEFAKRLSKHNLPITFIIPNDGPPSKAQTTVLTSLPHGISHVFLPPVTLSDLPPNTKPEPLMIATILRSLPSLHQTLLSLMTSHRLSALIIDLFGTDAFDMAAELDIPSYLYFPSTANMLSFAFYFPQLDQKVQGEFRDILEPLNIPGCFAVHGKDLPDPVQDRKDEAYKCFLHQMTRHRLAKGIIENSFFELEPEAITFLQKNEPPVYPVGPLVNEDSINNGSEFDMCFQWLDEQPRGSVIYVNFGSEGILTSAQTDEIAYGLEMSEQRFLWVLRCPKDKVENDSNFIANSNVDPFEFLPNGFTERTKGKGLVLPYWAPQAQVLSHISIGGFVSHCGWNSTLESVVNGVPLIAWPLYAEQKMNAVLLSENVKVAIRPKVGENGLVQREEIASVVKRLMVGEEEQKIRYRMKDLKEAAINALKENGSSTKQICELALKWKGVTIPN

>Solanum tuberosum_UGT73Q21

MDKRSDQLHIYFLPMMAPGHMIPLVDIARQFARHGVKATIITTPLNASKFSKTIQRDREMGSDISIRTVKFPCKEAGLPEGCENIASTTSTLMYLNFIMGLSLFQNPIEQFLEEDHPDCLIAAPQFSWAVDVATKLEIPTLAFNGSGFFPLCALHSLMEHKPHLNVESEMEEFVIPGLPDTIKMSRQKLSEHLMDEKDTPVTAIVKAIMRAETTSYGVIVNSFYEMEPNYVKHSREVVGRKVWHVGPISLCNEDKSQRGQDSSFCEQKCLDWLDTKEPKSVIYICFGSMAVFSSAQLLEIAIALEASNQQFIWAVTQNTINEEQNEWIPEGFEEKLNVNGRGLIIKGWAPQVLILDHEAIGGFVTHCGWNSLLEGVSAGVPMITWPLSAEQFFNEKLLVEILKIGVPVGSEAWSNRTDSTVPINRKDIERAVTKVMVGQEAEEMRGRAAALGELAKRAVEKGGSSHNSLISLLEQLRNAKITSN

>Zea mays_UGT74CA13

MVGEESGCGVEGGTGGGEAPVASAPNGDRSGNSRLAVPSSSADDRGFHRSSTMPGVIKNDEITNESTGPSNLERSRTERRRQNNPAIDPAKQLLDERIPIKKKLKMLNRIATVKDDGTVVVDVPSGLEPTIVGGTEDIYTEAVEEALDGTEILYRPPMQIVILIVGTRGDVQPFVAIGKCLQDYGHRVRLATHANFKEFVLTAGLEFFPLGGDPKILAEYMVKNKGFLPSGPSEIPIQRKQMKEIIFSLLPACKEPDPDTGIPFNVDAIIANPPAYGHTHVAEALKVPIHIFFTMPWTPTNEFPHPLSRVKQPAGYRLSYQIVDSMIWLGIRDMINEFRKKKLKLRPVTYLSGSQGSGNDIPHGYIWSPHLVPKPKDWGPKIDVVGFCFLDLASNYVPPEPLVEWLEAGDKPIYVGFGSLPVQDPQKMTEIIVKALEITGQRGIINKGWGGLGTLSEPKDFVYLLDNCPHDWLFLHCKAVVHHGGAGTTAAGLKAACPTTIVPFFGDQPFWGDRVHARGLGPPPIPVDQFGLQKLVDAITFMMKPE

>Arabidopsis thaliana_UGT76E2

MEEKQVKETRIVLVPVPAQGHVTPMMQLGKALHSKGFSITVVLTQSNRVSSSKDFSDFHFLTIPGSLTESDLQNLGPQKFVLKLNQICEASFKQCIGQLLHEQCNNDIACVVYDEYMYFSHAAVKEFQLPSVVFSTTSATAFVCRSVLSRVNAESFLIDMKDPETQDKVFPGLHPLRYKDLPTSVFGPIESTLKVYSETVNTRTASAVIINSASCLESSSLARLQQQLQVPVYPIGPLHITASAPSSLLEEDRSCVEWLNKQKSNSVIYISLGSLALMDTKDMLEMAWGLSNSNQPFLWVVRPGSIPGSEWTESLPEEFNRLVSERGYIVKWAPQMEVLRHPAVGGFWSHCGWNSTVESIGEGVPMICRPFTGDQKVNARYLERVWRIGVQLEGDLDKETVERAVEWLLVDEEGAEMRKRAIDLKEKIETSVRSGGSSCSSLDDFVNSM

>Vitis vinifera_UGT78A23

MTTTASSMDRHVAVLGFPTHTATLLKLLRGLASAAPTTIFSFFNTPKANSSISSAQSPHGIHNLRVYDVADGVPEDLVLSANPLARIEMFLKATPGNFRDALEVAEKDIGRKISCLVSDVFLWFTADMAEEMGVPWVAIRTAALYSLSVHIYTDAIREAVGVAGQVQDQTLDFIPGFSAIKVEDLPEGMVFGDTESPFACMLHKMGLMLPRATIVATNSFEELEPTIVTNDLKSKLQKVLTVGPFDLSSPPPLILDASGCLPWLDNKKEASVAYVSFGSIATPPPNEIVALAEALEATGIPFLWSLREHAMNNLPKGFLERTTAHGKVVSWAPQPQVLAHASVAVFITHSGWNSVTESIVGGVPMICRPFFGDQRLNRRMVQDVWGIGIGVEGGILTKRGVMSALGLILSHEGKKMREKIGVLKELARRAVEPNGSSTQNLGHLLEVITTSKLPLDTNK

>Arabidopsis thaliana_UGT79B1

MGVFGSNESSSMSIVMYPWLAFGHMTPFLHLSNKLAEKGHKIVFLLPKKALNQLEPLNLYPNLITFHTISIPQVKGLPPGAETNSDVPFFLTHLLAVAMDQTRPEVETIFRTIKPDLVFYDSAHWIPEIAKPIGAKTVCFNIVSAASIALSLVPSAEREVIDGKEMSGEELAKTPLGYPSSKVVLRPHEAKSLSFVWRKHEAIGSFFDGKVTAMRNCDAIAIRTCRETEGKFCDYISRQYSKPVYLTGPVLPGSQPNQPSLDPQWAEWLAKFNHGSVVFCAFGSQPVVNKIDQFQELCLGLESTGFPFLVAIKPPSGVSTVEEALPEGFKERVQGRGVVFGGWIQQPLVLNHPSVGCFVSHCGFGSMWESLMSDCQIVLVPQHGEQILNARLMTEEMEVAVEVEREKKGWFSRQSLENAVKSVMEEGSEIGEKVRKNHDKWRCVLTDSGFSDGYIDKFEQNLIELVKS

>Camellia sinensis_UGT85A143

MGSRKQPHAVCVPFPAQGHINPMMQLAKLLHSRGFYITFVNTEFNHRRLLQSKGPEFLKGCADFQFESIPDGLPPSDRDATQDPPTLCIAMRDNCLDPFRVLLKKLNNNNNSIASRQVPGVTCVVSDGAMNFAMKAAEEAGIPEVQFWTASACGFMGYLHYPQLVQRGIFPFKDESFQSDGSLDTTIDWIPGMRNIRLKDMPSFIRTTDPNDILFNYLSEEVQNCLKASAIIFNTFDTLEHQVLQAIASKFHNIYTIGPLSLLSKQVIDGEFKSLNSSLWKEDTKCLQWLDTKEPNSVVYVNYGSITVMTDQHLKEFAWGLANSKHPFLWIVRPDIVMGDSAILPEHFVEETKDRGLLVSWCPQEQVLSHPSIGVFLTHCGWNSTLESICGGVPIICWPFFAEQQTNCRYACTEWGIGMEVNHDVKRNEIVALINEMLEGDKGKQMRKKALKLKKEAEEATDVGGLSYNNFDRLIKEALHYCEQY

>Co_23

MDSDSKPHAVCIPFPAQGHINPMTKLAKILHHKGFHITFVHTEYNYKRLLKARGLLIELPRDFVFKTIPDGLPPPPDENIDATQDIPDLCVSTSKNCLAPFRNLVADLNNTDSIPPVSCIVSDGVMTFTLQVAQELGVPQVLFWTTSACGFMGYLQFPRLVQDGIIPFKDISHTSNDYLETPVDWIPGVDNIRLRDLPSFIRTTDPNDPLVMHAIGEVERATKASAIILNTFDELERDVLSSIQPILPPVYTIGSLHMLLPQISGDELNYIRSNLWKEEPECLQWLDSKDPNSVVYVNFGSIAVMTPQQTVEFAWGLANSNKSFLWVIRPDLVSGKDAILPSDFMAVTKERGLLASWCPQEQVLSHPSVCGFLTHSGWNSTLESVGSGVPMICWPFFTEQQMNCRYSCVHWGIGMEIESNVKREEVERLVIELMDGKKGEEMKRKAMEWKKKAEAAVAPGGSSYMNIDKLVREILVMKFSNVQIIQQI

>Co_26

MGSELIVDHHHVHVLMVSFPAQGHVNPLLRLAKRLASKGLLATLSSTHNIIHMIQKPTRPTPSLDIGSGQLRFESFSDGWDHDADRTNLNLYVPQLETVGRDSFFELVKKQSESGTPVSCIVSNPFIPWVADVAAELGIPCALLWVQSCSVYSVYFHFLHKLASFPTEEQPNMLVELPGLPTLESDEIPSFLHPVYSPQFRVLKHVILNQFKNLEKTFCVLVDSFEELERETLEPLLGSSTIIRPIGPLCKWSTEEKDVRVDMWTAAEECIEWLDSKPVGSVVYVSFGSVAVLDQKPMEELAKGLMNCGQPFLWVVRAPPKESDQNIGSLPDGFAQEAAGKGLVVQWCPQEKVLAHPSLACFVTHCGWNSSMEILSSGVPAVAAPQWGDQVTNAKFLTDVYNVGVRLLRSENDLKTLSSERVVNCIAEVTNGPRAAEFKTNALKWKKAAEEAALEGGSSYHNLDKFVAEVQALRPGLGTRPHRT

>Co_27

MDHQSKPNHFLLLVFPAHGHINPALELAKRLITTGAHVTFVTCMSSHRSLVKNSTSFPDALTFEPLFHTDDHGFKSADDVDQYLLEMRRGEGAISGLIEKAAGEGRPVTCFVNTVLFPWTFEVVQKYRMFSALLWVQPATAFNIYYYYFNGYRDLIVDRAKDPESVVQLPGLPLFKCRDLPAAVLPSNAHPVLHSFFQEQFNILSTERANFWILMNTFDGLELESMSAVTKFNLKGVGPLIPVAFLNSEHSMDNSSGGDLVDHSKEYLKWLDSQDKSSVVYVSFGSLAALSEEQMEALARALVDTGRPFLWVIRERKEDGKYNEILCGKHKGIVVPWCSQVEVLNHDSVGCFVTHCGWNSTLEAIAMGVPVVTFPQWHNQGTNAKLVVDVWKMGVRPTPNDDDKIVHSEEIMRCLKQVMESEELPKNAKKWKVLARQAVKQEGSSQKNLQKFIEAIGQFYY

>Co_28

MEKGSLLTRPCTMGKINHHDGLHVAVLSFPSGTHAVPLMSLTRRLAAAAPDVSFSFFGTTNSNRSVSSDCNPQNIKVFDVDDGVPEGYVFTGKRQEDTELFLNVVPGNFRKEMEKAVLETGRSISCLMGDAFLWHGADLADELGVSWVPFWTSSASALSAHFHTDFIRDTVGVGHTGREEEALTFVPGMHPSHRVKDLPLEVLDGGDKPMVISRLLHQMAQTLPRAATIIVNSFDTLEPSVTNNFGSKFDNFLMVGPINLTSSSPPDHDPYNCLSWLQDKKPASVAYVSFGTITTPPPKEVAALADALEDSNVFFIWSLKEAQKVNLPRGFVERTKERGMVVPWAPQEKILEHSACGVFVTHCGWNSVLESIGGGVPMICRPYFGDQKMNGRLVSHVWGIGVGVDNGVLSRDGVKECLDVMLKKEEGKVVRKKAEALKQLGEQAVAPDGSSSNNFKTLLNMLCSIN

>Co_24

MDSDSKPHAVCIPFPAQGHINPMTKLAKILHHKGFHITFVHTEYNYKRLLKARGLLIELPRDFVFKTIPDGLPPPPDENIDATQDIPDLCVSTSKNCLAPFRNLVADLNNTDSIPPVSCIVSDGVMTFTLQVAQELGVPQVLFWTTSACGFMGYLQFPRLVQDGIIPFKDISHTSNDYLETPVDWIPGVDNIRLRDLPSFIRTTDPNDPLVMHAIGEVERATKASAIILNTFDELERDVLSSIQPILPPVYTIGSLHMLLPQISGDELNYIRSNLWKEEPECLQWLDSKDPNSVVYVNFGSIAVMTPQQTVEFAWGLANSNKSFLWVIRPDLVSGKDAILPSDFMAVTKERGLLASWCPQEQVLSHPSVCGFLTHSGWNSTLESVGSGVPMICWPFFTEQQMNCRYSCVHWGIGMEIESNVKREEVERLVIELMDGKKGEEMKRKAMEWKKKAEAAVAPGGSSYMNIEKLVREILVI

>Malus domestica_UGT75L17

MVQHRFLLVTFPAQGHINPSLQFAKRLINTTGAHVTYVTSLSAHRRIGNGSIPDGLTYAPFSDGYDDGFKPGDNVDDYMSELRRRGVQAITDLVVASANEGHPYTCLVYSLLLPWSAGMAHELHLPSVLLWIQPATVFDIYYYYFNGYKDLIRDNTSSGTNNVLPCSIELPGLPLSFTSRDLPSFMVDTNPYNFALPLFQEQMELLERETNPTILVNTFDALEPEALKAIDKYNLIGVGPLIPSAFLDGKDPSDKSFGGDLFQKSKDSSYLEWLNSKPEGSVIYVSFGSISVLGKAQMEEIAKGLLDCGLPFLWVIRDKVGKKGDDNEAKKEEEMLRCREELEELGMIVPWCSQVEVLSSPSLGCFVTHCGWNSSLESLVSGVPVVAFPQWTDQGTNAKLIEDYWKTGVRVTPNEEGIVTGEELKRCLDLVLGSGEIGEDVRRNAKKWKDLAREAVSEGDSSDKNLRAFLDQIKVLKDARH

>Co_BX8

MASSSPKTPHIVCVPAPAQGHINPMFKLAKLFHSRGFYITFVHSEFSYQRLLQASALDHLKGLNNFRFETIPDGLPPENKRGVSDVPELCKSMRNTCADPFRSLILKLNSSSDVPPVTCIVADVAMDFTLQVSEELGPPVVLFFTLSGCGVLGYMHYGELLERGYFPLREESFLSNGYLDTEIDWIPAMKGIRLKDLPSFLRTTDPDDIMFNCKIIEVNSAFKAKGVILNTFDDLEQEVLDAIKSKIPQLYTIGPLSMLCDHMLQPDSKLCEASLWEEDTSCLEWLQEKDPKSVLYVNIGSLATMTSQQLGEFAWGLANSMCPFLWVIRPDILDRASGIVSEDYKKEIGGRGLLVSWCQQEKVLKHPSIGGFLTHCGWNSTLESLCEGVPMICWPFFAEQQTNCFYICNKWGIGMEIDFDVKRVEIGMMVKELMKGEKGLEMRNKVEDLMSKAIKATTPGGSSHTNFEMLMEDVAKW

>LgBX8

MSSFSKAESSEQKPHAVFIPAPGQGHINPMLMLAKLLHYNGFHVTFVNTEFIHRRLLKSCGPSALRGLPGFRFATIPDGLPESSTRDVAALCESFTTTCLEPFCDLLLEIKSSGGGVPPVSCIVADGEMSFTLKAAERFGLPEVLFWTTSACGLLGYTQYRRLVEKGYVPLQDMSQGYLDTTVDWVPGIKNIQLRDFPAFIRTTDPKDFMLNFKTQEVEAIPRAKALILNTFDALEQDVLNALSDMFPTIYTVGPLQLMMNHVHDQTLDSITSSLWKEDFQCIEWLDSKEPGSVVYVNFGSITVVTAQQLTEFAWGLANSNKQFLWVIRPDIVAGESALVPPEFVEKTKNRSLLISWCPQEQVLKHPAIGGFLTHSGWNSTLESVINGVPMICWPFFAEQQTNCRFSCVEWEMGMEIDNDVKRDEVEFLVRELMDGEKGKKMKEKAMEWKNKAEAATVPGGSSYLNFEKLIKELLP

>ZmBX8

MAASCGGRVVVFPFPFQGHFNPVMRLARALHARGVGITVFHTAGARAPDPADYPADYRFVPVPVEVAPELMASEDIAAIVTALNAACEAPFRDRLSALLSAADGEAGEAGGRVRCVLTDVSWDAVLSAARGLGVPALGVMTASAATFRVYMAYRTLVDKGYLPVREERKDDAVAELPPYRVKDLLRHETCDLEEFADLLGRVIAAARLSSGLIFHTFPFIEAGTLGEIRDDMSVPVYAVAPLNKLVPAATASLHGEVQADRGCLRWLDAQRARSVLYVSFGSMAAMDPHEFVELAWGLADAGRPFVWVVRPNLIRGFESGALPDGVEDRVRGRGVVVSWAPQEEVLAHPAVGGFFTHCGWNSTVEAVSEGVPMICHPRHGDQYGNARYVCHVWKVGTEVAGDQLERGEIKAAIDRLMGGSEEGEGIRKRMNELKIAADKGIDESAGSDLTNLVHLINSY

>Gentiana triflora_UGT84A9a

MGSLTNNDNLHIFLVCFIGQGVVNPMLRLGKAFASKGLLVTLSAPEIVGTEIRKANNLNDDQPIKVGSGMIRFEFFDDGWESVNGSKPFDVWVYINHLDQTGRQKLPIMLKKHEETGTPVSCLILNPLVPWVADVADSLQIPCATLWVQSCASFSAYYHYHHGLVPFPTESEPEIDVQLPGMPLLKYDEVPDYLHPRTPYPFFGTNILGQFKNLSKNFCILMDTFYELEHEIIDNMCKLCPIKPIGPLFKIPKDPSSNGITGNFMKVDDCKEWLDSRPTSTVVYVSVGSVVYLKQEQVTEMAYGILNSEVSFLWVLRPPSKRIGTEPHVLPEEFWEKAGDRGKVVQWSPQEQVLAHPATVGFLTHCGWNSTQEAISSGVPVITFPQFGDQVTNAKFLVEEFKVGVRLGRGELENRIITRDEVERALREITSGPKAEEVKENALKWKKKAEETVAKGGYSERNLVGFIEEVARKTGTK

>Arabidopsis thaliana_UGT76D1

MAEIRQRRVLMVPAPFQGHLPSMMNLASYLSSQGFSITIVRNEFNFKDISHNFPGIKFFTIKDGLSESDVKSLGLLEFVLELNSVCEPLLKEFLTNHDDVVDFIIYDEFVYFPRRVAEDMNLPKMVFSPSSAATSISRCVLMENQSNGLLPPQDARSQLEETVPEFHPFRFKDLPFTAYGSMERLMILYENVSNRASSSGIIHNSSDCLENSFITTAQEKWGVPVYPVGPLHMTNSAMSCPSLFEEERNCLEWLEKQETSSVIYISMGSLAMTQDIEAVEMAMGFVQSNQPFLWVIRPGSINGQESLDFLPEQFNQTVTDGRGFVVKWAPQKEVLRHRAVGGFWNHGGWNSCLESISSGVPMICRPYSGDQRVNTRLMSHVWQTAYEIEGELERGAVEMAVRRLIVDQEGQEMRMRATILKEEVEASVTTEGSSHNSLNNLVHAIMMQIDEQ
